# Chemotherapy confers a conserved secondary tolerance to EGFR inhibition via AXL-mediated signaling bypass

**DOI:** 10.1038/s41598-021-87599-9

**Published:** 2021-04-13

**Authors:** Mark Borris D. Aldonza, Roben D. Delos Reyes, Young Seo Kim, Jayoung Ku, Ana Melisa Barsallo, Ji-Young Hong, Sang Kook Lee, Han Suk Ryu, YongKeun Park, Je-Yoel Cho, Yoosik Kim

**Affiliations:** 1 Department of Chemical and Biomolecular Engineering, Korea Advanced Institute of Science and Technology (KAIST), Daejeon, 34141 Korea; 2 Department of Biological Sciences, KAIST, Daejeon, 34141 Korea; 3 KI for Health Science and Technology (KIHST), KAIST, Daejeon, 34141 Korea; 4 Department of Biochemistry, College of Veterinary Medicine, Seoul National University, Seoul, 151-742 Korea; 5 BK21 PLUS Program for Creative Veterinary Science Research and Research Institute for Veterinary Science, Seoul National University, Seoul, 151-742 Korea; 6 Department of Electrical Engineering, KAIST, Daejeon, 34141 Korea; 7 Tomocube Inc, Daejeon, 34051 Korea; 8 College of Pharmacy, Natural Products Research Institute, Seoul National University, Seoul, 08826 Korea; 9 Department of Physics, KAIST, Daejeon, 34141 Korea; 10 Department of Pathology, Seoul National University Hospital, Seoul National University College of Medicine, Seoul, 03080 Korea

**Keywords:** Cancer, Chemical biology, Computational biology and bioinformatics, Drug discovery, Biomarkers, Oncology

## Abstract

Drug resistance remains the major culprit of therapy failure in disseminated cancers. Simultaneous resistance to multiple, chemically different drugs feeds this failure resulting in cancer relapse. Here, we investigate co-resistance signatures shared between antimitotic drugs (AMDs) and inhibitors of receptor tyrosine kinases (RTKs) to probe mechanisms of secondary resistance. We map co-resistance ranks in multiple drug pairs and identified a more widespread occurrence of co-resistance to the EGFR-tyrosine kinase inhibitor (TKI) gefitinib in hundreds of cancer cell lines resistant to at least 11 AMDs. By surveying different parameters of genomic alterations, we find that the two RTKs EGFR and AXL displayed similar alteration and expression signatures. Using acquired paclitaxel and epothilone B resistance as first-line AMD failure models, we show that a stable collateral resistance to gefitinib can be relayed by entering a dynamic, drug-tolerant persister state where AXL acts as bypass signal. Delayed AXL degradation rendered this persistence to become stably resistant. We probed this degradation process using a new EGFR-TKI candidate YD and demonstrated that AXL bypass-driven collateral resistance can be suppressed pharmacologically. The findings emphasize that AXL bypass track is employed by chemoresistant cancer cells upon EGFR inhibition to enter a persister state and evolve resistance to EGFR-TKIs.

## Introduction

Emergence and spread of drug resistance in cancer necessitate the immediate discovery of novel treatment approaches. Biological mechanisms of drug resistance implicate a wide range of acquisition models from Darwinian selection^1^ to a non-heritable, random transcriptional variability^2,3^. Genetic mutations have been the paradigmatic cause of drug resistance in many cancers. While many targeted therapies are able to eradicate most of the disseminated tumors carrying these targetable genetic backgrounds, a small subset of surviving cancer cells develop resistance which can be driven non-genetically^3^. Independence from bona fide genetic drivers of resistance is associated with the drug-tolerant persister state, a phenomenon whereby small subpopulations of cells survive strong drug challenges in short-term treatment (days) via tolerance, in which the growth rate becomes stagnant and remains to have no appreciable growth in continued long-term treatment (weeks to months). However, a fraction of these dormant persisters acquires the capacity for population expansion in the presence of the drug^3^.

It has been experimentally demonstrated that resistance can evolve from a persister bottleneck. Yet, many questions remain as to how the passage through this persistence state influences the trajectories to a more stable resistant phenotype. Does drug persistence require a specific biological logic (genetic or epigenetic) based on particular drug/s? Can drug-resistant cancer cells re-enter a persister state when challenged with different drug/s to acquire a multidrug-resistant phenotype?

In the clinic, the recurrence of tumors after the failure of the first-line therapy is often managed independent of knowledge on sensitivity or resistance trajectories—the stochastic rendering of collateral trade-offs from the first drug to the second^4^. These fitness trade-offs often restrict the evolution of resistance, as previously reported in the context of antibiotic resistance^5^ and anticancer drug resistance^4^ whereby the induction of rugged fitness landscapes by these trade-offs impeded the number of trajectories to a higher fitness or confined the evolution to become irreversible. Collateral (or cross) resistance that arises from resistance to the first-line therapies spurred efforts to exploit the concept of an evolutionary trade-off, wherein the development of resistance toward a drug or drugs imparts susceptibility to other drugs. This emerging strategy stimulated previous work on pharmacological screens in chemoresistant cancer cells^6^, high-throughput in vitro evolution experiments^7^, and evolutionarily informed drug combinations^8^ in an attempt to build a network of collateral sensitivity. However, identifying and depending on these trade-offs (i.e., collateral sensitivity) for resistance information have recently been challenged because of the potential existence of a multi-dimensional evolutionary saddle point in the fitness landscape inducing divergent selection potential and differential collateral response^9,10^. Thus, a shift to ‘collateral likelihood’ of sensitivity between drugs derived from evolutionary experiments with many replicates is suggested to be more informative rather than a collateral sensitivity reported from a small number of evolutionary replicates^9^. As multiple-type sequential drug cycles are frequently prescribed in the clinic, it is critical to consider the likelihood of collateral resistance or sensitivity trajectories following the first-line therapy failure.

Using the Genomics of Drug Sensitivity in Cancer (GDSC) as a benchmark data set and drug perturbation assays, we report a targetable alternative mechanism that compensates for signaling inhibition and drives a secondary resistance (i.e., EGFR-TKI) following failure to the first-line chemotherapy (i.e., antimitotic drugs). This is critical given that both first-generation EGFR-TKIs gefitinib and erlotinib have since been widely used as second-line or maintenance treatment after chemotherapy failure in advanced non‐small cell lung cancer as opposed to a first-line treatment in chemotherapy-naïve patients^11^. Thus, our study can serve as a basis for poor-responders in this treatment setting. This information will be useful for the development of targeting strategies (i.e., drug combinations) to combat drug-specific resistance evolution and improve the second-line or maintenance setting of cancer treatment.

## Results

### Large-scale analysis of co-occurring resistance between RTK inhibitors and chemotherapy in the GDSC data set

**Figure 1:**
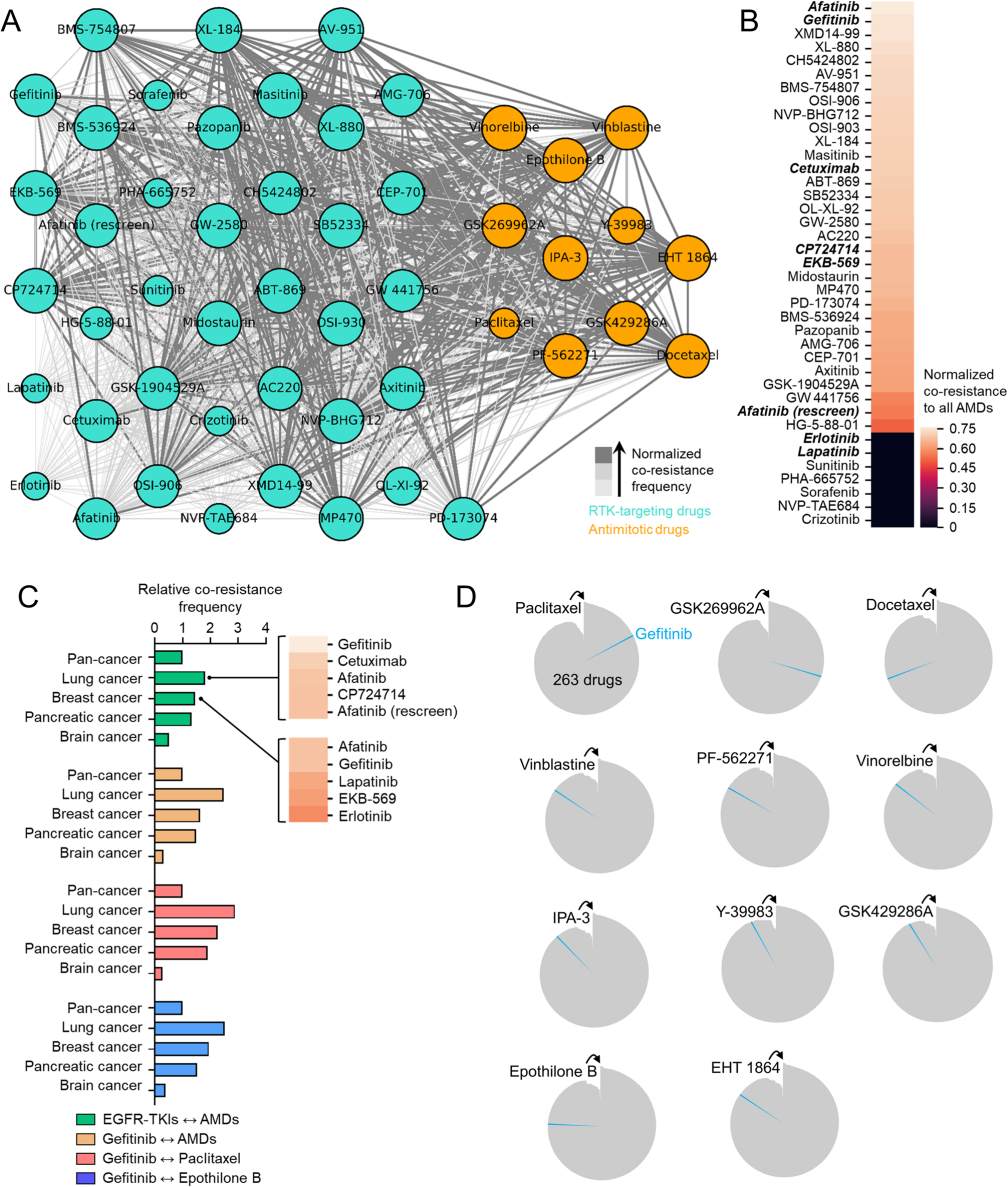
Co-resistance network analysis identifies drug-specific resistance trajectories. (**a**) Network visualization of co-resistance between RTK-targeting drugs and antimitotic drugs (AMDs) processed from the GDSC (https://www.cancerrxgene.org/). The size of each node (encircled drug; RTK inhibitors in cyan and AMDs in orange) relatively corresponds to the number of resistant cancer cell lines. The size and color of edges (connecting lines) represent normalized co-resistance frequency arranged in increasing order (< 10%, < 60%, and ≥ 60%). Python (seaborn; https://seaborn.pydata.org/) was used to generate the plot. (**b**) Ranking of all RTK-targeting drugs based on their normalized co-resistance frequency with all AMDs screened in this study. Highlighted text represents EGFR-TKIs. Python (seaborn; https://seaborn.pydata.org/) was used to generate the plot. (**c**) Co-resistance frequency between the indicated EGFR-TKIs and AMDs per lineage of cancer cell lines processed from the GDSC. Values are relative to the pan-cancer lineage (total of 23 cancer types analyzed per co-resistance case). GraphPad Prism 7.01 was used to generate the plot. (**d**) Radial histogram visualization of co-resistance ranks of gefitinib (highlighted in blue) out of 263 drugs tested in AMD-resistant cancer cell lines from the GDSC (total number of cell lines vary depending on available number of resistant cell lines per AMD). R^59^ (ggplot2; https://ggplot2.tidyverse.org/) was used to generate the plot.

Because resistance signatures can be shared by a wide-array of chemically different drugs, we explored the GDSC, a publicly available data set of pharmacogenomics in cancer cell lines, to examine co-occurring resistance to 265 clinically relevant anti-cancer drugs evaluated as a measure of drug response of 1001 cell lines^12^. Expanding from our previous analysis^13^, we set out to explore the co-resistance signatures shared between two different classes of drugs: RTK inhibitors and antimitotic drugs. These two classes represent clinically available combination regimens employed in various sequential or alternating cancer treatments^11,13–15^. Curating all co-resistance signatures between these compounds (denoted using ↔ such as ‘one RTK inhibitor ↔ one antimitotic drug’) across all cell lines in the database, we derived, clustered, and normalized co-resistance frequencies between the matched drugs and categorized them in increasing degrees (Fig. 1a and Supplementary Fig. S1a). In all RTK inhibitors evaluated, we highlighted the co-resistance signatures of EGFR-TKIs with AMDs (Fig. 1b), wherein gefitinib ranked among the highest AMD-co-resistant TKIs. So far, these co-resistance signatures included data from a pan-cancer dataset, which could cause the co-resistance to include ‘misleading’ data points (i.e., profiling of a drug response in non-relevant cancer background). Therefore, we analyzed the cancer specificity of the co-resistance profiles. We found that co-resistance between gefitinib and AMDs (i.e., gefitinib ↔ paclitaxel or gefitinib ↔ epothilone B) can serve as a proxy to represent co-resistance between all EGFR-TKIs and AMDs, at least based on the similar co-resistance frequencies in lung, breast, and brain cancers (Fig. 1c). We also showed that there is a considerably significant co-resistance trajectory to the EGFR-TKI gefitinib in cell lines classified as resistant to 11 cytoskeleton-targeting antimitotic drugs (CTDs), representing a sub-class of AMDs. It is noteworthy that co-resistance to gefitinib is positioned in varied ranks with > 83% of co-resistance status with associated CTDs out of 265 drugs evaluated in order of CTDs-resistant cancer cell lines (Fig. 1d). Out of these 11 common CTDs, paclitaxel appeared to have the highest rank in sharing a co-resistance signature with gefitinib. However, it should be noted that in the tested panel of cell lines, paclitaxel has the fewest number of classified resistant lines, owing to the high probability rank of co-resistance shared with gefitinib. Resolving this bias, we checked the discretization value (log IC50/cell lines) for each drug in the database. Epothilone B, docetaxel, and paclitaxel have the lowest discretization threshold among the CTDs (all lower than -5). Looking into individual cases, gefitinib increased its rank score in the panel of paclitaxel- and epothilone B-resistant cell lines when chemically similar drugs to gefitinib were removed from the panel of drugs ranked (Supplementary Fig. S1b).

Curious as to how each co-resistance case (gefitinib ↔ CTD) can be characterized and differentiated by its association with a specific RTK target expression, we inferred the extent to which gene expression of 22 RTKs is associated with each of the 11 co-resistance cases. Normalized scores were derived from the basal gene expression profiles of each cell line with similar resistance denomination for two indicated drugs. While co-resistance case-specific variations can be observed, all of the 11 gefitinib ↔ CTD co-resistance cases displayed similar association profiles with expression of the 22 RTKs (Supplementary Fig. S2). This supports the idea that at some degree all gefitinib ↔ CTD co-resistance cases can be driven by a common mechanism that might be regulated at the RTK level.

Given that these resistance correlations exist in a large-scale pharmacogenomics study, it is important to validate and reproduce these correlations in the context of resistance evolution in cells through pharmacological perturbation and selection. Thus, one of our aims is to ascribe significance to correlation between two (or more) specific drugs conferring specific resistance trajectories in a setting where regulatory effects of the tumor microenvironment can be presumed inconsequential to avoid its confuting impact on resistance.

### Genomic alterations associated with co-resistance between an EGFR-TKI and 11 antimitotic drugs

**Figure 2:**
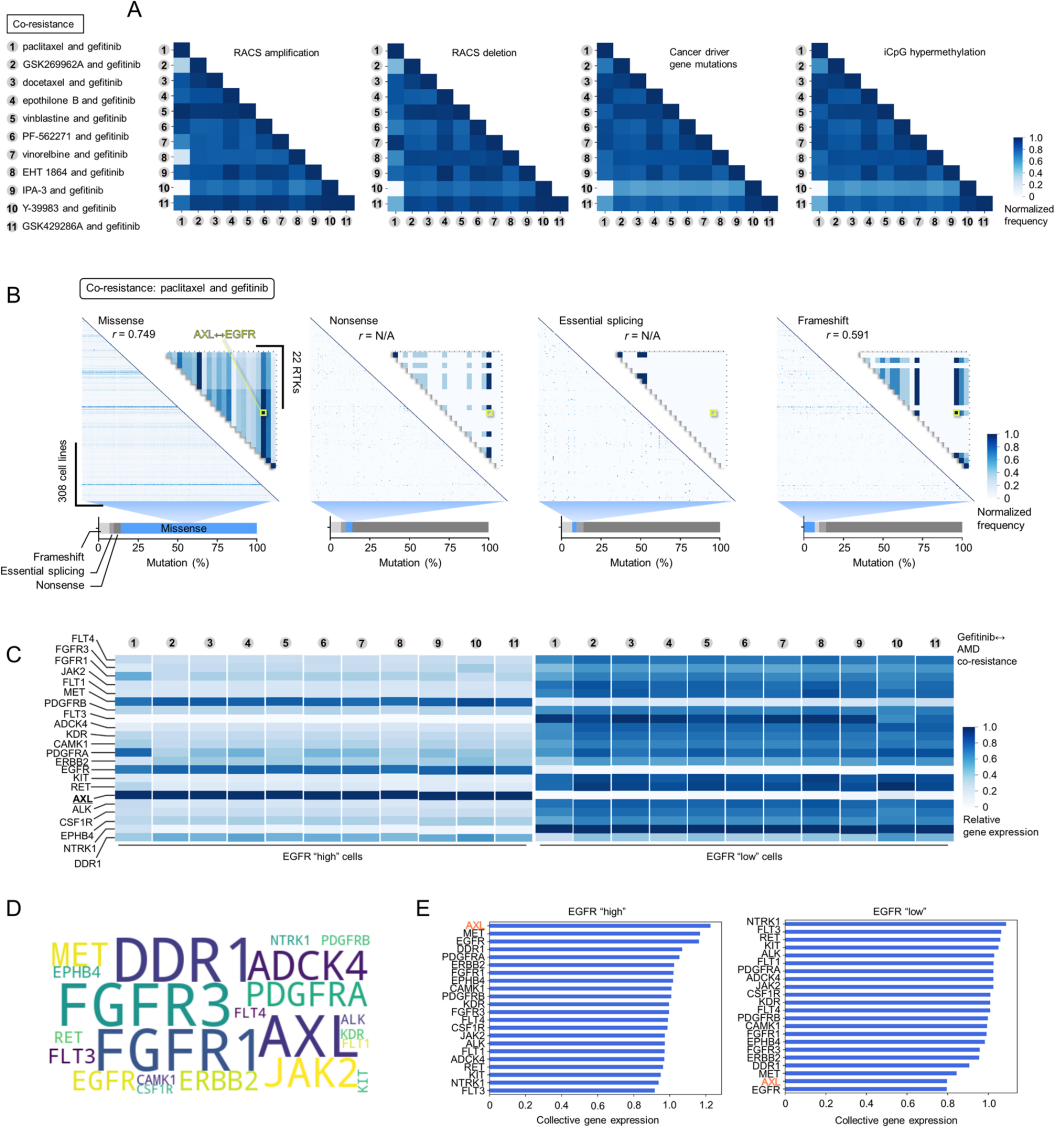
Genomic features associated with co-resistance between an EGFR-TKI and 11 common antimitotic drugs. (**a**) Association matrix of RACS amplification, RACS deletion, CG mutations, or iCpG hypermethylation frequency between cases of indicated co-resistance (11 AMD ↔ EGFR-TKI pairs). Similar denomination of indicated alteration per gene/genomic region in each cell line was normalized. (**b**) Association matrix of missense, nonsense, essential splicing, or frameshift mutation frequency (in all screened genes) between cell lines classified to be co-resistant to paclitaxel and gefitinib. Smaller matrix shows the association of indicated mutation types between 22 RTKs profiled in the GDSC. Similar denomination of indicated mutation per gene in each cell line was normalized. Correlation coefficient values were calculated for AXL and EGFR. (**c**) Relative expression of 22 RTKs per co-resistance case. In each case, cell lines were grouped based on EGFR levels (low or high; the raw intensity value of 4 was set as an expression baseline to categorize cells). Relative values are presented per gefitinib ↔ CTD co-resistance type. (**d**) Word cloud of 22 RTKs based on relative expression in all co-resistance cases. (**e**) Ranking based on the expression of 22 RTKs in all co-resistance cases classified as having low or high EGFR level. Mean gene expression values are presented across all (collective) gefitinib ↔ AMDs co-resistance types. Python (seaborn; https://seaborn.pydata.org/) was used to generate all the plots.

We next attempted to uncover associations between various genomic features and co-resistance cases. We used four different input features processed in the GDSC: (1) cancer driver gene (CG) mutations, (2) amplification or deletion of focal recurrently aberrant copy number segments (RACS), (3) hypermethylated 5′C-phosphate-G-3′ (iCpG) sites, and (4) basal gene expression. Upon inspecting alterations in the first three features, we indicated that many of these co-resistance cases share > 40% frequency of both copy number amplification and deletion (Supplementary Fig. S3a), > 60% frequency of CG mutations, and > 50% of iCpG hypermethylation (Fig. 2a). Comparing these frequencies amongst co-resistance cases indicated that these alterations are mostly correlated but in varying degrees. As an example, co-resistance of paclitaxel ↔ gefitinib and vinblastine ↔ gefitinib share the highest RACS amplification while paclitaxel ↔ gefitinib and vinorelbine ↔ gefitinib share the highest RACS deletion.

Expanding upon the mutational landscape of > 17,000 genes, we mapped four different mutation types: (1) missense, (2) nonsense, (3) essential splicing, and (4) frameshift. We aimed to analyze the extent to which these mutations vary in individual cell lines classified to be co-resistant to the 11 drug pairs and potentially infer informative variations that occur in 22 RTKs. Unanimous to all co-resistance cases, missense mutation is the most prevalent representing > 45% of all mutations while essential splicing represents the least > 2% (Supplementary Fig. S3b). In the paclitaxel ↔ gefitinib co-resistance, where the other 10 co-resistance cases share relatively high frequencies in CG mutations with (Fig. 2a), a fraction of these co-resistant cell lines display high missense mutation frequency while nonsense, essential splicing, and frameshift mutations have sparse signatures (Fig. 2b). Matching the frequencies of these mutations within the 22 RTKs, we found that AXL and EGFR share substantial abundance in missense and frameshift mutations and both are significantly correlated except in nonsense and essential splicing where there is no input data available from the GDSC (Fig. 2b and Supplementary Fig. S3c). While the inferred genomic feature associations can appear obvious (i.e., expected fraction of mutation signatures), we reveal through our analysis that there is relatively limited variation across the 11 co-resistance cases in terms of RACS amplification/deletion, cancer driver gene mutation frequency, iCpG methylation frequency, and mutation type profile. These findings continue to support a common signature profile for co-resistance between gefitinib and CTDs, all these despite the differences in chemistry and biological mode-of-action of CTDs, and generally AMDs.

We next examined the relative basal gene expression of the same set of genes per co-resistance case (Supplementary Fig. S4). Given that the basal gene expression of AXL and EGFR are consistently matched with co-resistance signature between gefitinib and 11 CTDs and that AXL is exceptionally predictive of drug sensitivity response to ErbB family receptor–targeted inhibitors^15^, we hypothesized that AXL should correspond to the level of EGFR in resistant cell lines. To test, we classified and grouped cell lines per co-resistance case according to their level of basal EGFR expression (low and high EGFR-expressing cell lines). Indeed, among all the RTKs evaluated, AXL notably emerged as the highest-ranking RTK that displayed strong correlation with EGFR basal expression (Fig. 2c–e). Although these results are merely associations between basal alterations in cell lines and a co-resistance signature and not a direct measure of drug sensitivity, it is still arguably convincing that AXL and EGFR share a substantial genomic association in the context of co-resistance between gefitinib and a CTD, and arguably, across all gefitinib ↔ CTDs. Therefore, these results extend to the diversifying role of AXL on EGFR signaling which entails subadditive interaction between their targeted inhibitors^16^.

### First-line failure to common antimitotic drugs confers conserved collateral persistence to gefitinib

**Figure 3:**
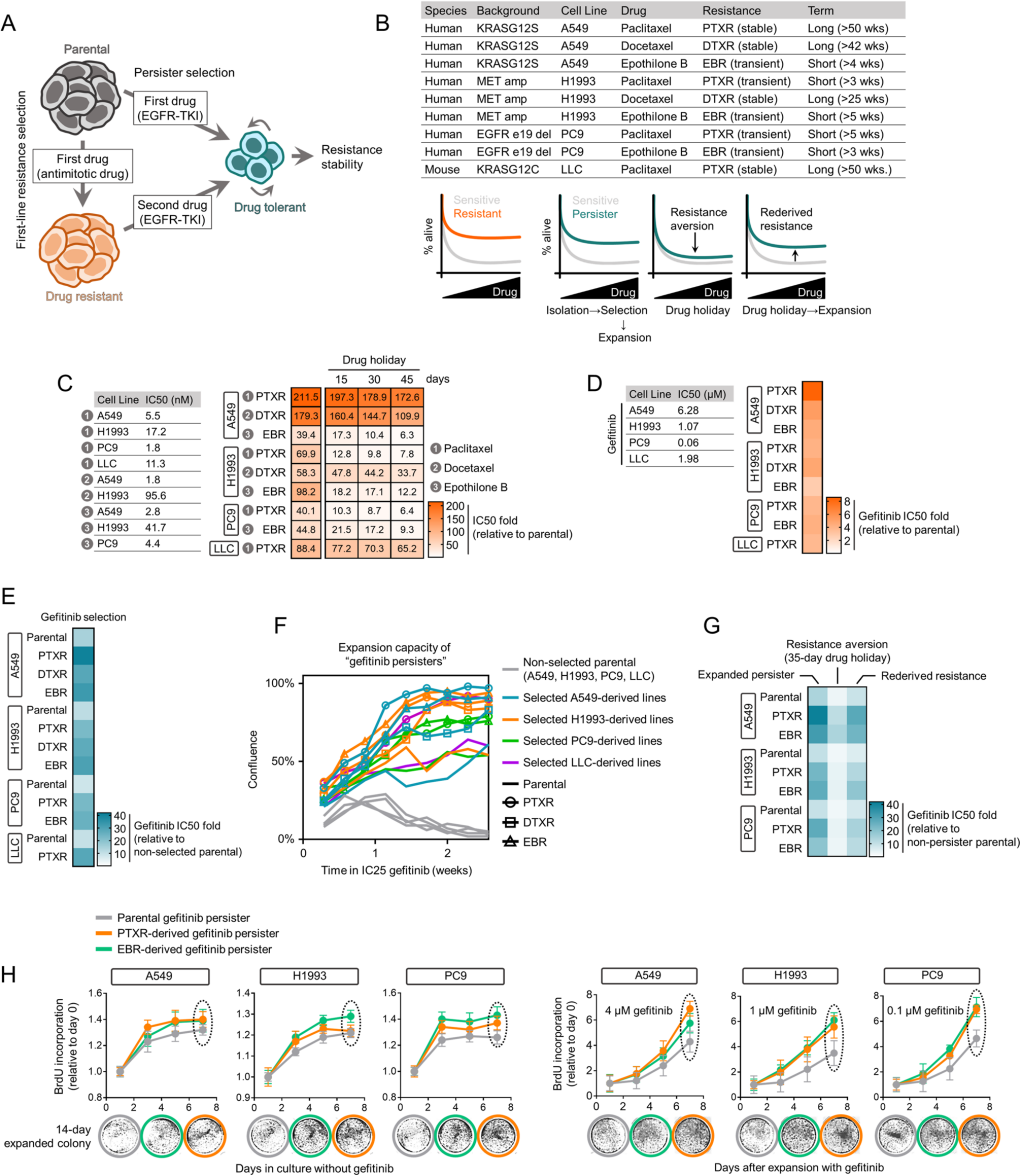
A persister model captures collateral gefitinib resistance stability and trajectory between sensitive and CTD-resistant cells. (**a**) Schematic of drug resistance models. (**b**) Cellular models of primary acquired resistance to paclitaxel, docetaxel, or epothilone B in indicated human and mouse lung cancer cell lines used in this study. See also Methods. (**c**) Characterization of established resistance in cell lines as in b. Stability and transience of resistance were assayed by employing indicated schedules of drug holiday. Cells were treated with or without drugs for 72 h with a concentration dilution series and were assayed for SRB. Resistance was assessed based on drug IC50 values. Representative of three independent experiments. (**d**) Validation of collateral resistance to gefitinib in cell lines as in b. Resistance was assessed based on IC50 fold change relative to parental cells and were assayed as in c. Representative of three independent experiments. (**e**) Resistance characterization of pre-GPs derived from 13 cell lines evaluated after the first high concentration selection. Resistance was assessed based on IC50 fold change relative to non-selected parental cells and were assayed as in c. Representative of three independent experiments. (**f**) Short-term regrowth of indicated pre-GPs in IC25 gefitinib after > 30-day drug holiday following selection as in e. Confluence is quantified in terms of percentage of field of view covered by cells. Representative of four independent experiments. (**g**) Characterization of reversible drug tolerance in A549-, H1993-, and PC9-derived GPs in response to indicated gefitinib-induced selection, expansion, and drug holiday schedules in culture; assayed by SRB (mean ± SD of three biological replicates) (See “Methods”). (**h**) BrdU incorporation assay in indicated GPs. Cells were expanded in culture with (right panel) or without (left panel) gefitinib for indicated time (mean ± SD of three biological replicates). Expanded cells at day 8 were evaluated for colony formation in drug-free media for 14 days. All IC50s were calculated using TableCurve 2D v5.01. GraphPad Prism 7.01 was used to generate all the plots.

To investigate whether the co-resistance signatures mined from the GDSC were experimentally reproducible and reflect a clinically relevant multi-drug treatment failure, we established a sequential model of resistance where the first-line therapy resistance relayed drug-specific trajectory to a secondary resistance. In this regard, we validated the likelihood of collateral resistance to gefitinib in CTD-resistant cancer cells. We contextualized this resistance likelihood in a drug-tolerant persister state (Fig. 3a). This state has been proposed as an alternate route through which cancer cell populations can acquire resistance in response to a variety of strong drug challenges by producing minimal, slow-doubling subpopulations (herein referred to as ‘persisters’)^2,3,13^. We aimed to utilize this model as an experimental tool to distinguish collateral sensitivity or resistance trajectory of CTDs to gefitinib^13,17^.

Modifying previously tested protocols in generating these persisters arising from various targeted therapy pressures^2,13,17^, we developed a gefitinib-tolerant persister model whereby cells acquire the capacity to maintain resistance and expand depending on the presence or absence (via drug holidays) of gefitinib at relatively low concentrations (0.05 ~ 3 μM; see “Methods”). A non-mutational, reversible resistance is a core phenotype observed in these persister cells where removal of gefitinib (> 30 days) allows the derived quiescent surviving persister cells to regrow and re-acquire sensitivity to gefitinib. While poorly understood, this phenotype has also been described in various human cancer cell lines derived from the breast, colon, gastric, lung, and skin following specific drug-induced selection strategies^2,3,13,16,17^.

For our work, we used a panel of nine human and mouse lung cancer cell lines (sensitive or CTD-resistant) with diverse mutational/oncogenic driver profiles (i.e., KRAS G12 mutants, MET amplification, EGFR exon 19 deletion, among others) (Fig. 3b). The CTD sensitivity profile of our parental and CTD-resistant models revealed that all parental cells are hypersensitive (IC50 < 100 nM measured after 72 h) to paclitaxel, docetaxel, and epothilone B while all CTD-resistant lines (PTXR, DTXR, and EBR) are markedly resistant to these drugs and this CTD resistance is either stable or transient (loss of resistance after 15- to 45-day drug holiday) (Fig. 3c). At the parental state, gefitinib sensitivity of A549, H1993, PC9, and LLC cells are in the range of 0.06 to 6.28 μM. This intermediate sensitivity profile is important to address, in some degree, potential off-target mechanisms later when CTD-resistant cells derived from these parental lines are tested for gefitinib activity. Indeed, all CTD-resistant cells displayed varied but considerably high collateral resistance to gefitinib compared to those of the sensitive cells (Fig. 3d and Supplementary Fig. S5). While we cannot rule out other potential off-target mechanisms that might influence this gefitinib collateral resistance, it appears that this signature can be observed in CTD-resistant cells derived from parental cells with varying sensitivities to gefitinib.

Prior to the generation of gefitinib persisters (GPs), we first subjected these cells to strong gefitinib selection followed by a long-term culture in drug-free media (pre-GPs). While all pre-GPs displayed increased resistance to gefitinib, those derived from CTD-resistant cells showed higher gefitinib resistance (Fig. 3e). One possible explanation to account for this observation is differences in cell culture confluence. These pre-GPs are exposed to a gefitinib concentration lower than the drug’s IC50 values per derived pre-GP. During a 2-week expansion, parental cells that were not selected for gefitinib displayed negligible proliferation while those selected remained considerably resistant (colonies formed after 1 week). Pre-GPs derived from CTD-resistant cells displayed higher confluence and are more resistant to varying degrees (> 50%) than pre-GPs derived from parental (mostly < 50%) (Fig. 3f).

Characterization of GPs derived from these pre-GPs revealed that they are in a reversible state of drug resistance (Fig. 3g). As previously observed in a variety of cancer models^1,3,13,17^, a functional signature of this EGFR-TKI persisters (i.e., erlotinib) is the capacity to revert back to sensitivity after an extended drug holiday (previous experimental observations vary from 20 to 40 passages). In our model, 12 ~ 14 passages during a > 30-day drug holiday were sufficient to observe resistance aversion across all GP lines. However, we should note that this aversion was still not adequate to reach parental-like sensitivity.

A delayed DNA synthesis accompanies a signature of negligible growth of these persisters^3,13,18,19^. We used BrdU, a thymidine analogue, to examine DNA synthesis in both pre-GPs and GPs. Before expansion, all derived pre-GPs experienced a widespread growth arrest (Supplementary Fig. S6). Upon expansion (~ 12 days) in low gefitinib concentration (approx. IC10) for a week, there was an apparent re-growth from the third day after plating. To our surprise, all pre-GPs, derived from parental or CTD-resistant cells, displayed similar signatures of DNA synthesis. Next, we uncoupled growth recovery states of GPs upon drug holiday and re-derivation of persistence in derived GPs. During drug-free culture, all GPs displayed negligible growth, but those derived from CTD-resistant cells retained colony-forming potential after a long-term culture (Fig. 3h). Following this drug holiday, re-deriving GPs through exposure to gefitinib recovered growth arrest phenotype, with those derived from CTD-resistant cells gaining a sharp increase in proliferative capacity compared to parental-derived GPs. To further expand our collateral resistance analysis to other EGFR-TKIs that share chemical similarity with gefitinib, we characterized the response of GPs derived from parental or CTD-resistant cells to erlotinib and afatinib. A compelling collateral resistance to both of these EGFR-TKIs coincides with the persister phenotype of GPs derived from CTD-resistant cells but not from parental cells (Supplementary Fig. S7), suggesting that CTD resistance can confer a much more widespread tolerance to EGFR-TKIs.

### AXL expression and stability in drug persisters dictate secondary resistance and are associated with relapse

**Figure 4:**
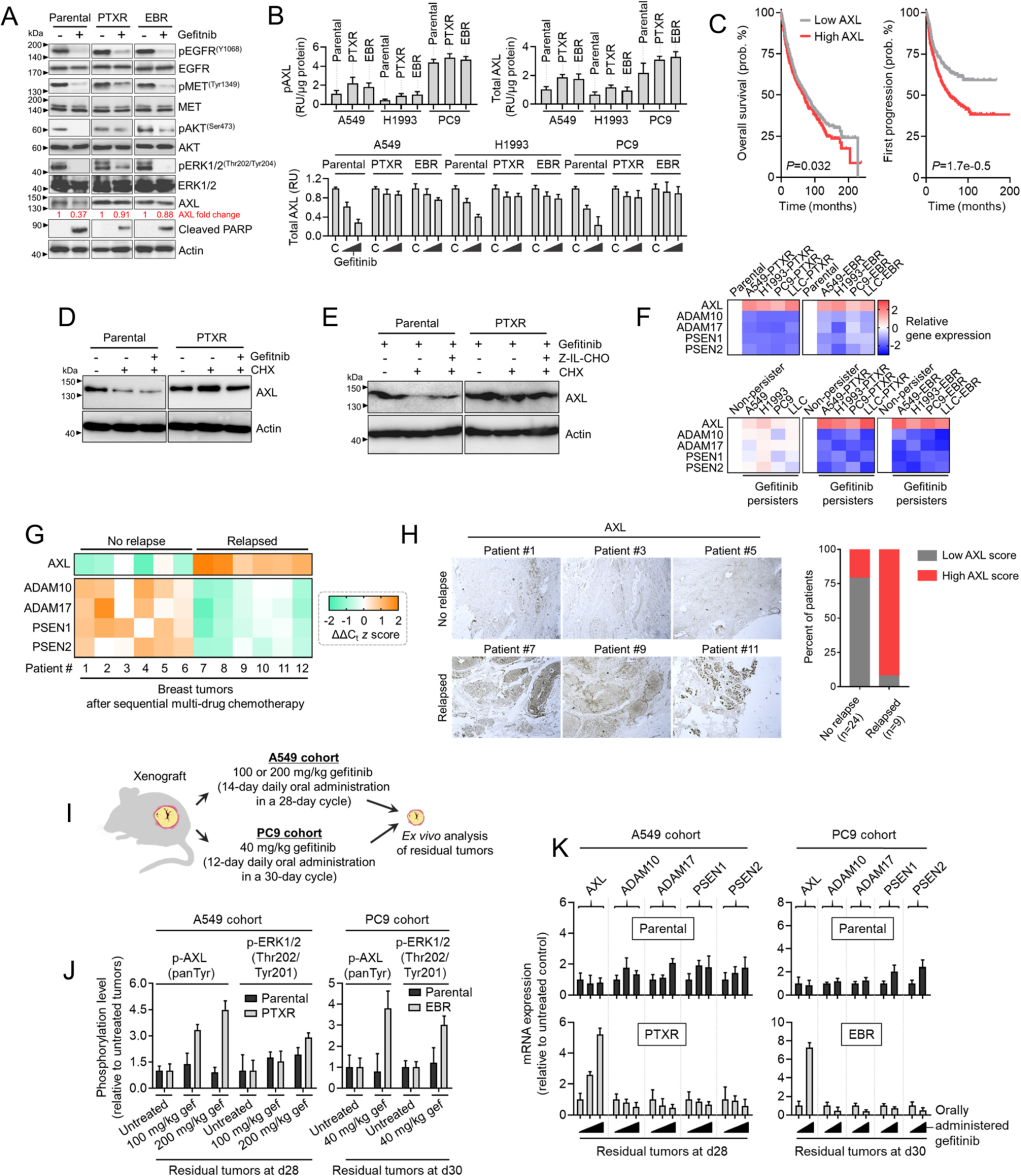
AXL expression and stability are markers of a secondary resistance. (**a**) Western blot analysis of indicated proteins in A549-parental, -PTXR, and -EBR cells upon treatment with or without 6 μM gefitinib for 24 h. Representative of two independent experiments. 45 μg of total cell lysates were loaded per lane. Samples from the same cell line were run on the same gel. Paired samples are highlighted in black frame. (**b**) Phosphorylated AXL (pAXL) and total AXL quantified in parental, PTXR, and EBR lines derived from A549, H1993, or PC9 cells upon treatment with or without 4 or 8 μM gefitinib for 24 h (mean ± SD of three biological replicates). (**c**) Kaplan–Meier plots (KMPlotter; https://kmplot.com/analysis/) of overall (patients n = 1926) and first progression (FP; patients n = 982) survival of lung cancer patients. Patient survival data were stratified by AXL expression (low or high) in their primary tumors. *P* values were calculated using a log rank test. (**d**) Western blot analysis of AXL in parental and PTXR cells derived from A549 upon treatment with or without 5 μM gefitinib for 24 h followed by treatment with 25 μg/mL CHX for 8 h. Actin was used as a loading control. Representative of two independent experiments. 35 μg of total cell lysates were loaded per lane. Samples from the same cell line were run on the same gel highlighted in black frame. (**e**) Western blot analysis of AXL in parental and PTXR cells derived from A549 upon treatment with 5 μM gefitinib and with or without 800 nM Z-IL-CHO for 24 h followed by treatment with or without 25 μg/mL CHX for 8 h. Actin was used as a loading control. Representative of two independent experiments. 40 μg of total cell lysates were loaded per lane. Samples from the same cell line were run on the same gel highlighted in black frame. (**f**) qRT-PCR analysis of AXL and PS-RIP marker expression in indicated parental, CTD-resistant cell lines, and GPs. Values are relative to parental and were normalized to GAPDH levels (mean ± SD of three biological replicates). (**g**) qRT-PCR analysis of AXL and PS-RIP marker expression in FFPE tumor tissue sections from breast cancer patients who underwent sequential multi-drug chemotherapy. Log-transformed gene expression values are relative to the sample with the lowest AXL expression and were normalized to GAPDH levels (mean ± SD of three biological replicates). (**h**) Immunohistochemical analysis of indicated FFPE tumor tissue sections used in e. Sections were blocked and probed with AXL antibody and detected using a DAB chromagen kit. All sections were photographed with an inverted phase contrast microscope (original magnification, 200 ×). Scale bar, 100 μm. Representative of two independent experiments (left panel). Scored IHC expression of AXL in tumor sections of relapsed or non-relapsed breast cancer patients (right panel). (**i**) Schematic of xenograft model and gefitinib therapy. (**j**) ELISA sandwich-based measurement of pan tyrosine phosphorylation of AXL and threonine 202 / tyrosine 201 phosphorylation of ERK1/2 in xenograft tumors derived from parental and PTXR cells excised at day 28 or 30 detailed in i (mean ± SD of four biological replicates). (**k**) qRT-PCR analysis of AXL and PS-RIP marker expression in the same tumor samples as in i. Values are relative to parental untreated and were normalized to GAPDH levels (mean ± SD of four biological replicates). GraphPad Prism 7.01 was used to generate all the plots.

We next asked whether key components in EGFR signaling are maintained or regulated upon gefitinib treatment in both parental and CTD-resistant cells. Upon treatment, gefitinib effectively reduced phosphorylation of EGFR, MET, AKT, and ERK in both parental and CTD-resistant cells (both PTXR and EBR), although in varying efficiency (Fig. 4a). This led us to question whether there exists a bypass signal acting to compensate for these functional losses in EGFR signaling, at least for CTD-resistant cells that exhibit collateral EGFR-TKI resistance. Several compensatory resistance routes upon EGFR blockade have been uncovered including the activation of AXL, its ligand (GAS6)-independent activity transactivated by EGFR^16^, and its reduced proteolytic shedding^16^. We found that AXL expression and receptor abundance were maintained upon gefitinib treatment in CTD-resistant cells while they were inhibited in parental cells (Fig. 4a,b). Note that higher basal AXL phosphorylation and receptor abundance in CTD-resistant cells than parental cells (Fig. 4b).

To broadly substantiate AXL expression with drug response to EGFR-TKIs, we examined the relationship of drug IC50 values with AXL expression in silico through an open-access application that mined the GDSC and Cancer Cell Line Encyclopedia (CCLE) data sets^20^. We found substantial correlation between high AXL expression and drug resistance to EGFR-TKIs gefitinib, erlotinib, afatinib, lapatinib, and cetuximab in a variety of malignancies (Supplementary Fig. S8a). In a lung cancer patient cohort, Kaplan–Meier analysis of microarray data supported this association with high AXL expression significantly correlated with poor first progression survival of patients who underwent chemotherapy, while AXL expression did not adequately correlate with a signature of overall survival (Fig. 4c). Interestingly, in pan-cancer cohorts, high AXL is associated with poor RFS in patient samples with enriched mesenchymal stem cells (Supplementary Fig. S8b).

We next considered the possibility that the maintained AXL expression and receptor abundance in CTD-resistant cells upon gefitinib-dependent blockade of EGFR signaling can be the result of a slow turnover. First, we examined the stability of AXL upon treatment with a protein synthesis inhibitor cycloheximide (CHX) for 8 h. Accordingly, AXL degradation was suppressed in PTXR cells while degradation was effectively induced in their respective parental cells (Fig. 4d). It should be noted that CHX still reduced the protein level of AXL in PTXR cells when co-treated with gefitinib. We wondered if γ-secretase-mediated AXL cleavage is directly involved in this process. Upon treatment with γ-secretase inhibitor Z-Ile-Leu-aldehyde (Z-IL-CHO), the immediate CHX-dependent AXL degradation in parental cells was moderately rescued while that in PTXR cells was only marginally affected (Fig. 4e). This was further supported by the twofold increase in AXL receptor abundance in parental cells upon Z-IL-CHO treatment while the effect on CTD-resistant PTXR and EBR cells remained insignificant (Supplementary Fig. S9). These findings hint at the differential basal profiles of presenilin-dependent regulated intramembrane proteolysis (PS-RIP) components in parental and CTD-resistant cells. In light of this, we characterized the gene expression of markers for PS-RIP-directed degradation of AXL. Notably, key biomarkers of PS-RIP (ADAM10, ADAM17, PSEN1, and PSEN2) were downregulated in all CTD-resistant cells along with an amplified AXL (Fig. 4f). These PS-RIP markers showed variable expressions in parental-derived GPs while consistently downregulated in GPs derived from CTD-resistant cells.

In a lung cancer patient cohort, both high expression of ADAM17 and PSEN2 were correlated with better first progression survival of patients who underwent systemic therapy while in a breast cancer patient cohort, ADAM17 and PSEN2 expression led to a much narrower gap in relapse-free survival (RFS) of patients who underwent systemic therapy compared to the significantly wide gap in overall survival of patients under the systemically untreated cohort (Supplementary Fig. S10a,b). Corroborating these results using clinically-relevant samples, we further analyzed tumors from breast cancer patients who underwent sequential cycles of muti-drug chemotherapy. We assumed that the patient tumors that re-appeared as a result of relapse contain drug-refractory tumor sub-populations that evolved through cycles of therapeutic pressure, pointing to the previously reported tracking of clonal resistance evolution in breast cancer patients following sequential cycles of chemotherapy^13,21^. We found that there is a marked AXL expression in tumors of patients that relapsed following therapy as evidenced by gene and protein expression analyses (Fig. 4g,h). In line with this, downregulation of PS-RIP genes were observed in these relapsed tumors (Fig. 4g). Intriguingly, two AXL-negative relapsed breast tumors displayed minimal upregulation of PS-RIP gene expression when compared to a non-relapsed sample that has a low basal AXL expression (Supplementary Fig. S10c). Although a larger sample size is needed to draw relevant conclusions, this might indicate that the PS-RIP is perturbed and is not able to properly regulate AXL homeostasis in relapsed, residual tumors.

To dissect whether these associations are supported in gefitinib-resistant tumors, we subjected mouse xenografts derived from our A549 and PC9 models to gefitinib therapy (Fig. 4i). Residual tumors following this therapy were considered as ‘gefitinib-resistant’. Xenografts from CTD-resistant cells displayed pronounced collateral resistance to gefitinib even in varying doses (both during and at the end of the gefitinib therapy) while those derived from parental cells were unanimously inhibited (Supplementary Fig. S11a,b). CTD-resistant cell-derived residual tumors displayed pronounced pan tyrosine phosphorylation of AXL compared to parental-derived tumors where this phosphorylation remained unaffected even after a high dose of gefitinib therapy (Fig. 4j). Furthermore, CTD-resistant cell-derived gefitinib residual tumors displayed the same downregulated PS-RIP signature along with a pronounced AXL activation (Fig. 4k). These results demonstrate the predisposition of AXL degradation via decreased ectodomain shedding capacity and γ-secretase activity in CTD-resistant cell-derived gefitinib residual tumors and collaterally resistant cells.

Next, we wondered whether this EGFR-TKI collateral resistance specific in CTD-resistant cells reflects independence from an autocrine loop involving GAS6 (AXL ligand). Across our panel of PTXR and EBR cells and their parental lines, we characterized GAS6 expression. All CTD-resistant cells did not show significant changes in GAS6 expression compared to parental cells (Supplementary Fig. S12a). However, at the GP state, all of the CTD-resistant cells significantly upregulated GAS6 which is further supported by a sharp increase in expression in gefitinib-induced residual tumors derived from CTD-resistant cells (Supplementary Fig. S12a,b). While we cannot preclude other interpretations, these data suggest that prior to a selection pressure by EGFR-TKIs, at the basal state, AXL regulation associated with collateral EGFR-TKI resistance in CTD-resistant cells is independent of the GAS6 ligand-receptor signaling activation but shifts to a GAS6-AXL autocrine manner upon entering a drug-tolerant persister state as a result of EGFR-TKI pressure. Taken together, these results suggest that proteolytic processing of AXL is impeded in CTD-resistant cells that result in delayed AXL turnover and maintained translation promoting collateral EGFR-TKI resistance. It is subject to further study whether GAS6-dependent autocrine mechanism is functionally important in this context.

### AXL expression and stability are regulated in metastasis and promote stemness of gefitinib persisters derived from chemotherapy-resistant cells

AXL is required at multiple steps of the metastatic cascade^22^ and is directly linked with resistance to various therapies^23^. There is no significant difference between low and high AXL expression in the distant metastasis-free survival (DMFS) of breast cancer patients under the systemically untreated cohort. However, high AXL expression significantly correlated with poor DMFS of patients who underwent systemic therapy (Supplementary Fig. S13a). Expanding these associations, high AXL expression is correlated with breast metastasis to the lymph node, brain, liver, and lung, among other sites (Supplementary Fig. S13b). In our small cohort of relapsed breast cancers, high IHC positivity to AXL significantly correlated with metastasis to the lymph node (Supplementary Fig. S13c).

To ask whether metastatic cancer relapse associated with therapy is linked with decreased PS-RIP, we further inspected both non-relapsed and relapsed breast tumor tissues that stained positive to high AXL and correlated their expression with patient’s HER2 status. Along with an amplified AXL, PS-RIP markers were downregulated in relapsed tumors while these markers were variably expressed in high levels in non-relapsed tumors (Supplementary Fig. S13d). Intriguingly, HER2 positivity together with AXL amplification in these relapsed tumors express high levels of basal EGFR (Supplementary Fig. S13e). Because co-signaling of HER2 and AXL can mediate the metastasis of HER2 + breast cancer^22^, it will be interesting to dissect the requirement of AXL as a bypass signal during therapy relapse in this context.

Curious whether AXL can differentiate between the metastasis of parental and CTD-resistant cells, we generated a murine model of lung-specific metastasis using LLC-derived cells (Supplementary Fig. S13f). Remarkably, LLC-PTXR-derived tissues from induced lung metastasis (at day 15 after inoculation) showed larger nodule sizes indicating more efficient seeding than those derived from parental cells (Supplementary Fig. S13g). We observed an elevated AXL protein expression in PTXR-derived tissues from primary metastatic lung nodules and tumor xenografts compared to parental-derived tumor tissues but AXL gene expression is only markedly increased in primary lung metastasis and metastasized lung tumors in liver and spleen (Supplementary Fig. S13h,i). Taken together, these results suggest that AXL is less prone to ligand-independent receptor shedding during therapy relapse and metastasis.

**Figure 5:**
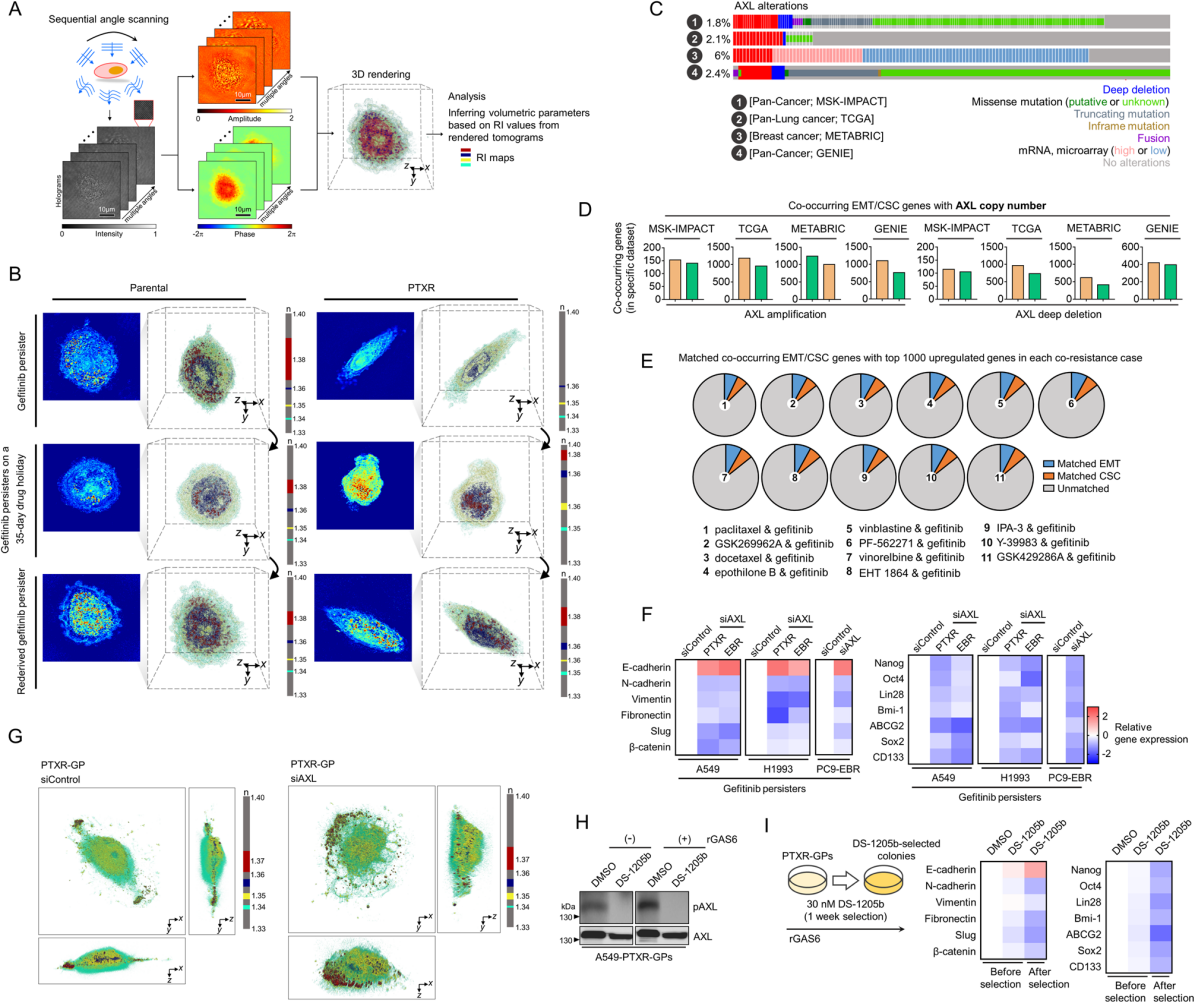
AXL regulates an adaptive state of resistance via EMT and CSC programs. (**a**) Three-dimensional (3D) refractive index (RI) map of cells. (**b**) 3D RI distributions of indicated live A549-derived GPs plated on a glass-bottom imaging dish. Representative images show snapshots from live holotomography imaging. The color legend indicates RI value. TomoStudio (http://www.tomocube.com/product/tomostudio/) to generate the images. (**c**) OncoPrints visualizing AXL alterations across the indicated data sets. Number of patients with indicated AXL alterations in each data set are: MSK-IMPACT [181/10,336 patients], TCGA [24/1144], METABRIC [114/1904], and GENIE [1255/52292]. Datasets were accessed via cBioPortal (https://www.cbioportal.org/). GraphPad Prism 7.01 was used to generate the plot. (**d**) Co-occurrence analysis of indicated AXL copy number or mRNA alterations with EMT (out of 342 genes) and CSC (out of 1782 genes) associated genes, respectively, per indicated data set as in c. GraphPad Prism 7.01 was used to generate the plot. (**e**) Proportion of co-occurring EMT/CSC genes with AXL matched with the top 1000 upregulated genes in each co-resistance case. List of these top hit genes are derived from Supplementary Fig. S4. Python (seaborn; https://seaborn.pydata.org/) was used to generate the plot. Gene annotations were accessed via dbEMT (http://dbemt.bioinfo-minzhao.org/) and CSCdb (http://bioinformatics.ustc.edu.cn/cscdb/). (**f**) qRT-PCR analysis of expression of EMT and CSC markers in indicated GPs upon AXL RNAi. Values are relative to parental untreated and were normalized to GAPDH levels (mean ± SD of two biological replicates). GraphPad Prism 7.01 was used to generate the plot. (**g**) 3D RI tomograms of indicated fixed GPs upon AXL RNAi followed by gefitinib treatment. TomoStudio to generate the images. (**h**) Western blot analysis of phospho-AXL in A549-PTXR-GPs upon treatment with or without 80 nM DS-1205b for 24 h. Prior to treatment, cells were stimulated with or without 250 ng/mL recombinant GAS6. Total AXL was used as a loading control. Representative of two independent experiments. 42 μg of total cell lysates were loaded per lane. Samples from the same cell line were run on the same gel highlighted in black frame. (**i**) qRT-PCR analysis (right panel) of expression of EMT and CSC markers in indicated GPs upon DS-1205b selection as schematized (left panel). Values are relative to parental untreated and were normalized to GAPDH levels (mean ± SD of two biological replicates; similar color scale as in (**f**). GraphPad Prism 7.01 was used to generate the plot.

Development of secondary resistance following initial response to targeted therapies have been correlated with metastatic potential and the epithelial-to-mesenchymal transition (EMT) in several cancers^24–28^. EMT pathways mediate the generation of drug-tolerant persisters^28^. We first characterized cell morphologies using holotomography (HT)^29–31^, a 3D quantitative phase imaging technique for label-free and quantitative bioimaging. HT, also known as optical diffraction tomography, is an optical analogy to X-ray computed tomography. From the measurements of multiple 2D optical fields of a sample, HT reconstructs the 3D refractive index (RI) distributions of the sample, providing 3D high-resolution morphological information of individual cells (Fig. 5a and Movie S1 and S2). 3D RI distributions allowed us to assess the organization of cellular organelles throughout the process of deriving gefitinib persistence from parental and PTXR cells. RI tomograms evidently demonstrated rod/spindle-like projections along with apparent changes in orientations of cellular compartments at the single-cell level of PTXR-derived GPs, which are morphological features of the mesenchymal phenotype, whereby GPs derived from parental cells maintain a consistent morphological profile remaining mostly epithelial-like (Fig. 5b). These RI tomograms also revealed that certain organelles (i.e., vesicle-like bodies) in PTXR-derived GPs seem to be regulated as persistence is maintained or averted (during drug holiday).

We next aimed to correlate EMT and stemness gene expression with AXL. In four large data sets of cancer patient cohorts (MSK-IMPACT, TCGA, METABRIC, and GENIE), alterations in AXL are observed (Fig. 5c). Interestingly, the number of co-occurring genes regulating the EMT and cancer stem cell (CSC) programs correspond to the type of AXL alterations (i.e., high in AXL amplification, low in AXL deletion) at the copy number level (Fig. 5d). However, these associations are modest at the mRNA level (Supplementary Fig. S14a). To connect this expression co-occurrence to co-resistance between CTDs and gefitinib, we matched the co-occurring EMT and CSC genes with 1000 highly upregulated genes in each co-resistance case. Intriguingly, the number of matched EMT and CSC genes are analogous across the 11 co-resistance cases (Fig. 5e). CTD-resistant cell-derived residual tumors following high dose gefitinib therapy displayed pronounced activation of EMT and CSC gene expressions (Supplementary Fig. S14b). Next, we directly linked EMT and CSC programs to AXL regulation in GPs. AXL RNAi led to the reversal of the mesenchymal phenotype and gene expression in GPs derived from CTD-resistant cells (Supplementary Fig. S14c; Fig. 5g,f). This reversion is accompanied by morphological transition as revealed by HT (Fig. 5h). To support these RNAi data, we used the small-molecule AXL selective inhibitor DS-1205b (Fig. 5h). DS-1205b induced a strong reversal of the EMT and stemness phenotype in PTXR-derived GPs upon selection for 1 week while marginally affecting the phenotype upon short-term treatment (Fig. 5i). Overall, these findings indicate that the mesenchymal state and stem cell-like properties of GPs derived from CTD-resistant cells are tightly linked with AXL status.

### AXL is required and sufficient as a bypass signal to compensate for EGFR inhibition and supports secondary gefitinib resistance

**Figure 6:**
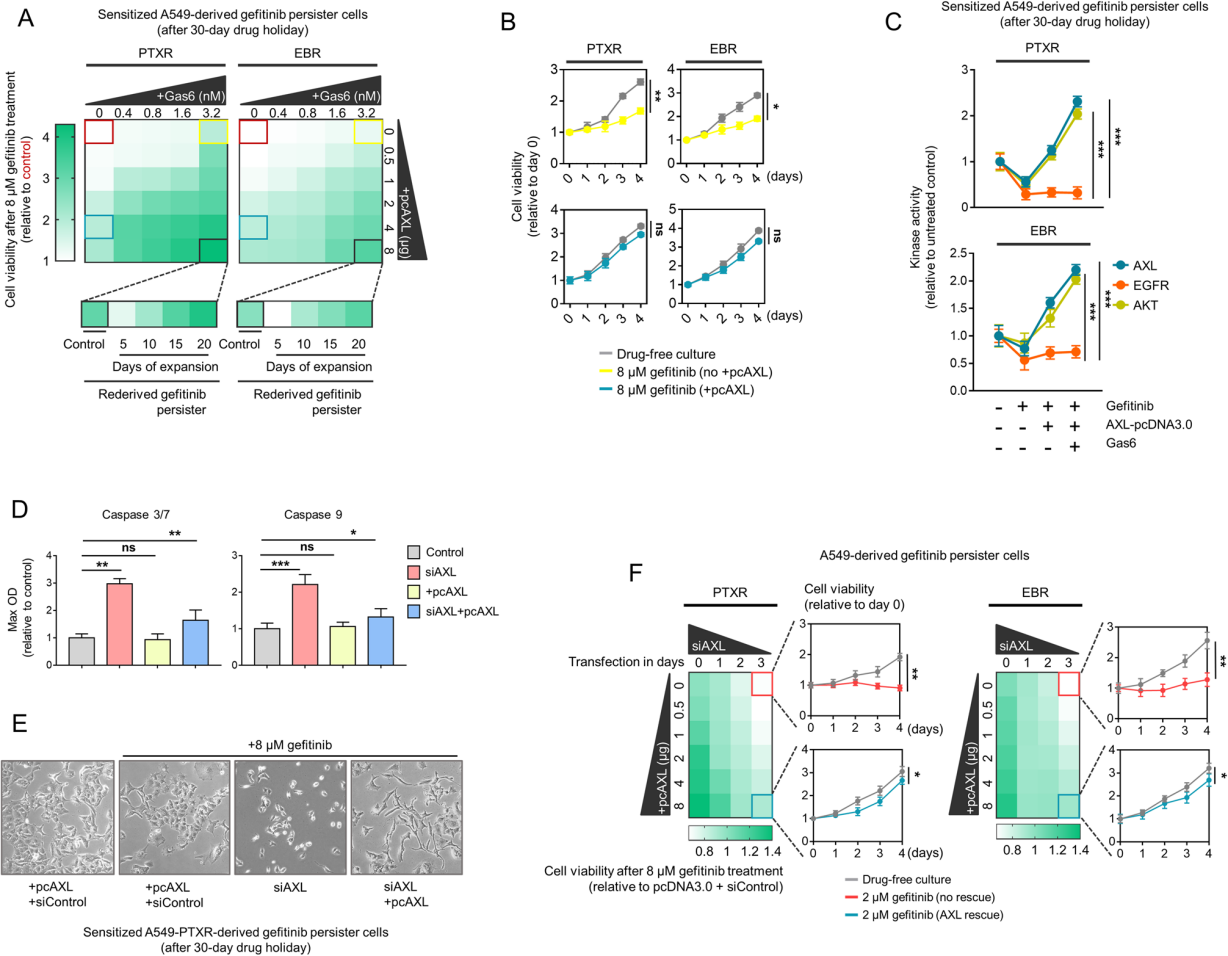
AXL is a bypass node that supports a route to a secondary resistance. (**a**) Relative cell viability of A549-PTXR and -EBR-derived GPs sensitized after a 30-day drug holiday treated with 8 μM gefitinib for 24 h. Cells were treated or transfected with or without the indicated concentrations of Gas6 ligand or pcDNA3.0-AXL plasmid for 24 h. Indicated rederived GPs were subjected to further expansion in gefitinib. Representative of three independent experiments. Python (seaborn) was used to generate the plot. (**b**) Proliferation of indicated sensitized GPs upon treatment with 3.2 nM Gas6 ligand (yellow) or transfection with 4 μg pcDNA3.0-AXL plasmid (blue) for 24 h followed by treatment with 8 μM gefitinib for 24 h. *P < 0.05, **P < 0.01, ***P < 0.005, Student’s t test. GraphPad Prism 7.01 was used to generate the plot. (**c**) Luminescence-based kinase assay for AKT, AXL, and EGFR in indicated cells as in a. Cells were treated with or without 8 μM gefitinib for 24. Prior to this, cells treated or transfected with or without 1.6 nM Gas6 ligand or 4 μg pcDNA3.0-AXL plasmid for 24 h. Values are relative to untreated control (mean ± SD of two biological replicates; *P < 0.05, **P < 0.01, ***P < 0.005, Student’s t test). GraphPad Prism 7.01 was used to generate the plot. (**d**) Caspase 3/7 DEVDase and caspase 9 activities of A549-PTXR-derived GPs sensitized after a 30-day drug holiday treated with 8 μM gefitinib for 24 h. Prior to drug treatment, cells were subjected to AXL RNAi and/or transfection with or without pcDNA3.0-AXL plasmid for 24 h. Values are relative to transfection control (mean ± SD of three biological replicates; *P < 0.05, **P < 0.01, ***P < 0.005, Student’s t test). GraphPad Prism 7.01 was used to generate the plot. (**e**) Phase-contrast images of cells as in c. Scale bar indicates 100 μm. (**f**) Cell viability of indicated A549-derived GPs upon AXL RNAi and/or transfection with or without pcDNA3.0-AXL plasmid for 24 h. Indicated cells under rescue or no rescue conditions were further treated with or without 2 μM gefitinib. Values are relative to plasmid and RNAi control transfection. Representative of two independent experiments. *P < 0.05, **P < 0.01, ***P < 0.005, Student’s t test. Python (seaborn; https://seaborn.pydata.org/) was used to generate the plot.

To test whether the bona fide gene expression and receptor activation of AXL have consequences on secondary gefitinib resistance in CTD-resistant cells, we first characterized the activity of DS-1205b. As expected, both CTD-resistant PTXR and EBR cells displayed increased sensitivity to DS-1205b than parental cells, owing to their amplified AXL (Supplementary Fig. S15a). Following this, we subjected the cells to ectopic treatment with GAS6 ligand or transfection with AXL-pcDNA3.0 construct. We found that collateral gefitinib resistance in CTD-resistant cells can be triggered in a ligand-dependent or -independent AXL activation manner (Supplementary Fig. S15b). We next sought to assess how AXL abundance influences the stability of collateral gefitinib resistance. Employing sensitized GPs that underwent long-term drug holiday, we uncovered that ligand-dependent receptor activation led to an adequate population rescue that was able to expand and re-derive gefitinib persistence when challenged with growth inhibitory concentrations of gefitinib (Fig. 6a,b). In addition, EGFR activity in GPs derived from CTD-resistant cells was consistently inhibited upon gefitinib treatment but was unaffected by ligand-dependent or -independent AXL receptor activation, even at high concentrations of GAS6 or AXL-pcDNA. In contrast, AXL receptor activation was sufficient to rescue both AXL and AKT kinase activities from gefitinib-induced blockade (Fig. 6c). These results further confirm that transactivation of AXL upon gefitinib-induced EGFR blockade is sufficient in promoting collateral gefitinib resistance.

Given these, we hypothesized that AXL is required to reprogram apoptosis in this context. Indeed, AXL silencing led to increased activities of caspases 3/7 and 9 and an apparent induction of apoptotic morphology in sensitized GPs derived from CTD-resistant cells (Fig. 6d,e). Consistent with the rescue observation, AXL receptor activation rescued these apoptotic phenotypes. Moreover, cells with scant AXL expression displayed retarded resistance capacity while cells with rescued AXL expression recovered and exhibited resistance potential (Fig. 6f). Altogether, our data support a functional compensatory role for AXL upon EGFR blockade and promotes a gefitinib-specific secondary resistance following failure to CTDs.

### A candidate EGFR inhibitor co-targets AXL by promoting its degradation and is synergistic with gefitinib in suppressing sequential resistance

**Figure 7:**
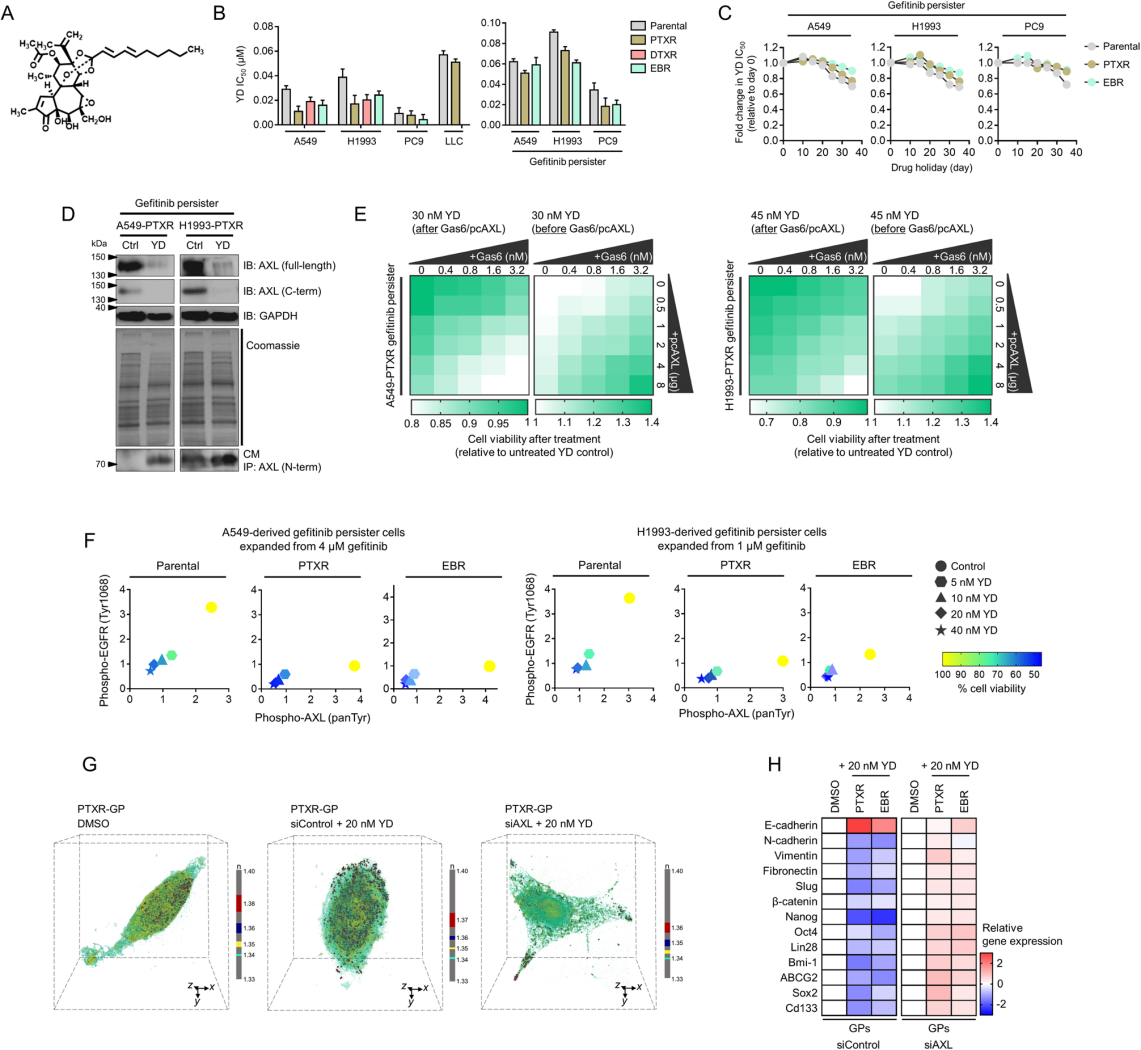
A candidate EGFR-TKI blocks AXL-mediated bypass route to a stable secondary gefitinib resistance in CTD-resistant cells. (**a**) 2D structure of the diterpenoid yuanhuadine (YD). (**b**) IC50 values of YD in indicated cell lines and GPs. Cells were treated with or without YD for 72 h with a concentration dilution series and were assayed for SRB. Values represent mean ± SD of three biological replicates. GraphPad Prism 7.01 was used to generate the plot. (**c**) Characterization of indicated sensitized GPs upon treatment with YD. IC50 values are relative to day 0. Representative of two independent experiments. GraphPad Prism 7.01 was used to generate the plot. (**d**) Characterization of YD-induced AXL suppression in indicated PTXR-derived GPs. Cells were treated with or without 30 nM YD (in A549-derived GPs) and 45 nM YD (in H1993-derived GPs) for 48 h. Conditioned culture media (CM) were harvested, immunoprecipitated with antibody against N-terminal AXL, and immunoblotted using anti-N-terminal AXL. Cells were also subjected for immunoblotting with anti-C-terminal AXL. GAPDH was used as a loading control. In-gel proteins were visualized with Coomassie blue. Representative of two independent experiments. 40 μg of total cell lysates were loaded per lane for immunoblotting while 50 μg of pulled-down proteins were loaded for immunoprecipitation. Samples from the same cell line were run on the same gel. Paired samples are highlighted in black frame. (**e**) Cell viability of indicated PTXR-derived GPs upon treatment with 30 nM YD (in A549-derived GPs) and 45 nM YD (in H1993-derived GPs) for 48 h. Prior to YD treatment, cells were treated or transfected with or without the indicated concentrations of Gas6 ligand or pcDNA3.0-AXL plasmid for 24 h. Values are relative to untreated control. Representative of three independent experiments. Python (seaborn; https://seaborn.pydata.org/) was used to generate the plot. (**f**) ELISA sandwich-based measurement of EGFR tyrosine 1068 phosphorylation and AXL pan tyrosine phosphorylation and concurrent cell viability analysis of indicated GPs. Cells were treated with or without increasing concentrations of YD (5, 10, 20, and 40 nM) for 48 h. Representative of two independent experiments. Python (seaborn; https://seaborn.pydata.org/) was used to generate the plot. (**g**) 3D RI distributions of indicated fixed A549-derived GPs upon AXL RNAi followed by treatment with or without 20 nM of YD for 48 h. Representative images show snapshots from holotomography imaging. The color legend indicates RI value. TomoStudio to generate the images. (**h**) qRT-PCR analysis of expression of EMT and CSC markers in indicated GPs with the same conditions as in g. Values are relative to DMSO control and were normalized to GAPDH levels (mean ± SD of two biological replicates). Python (seaborn; https://seaborn.pydata.org/) was used to generate the plot. All IC50s were calculated using TableCurve 2D v5.01.

To chemically probe the function of AXL in the context of collateral gefitinib resistance, we used YD (Fig. 7a), an antitumor agent we previously characterized to have concurrent AXL-dependent inhibitory mode-of-action in addition to having EGFR-targeting activity in gefitinib-resistant lung cancer models^32,33^. YD displayed a more potent growth killing capacity in cancer cell lines with acquired resistance to paclitaxel, docetaxel, and epothilone B, than those of their parental origin (Fig. 7b). In line with this, YD showed a more stable activity in GPs derived from CTD-resistant cells throughout sensitization induction (Fig. 7b,c). We emphasize that this is the first to test on whether YD exerts similar phenotypic effects in a setting where EGFR is scant and that resistance to gefitinib is relayed collaterally by a prior acquisition of resistance to CTDs. We found that YD effectively diminished AXL protein expression in PTXR- and EBR-derived GPs as detected using antibodies against full-length AXL and its C-terminal fragment, respectively (Fig. 7d and Supplementary Fig. S16a). Accordingly, YD markedly produced soluble AXL evidenced by the increased generation of its N-terminal fragment to the secretion. These results reinforce the idea that PS-RIP-associated degradation of AXL is crucial for developing collateral gefitinib resistance as a consequence of prior paclitaxel or epothilone B resistance and might act as a drug-specific resistance node during a second drug selection.

**Figure 8:**
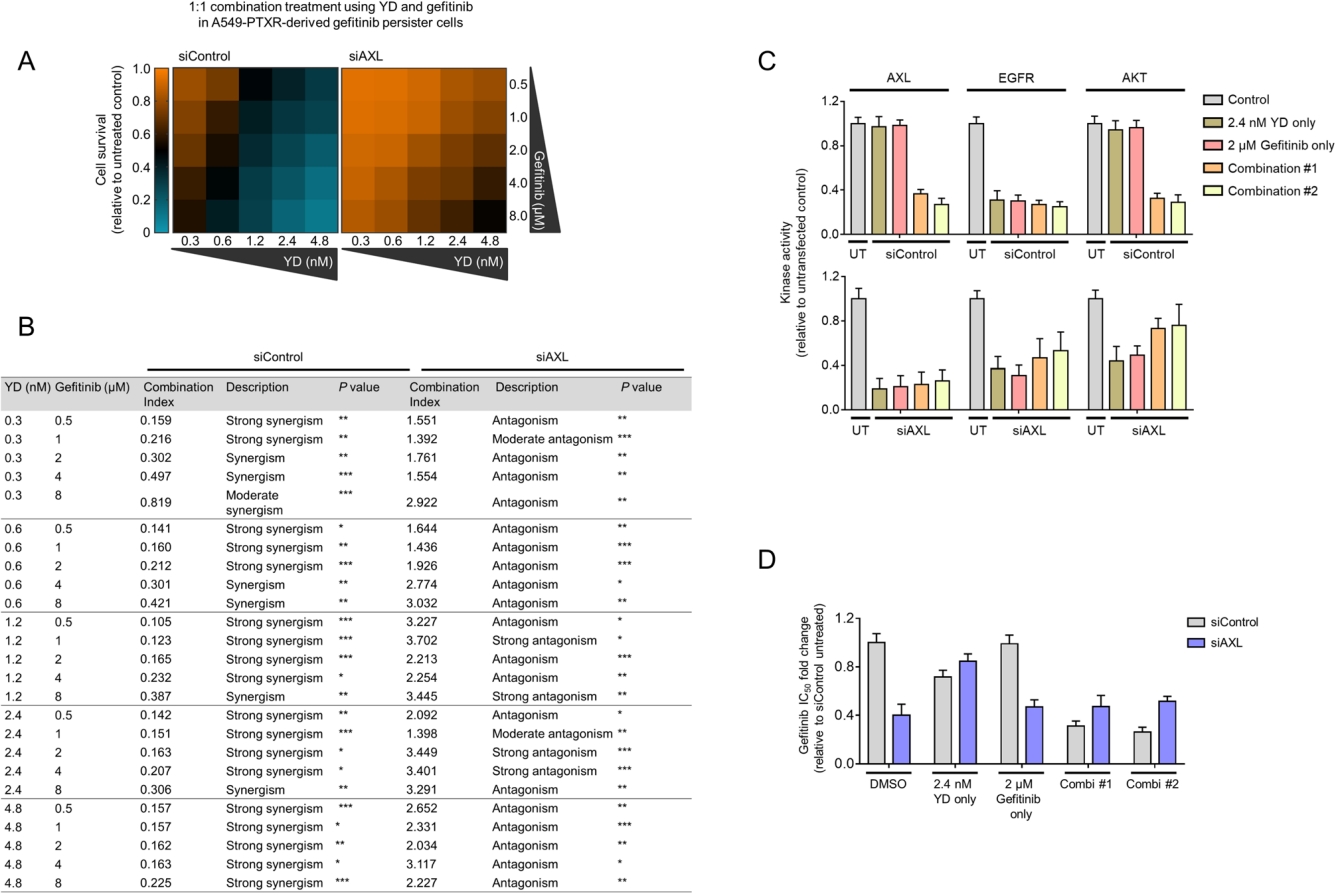
YD in combination with gefitinib achieves synergistic suppression of resistance by inhibiting compensatory mechanisms of AXL. (**a**) Characterization of the combination of YD and gefitinib in A549-PTXR-derived GPs upon AXL RNAi. Cells were treated with the indicated combination of drugs for 48 h. Cell viability was determined by SRB assay and the combination synergy was measured by calculating the combination index values. Values represent mean ± SD of three biological replicates. Python (seaborn; https://seaborn.pydata.org/) was used to generate the plot. (**b**) Combination effects description after combination treatment with YD and gefitinib. *P < 0.05, **P < 0.01, ***P < 0.005, Student’s t test. (**c**) Luminescence-based kinase assay for AKT, AXL, and EGFR in indicated cells, as in a, upon AXL RNAi. Cells were treated with indicated drugs alone or in combination (#1, 2.4 nM YD + 4 μM gefitinib; #2, 4.8 nM YD + 8 μM YD) for 48 h. Values are relative to untransfected control (UT). Values represent mean ± SD of two biological replicates. GraphPad Prism 7.01 was used to generate the plot. (**d**) Characterization of gefitinib resistance in indicated cells as in A upon AXL RNAi. Cells were treated with indicated drugs alone or in combination as in b. Cells were then treated with or without gefitinib for 72 h with a concentration dilution series and were assayed for SRB. Resistance was assessed based on drug IC50 values. Values represent mean ± SD of three biological replicates. GraphPad Prism 7.01 was used to generate the plot. All IC50s were calculated using TableCurve 2D v5.01.

Ligand-dependent and -independent AXL activation concurrently rescued YD-induced killing in PTXR- and EBR-derived GPs (Fig. 7e and Supplementary Fig. S16b). Along with these, YD remains a potent EGFR-TKI based on its capability to target the phosphorylation of EGFR and AXL and exhibiting effective growth inhibitory action in PTXR- and EBR-derived GPs (Fig. 7f). This dependence on AXL also dictates the activity of YD on EMT and CSC phenotypes (Fig. 7g,h). Capacitating its resistance suppression activity, low concentrations of YD synergistically potentiated gefitinib and significantly sensitized PTXR-derived GPs back to gefitinib (Fig. 8a,b). This synergism appeared to be dependent on AXL, as silencing AXL prior to combinatorial treatment dramatically led to antagonism. The synergistic combination of YD and gefitinib also led to significant inhibition of EGFR, AXL, and AKT kinase activities (Fig. 8c), induction of caspase-dependent apoptosis (Supplementary Fig. S17), and suppression of collateral gefitinib resistance (Fig. 8d), all of which displayed as AXL-dependent mechanisms. Thus, overcoming AXL-directed promotion of collateral gefitinib resistance following resistance to CTDs can be achieved by YD alone or in combination with gefitinib. Overall, the use of YD in this study revealed that AXL degradation is a crucial, prolonged process required for CTD-resistant cells to remodel their stress adaptation when transitioning to a GP state.

## Discussion

The history of previous therapy and the response of cancer patients have proven informative in improving their overall survival following sequential treatments^1,11,13,15^. However, the advent of multidrug resistance hampers the success of this sequential therapy strategy. In this study, we report a key mechanism driving collateral gefitinib resistance following failure to prior chemotherapy. We interrogated a large publicly available data resource derived from thousands of annotated drug response of cancer cell lines to examine co-occurring signatures of drug resistance and found that CTDs mostly appear to have co-occurring resistance with the EGFR-TKI gefitinib. As patients undergo an optimal sequence of therapy cycles that can categorically be classified into first- and second-line therapies, we framed our study to investigate whether prior acquired resistance to CTDs can confer collateral resistance to gefitinib. We experimentally showed that among these CTDs, failure to paclitaxel or epothilone B can relay a strong collateral resistance to gefitinib. Based on input features in the GDSC, we found that genomic alterations in two RTKs AXL and EGFR were mostly correlated in co-resistance between a CTD and gefitinib. This pointed us to uncover potential signaling bypass that occurs between these RTKs, marking an advent for drug-specific secondary resistance.

The involvement of AXL in the relapse and metastasis of treatment-treated cancer tumors came as a surprise to us. Although AXL has been shown previously to relay secondary resistance to EGFR and HER targeted therapies in a subset of breast and lung cancers^16,22,23,25^, a marked increase of AXL expression and stability in our small cohort of relapsed breast cancer tumors was unexpected. More importantly, AXL expression was further associated with lymph node and lung metastasis. Together with our biochemical data, we suggest that the role of AXL in conferring secondary resistance depends on its connection with the EMT and CSC programs.

Our lung cancer xenograft data indicate that the link between AXL activation and gefitinib resistance can be specific only to tumors with prior CTD resistance. AXL is implicated as a resistance marker gene in pre-existing sensitive cells that can render therapy resistance to be non-heritable^3,34^. While this non-heritable resistance mechanism is still relatively a new concept, it would be interesting to investigate whether sporadic expression of AXL influences the selection of CTD-resistant cells to a second therapy. Regardless, considering that CTD-resistant cells are successfully selected during gefitinib treatment despite low phosphorylation of EGFR, we hypothesize that high AXL expression is a bypass switch that can re-activate downstream signaling of EGFR.

In an effort to generate models whereby we could differentiate the acquisition of gefitinib resistance in sensitive parental and CTD-resistant cells, we turned to a drug-tolerant persister model of resistance. Under this paradigm, prolonged drug exposure forces a subset of cancer cells to reprogram cellular processes to develop an adaptive resistance strategy whereby drug-tolerant cells can revert to a sensitive state when the presence of the drug is removed. In culture, this resistance aversion is usually observed upon multiple passages during drug holiday. We showed that entry to this transient mode of resistance (herein GPs) allowed CTD-resistant cells to adapt their collateral response to gefitinib. GPs exclusively derived from these cells appeared to depend on the status of AXL as a compensation to a scant EGFR activity because of continued exposure to the EGFR inhibitor compared to GPs derived from parental cells. AXL is also more stable in GPs derived from CTD-resistant cells. AXL is a strong regulator of EMT and CSC phenotypes in these GPs and is widely associated with the co-resistance of gefitinib and 11 CTDs.

AXL, a member of the TYRO3, AXL, and MERTK (TAM) family of RTKs, is an EGFR-diversifying signal that can directly bind to EGFR and other members of HER family like MET and PDGFR^35,36^, owing to its shared homology with these RTKs. This pan-RTK interaction creates a bypass role for AXL to activate alternative downstream signaling cascade for survival of cancer cells from which specific non-TAM RTKs were targeted, enabling resistance selection to specific RTK inhibitors^36,37^. This promotion of resistance upon EGFR-targeted inhibition appears to depend on which downstream signaling the constitutively activated AXL interact with (i.e., AXL-MAPK for cetuximab resistance^38^ or AXL-AKT for osimertinib resistance^39^). Meanwhile, it will be interesting to see whether known transcription factors that regulate AXL synthesis (i.e., activator protein AP1, YAP/TAZ/TEAD, and HIF1α) can mediate the predominant activation AXL and promote EGFR-TKI-specific resistance trajectory in chemoresistant cancer cells. It is important to note that the high GAS6 levels we found to be specific to CTD resistance-enabled GPs might attribute autocrine regulation of other TAM receptors, which we can only argue as venue for further investigation.

While we are the first to report an AXL-dependent logic driving collateral EGFR-TKI resistance following chemotherapy failure, AXL has tightly been linked to multiple mechanisms of multidrug resistance. Complemented by our results on proteolytic rewiring through reduced AXL receptor shedding, it has been shown that kinase inhibitors can block proteolytic shedding of AXL receptor altering the negative feedback on signaling activity and enhances bypass signaling^40^. In addition, a newer EGFR-TKI osimertinib was shown to stimulate AXL by inhibiting this negative feedback loop inducing the emergence of cells tolerant to osimertinib^41^. These EGFR-TKI-tolerant persister cells revealed that a trajectory to a fully-resistant state does not only require a simple AXL-dependent bypass signaling but can also be characterized by dependence to an increased autophagic flux^42^.

Our previous and current data^32,33^, together with that of others^39–41,43–45^, point that an extended degradation of AXL is a pivotal mechanism of collateral EGFR-TKI resistance. In probing this process, we used YD, a diterpene ester our labs^46,47^, together with others^48,49^, previously isolated from a medicinal plant. YD is a candidate AXL degrader which also exhibits targeting action on SerpinB2, NNMT, and BMP4 implicated in lung cancer^32,33,50–52^. Our study adds to this compendium of evidence that YD can suppress resistance by inducing near-complete proteolysis (i.e., via PS-RIP) of AXL and operate its AXL degradation activity at sub-stoichiometric levels (low nanomolar). While it might appear that the activity characterization of YD in our experiments reflect similar conclusions from our previous works^32,33^, we took advantage of YD to probe AXL degradation and provide potential direction for pharmacological targeting of collateral resistance evolution to EGFR-TKIs following chemotherapy failure. Given that our findings imply a mainly proof-of-principle study, we caution the direct clinical interpretation of our results and acknowledge the fact that more clinically-relevant systems (i.e., patient-derived animal models) will help facilitate translation. Regardless, our work highlights that secondary persistence to an EGFR-TKI following prior stable resistance to CTDs can evolve through the activation and stability of AXL during the emergence of non-genetic persistence, which can be targeted using a natural product-based degrader.

## Methods

### Data reporting and statistics

Data are expressed as mean ± standard deviation (SD) (or median ± interquartile range), unless otherwise specified. No statistical method was used to predetermine sample size. Group allocation and outcome assessment were not performed in a blinded manner. Statistical tests were performed using GraphPad Prism 7.01 and TableCurve 2D v5.01 software (Systat). Significant differences between experimental groups were determined using an unpaired two-tailed Student *t*-test assuming Gaussian distribution. For all analyses, *p*-values < 0.05 were considered statistically significant. Data visualization were mostly done using Python (with referenced libraries; see code availability), GraphPad Prism 7.01 and R in conjunction with ggplot2 software package (https://ggplot2.tidyverse.org/).

### Bioinformatics

Co-resistance signature of drugs shared in cancer cell lines was investigated using the GDSC database (www.cancerRxgene.org), a publicly available resource for information on drug sensitivity and molecular markers of drug response in cancer cell lines. Collaterally shared sensitivity signatures of drug pairs were derived from the binarized sensitivity (sensitive or resistant) data set of the screened compounds (n = 265) in 1001 cancer cell lines based on their IC50 values in the form of activity area values (mined from pre-processed reference data in the GDSC). Categorical grouping of cell lines per CTD ↔ gefitinib co-resistance was done and plotted in R (see code availability). All IC50s are expressed in μM concentration. The discretization threshold for each drug (log IC50/cell line) was determined by applying the waterfall method that identifies the cutoff based on Pearson correlation such that low values would correspond to ‘resistant’ while high values to ‘sensitive’. The cutoff for sensitive or resistant classification was somewhat selected arbitrarily because of the maximum dose tested. Cell lines without corresponding drug and expression measurements were not considered in the analysis. All co-resistance and expression/genomic alteration association analyses were done by quantitatively matching pre-processed values with binarized drug response of each cell line. Matched data were plotted as relative expression or frequencies unless otherwise specified. For co-resistance frequency normalization, we used min–max feature scaling in order to rescale the co-resistance counts to be in the range [0,1]. All relative quantification and plotting were done in Python (see code availability). All information on how each oncogenic alteration was derived has been described previously^12^. All analyzed basal gene expression data was derived from raw microarray data deposited in ArrayExpress (accession number: E-MTAB-3610)^12^. In exploring the correlation of AXL gene expressions with drug sensitivity, a previously developed in silico prediction interface utilizing data from the CCLE and GDSC databases was used^20^. For survival analysis, the data were queried through KM plotter for lung cancer and breast cancer in http://kmplot.com/analysis, using the 2017/18 version of the database. For metastasis correlation, the data were queried through the HCMD in http://hcmdb.i-sanger.com, using the 2019 version of the database. For co-occurrence gene analysis, data from the pan-cancer MSK-IMPACT, pan-lung cancer TCGA, and breast cancer METABRIC clinical cohorts were used. The co-occurring genes in patients with AXL copy number-amplified, -deep deleted, AXL mRNA-upregulated or -downregulated expressions were stratified. Then, number of genes co-occurring with the EMT and CSC phenotypes were identified. To do so, the functionally annotated genes in the dbEMT (http://dbemt.bioinfo-minzhao.org/) and CSCdb (http://bioinformatics.ustc.edu.cn/cscdb/) databases, which are comprehensive gene resources for EMT and CSC, respectively, were used. These co-occurring EMT/CSC genes with top 1000 upregulated gene hits in 11 co-resistance cases were further compared.

### Cell culture

Human A549, H1299, H1993, H292, H358, and H460 cells from ATCC were cultured in RPMI-1640 media (Welgene) and mouse LLC1 cells from ATCC were cultured in DMEM/F12 media (Gibco). PC9 cells, originally provided by Jin Kyung Rho (Asan Medical Center, University of Ulsan, Seoul, Korea), were cultured in RPMI-1640 media. All media contained 10% FBS, 2 mM L-glutamine, 100 IU ml^−1^ penicillin/streptomycin (Invitrogen). All cells were obtained in 2014, 2015, and 2017 and passaged 4 to 20 times after obtaining. All cells were grown in a humidified incubator at 37 °C with 5% CO_2_ and were tested regularly for mycoplasma contamination. A549, H1993, and PC9 cells were authenticated at the College of Pharmacy, Seoul National University and LLC1 cells were authenticated at the College of Veterinary Medicine, Seoul National University.

### Generation of CTD-resistant cells

To generate paclitaxel- (PTXR), docetaxel- (DTXR), and epothilone B- (EBR) resistant cancer cell lines, parental cells were seeded at low density and exposed to 0.005 ~ 5 μM of indicated drugs (LC Labs). After approximately > 3 (high fold; transient resistance) to > 20 (high fold; stable resistance) weeks of continuous exposure to escalating drug concentrations, we derived resistant clones that were maintained on indicated drugs (10 nM for transient and 1 μM for stable resistance). Prior to maintenance culture, only those that form colonies upon selection were maintained. Resistance stability was characterized upon subjecting resistant clones to drug holidays (15 to 45 days). Resistance status was determined by fold change in IC50 values for each drug in resistant clones compared to parental cells. Cells were passaged every four days with fresh media containing drug.

### Generation and expansion of pre-GPs and GPs

A549-, H1993-, and PC9-derived cell lines which have varying sensitivity profiles to gefitinib at the parental state were used in this study. This allowed us to recover a sufficient surviving populations following harsh therapeutic pressure due to exposure to high gefitinib concentrations at the initial selection. At the first round, 5 × 10^5^ cells were plated in 150 mm plates and allowed to adhere for 24 h. Cells were then treated with IC75 concentrations of gefitinib in respective cell lines for two rounds, each treatment lasting 72 h. Surviving fractions of cells were then recovered by treatment with 0.5 μM (for PC9-derived clones) or 2 μM (for A549- and H1993-derived clones) gefitinib until colonies appeared. At the end of this recovery stage, few isolated drug-tolerant colonies with slow growth kinetics were remained on the plates. Clearly well-spaced colonies were picked and allowed to adhere for 24 h in drug-free media. These colonies were then subjected to expand in 1–4 μM gefitinib initially for ~ 2.7 weeks. Fresh media containing drug were replaced every 3 days until drug-tolerant colonies started to re-emerge. Around ~ 20 ‘individual’ colonies (> 40 visible cells per colony) were isolated, transferred to 96-well plates, and individually expanded (one colony per well) in 1 or 2 μM gefitinib-containing media. Cells that were not able to expand in this format tested strong positivity to senescence-associated β-galactosidase while those that were able to expand were completely negative (data not shown). Cells were subsequently transferred to 6-well plates for continued expansion. Plate transfers were performed only when cells were at least > 40% confluent. Cells were periodically examined and found negative for mycoplasma. Stock cultures of expanded populations from this method were considered as ‘gefitinib persisters.’ ‘Sensitized gefitinib persisters’ refer to GPs that were continuously grown in drug-free culture for indicated times (usually > 30 days unless otherwise stated). ‘Rederived gefitinib persisters’ refer to sensitized GPs that were subsequently grown in 1–4 μM gefitinib-containing media for at least a week. We note that gefitinib media was used for the entire duration of persister generation and expansion. Expanded cells were only frozen when they reached full confluence in 6-well plates.

### Animal studies

All animal use and care followed the guidelines approved by the Institutional Animal Care and Use Committee of KAIST (through GSMSE) and Seoul National University (through College of Veterinary Medicine). For nude mice models, 4–5 week-old male athymic mice (BALB/c-nu) were purchased from Orient Bio Inc. and were transferred, established, and bred in an animal facility at GSMSE, KAIST. A549- and PC9-derived cell suspensions (1.2 ~ 1.4 × 10^7^ cells) in 200 μL culture medium/growth factor-reduced Matrigel (BD Biosciences) in a 1:1 ratio were subcutaneously injected into the right flank of each mouse. Two-to-four sites on the flanks were injected per mouse. Mice were treated when their tumor volumes reached 70 to 100 mm^3^ (gefitinib cohort) and 100 to 150 mm^3^ (paclitaxel cohort). Based on the lethality and body weight loss exhibited by the nude mice, they were randomized into vehicle control and treatment groups of four to five animals per cohort. Gefitinib (100 and 200 mg/kg body weight) and paclitaxel (10 mg/kg body weight) were dissolved in 200 μL vehicle solution (Tween 80-EtOH-H2O in 1:1:98 ratio) and were orally administered to mice in appropriate cohorts once daily for 20 days (paclitaxel cohort) and 14 days (gefitinib cohort), respectively. The control group was treated with an equal volume of vehicle. Mice were sacrificed and tumors were excised and flash frozen at the end of each experiment schedule.

For the metastasis model, 7–8 week-old C57BL/6 male mice were purchased from Orient Bio Inc. and were transferred, established, and bred in an in-house animal facility at the College of Veterinary Medicine, Seoul National University. LLC and LLC-PTXR cell suspensions (1.5 × 10^6^ cells) in 140 μL culture medium were intravenously injected through the tail vein. Mice were sacrificed at days 15 and 37 after injection. Lung, liver, and spleen that developed primary or metastasized tumors were collected in ice-cold PBS for further testing. All animals were fed with free access to standard diet (PMI LabDiet) and water. All mice were maintained under continuous sedation by administering 2–4% isoflurane via an anesthesia mask during surgery and prior to euthanasia.

### Preparation of YD

Fresh stock of YD was prepared from CHCl_3_-soluble fraction of the flowers of *Daphne genkwa*, characterized as natural source of diterpenes with wide-ranging antitumor activities, as described previously^46^. Preliminary extracts and fractions were prepared from the dried powder of the flowers and the most active fraction (10% MeOH/CHCl_3_) was further fractionated and the final fraction was prepared by HPLC using octadecyl silica (ODS) column with elution of 93% MeOH in water (340 mg; 0.0034%). YD appeared as white amorphous powder. Purity (> 98.5%), physical, and spectral data were previously described^46,47^.

### Cell proliferation and survival assays

Cell proliferation and survival were monitored by sulforhodamine B (SRB) and colony formation assays, as we previously described^32,53^. For validation, 3-(4,5-dimethylthiazol-2-yl)-2,5-diphenyl-2H-tetrazolium bromide (MTT) colorimetric assay was simultaneously used along with SRB assay, as we previously described^54^. IC10, IC25, IC50, and IC75 values were calculated via non-linear regression analysis using TableCurve 2D v5.01 software (Systat). For colony assay, 600 ~ 800 cells were seeded in 24- or 6-well plates and allowed to expand for 3 days in drug-free media. Drug treatment started at day 3 and lasted for 4 days and media was replaced and colonies were further incubated for additional 7 days in drug-free media. Colonies > 0.20 mm diameter were counted and quantified using the ColonyArea plugin in Fiji/ImageJ (NIH software). Cell confluence was determined using an automated cell counter (Luna-II, Logos Biosystems).

### BrdU incorporation assay

A colometric BrdU incorporation assay (Abcam) was done to measure the rate of DNA synthesis according to manufacturer’s instructions. Briefly, cells were seeded in 96-well plates and subjected to treatment protocols. BrdU is added to cells followed by 4 h incubation to incorporate BrdU into the DNA of proliferating cells. Culture supernatant was removed followed by fixation. Cells were then incubated with an anti-BrdU antibody conjugated to peroxidase. Bound BrdU is detected by a substrate reaction and quantified by absorbance measurement at 350 nm with common background subtraction in a microplate reader (Varioskan LUX, Thermo Scientific).

### Holotomography

The 3D label-free images of fixed or live cells were performed using HT. To measure the 3D RI tomograms of cells, we used a commercial system (HT-1H; Tomocube Inc.; South Korea). The used HT system is based on interferometry equipped with a digital micromirror device for the control of an illumination beam. A sample is illuminated with a coherent plane wave (wavelength = 532 nm) with various illumination angles (49 different angles). The corresponding multiple 2D optical field images of the sample are measured using off-axis Mach–Zehnder interferometry. From the measured multiple 2D optical fields, the 3D RI distribution of the sample is reconstructed by inversely solving Helmholtz wave equation^55^. The theoretical spatial resolution was 110 nm and 360 nm for lateral and axial direction, respectively. The details on the principle, the optical system, and the resconstruction algorithm can be found elsewhere^56,57^. Visualization of the 3D iso-surface and processing of 3D RI tomograms were conducted using a commercial software (TomoStudio; Tomocube Inc.; South Korea). Supplementary Movies 1 and 2 display both 2D and reconstructed 3D RI distributions of live A549 and A549-PTXR cells, respectively.

### Immunoblotting and immunoprecipitation

For immunoblotting, 2–3 million cells per mL were lysed in 2 × SDS loading buffer (250 mM Tris–HCl pH 6.8, 4% SDS, 10% glycerol, 0.006% bromophenol blue, 2% β-mercaptoethanol, 50 mM sodium fluoride, and 5 mM sodium orthovanadate) and boiled for 5 min. Protein samples (usually 30–50 μg) were separated on 10–12% SDS-PAGE gels and transferred to PVDF (Millipore) or nitrocellulose (Bio-Rad) membranes using a semi-dry transfer system (Amersham). Membranes were blocked with 5% bovine serum albumin (BSA) in Tris-buffered saline containing 0.1% Tween-20 (TBST) for 1 h at room temperature and then incubated with primary antibodies in 2.5% BSA in TBST overnight at 4 °C. Membranes were washed multiple times with TBST and incubated with the corresponding horseradish peroxidase-conjugated secondary antibodies (Bio-Rad) antibodies diluted in 2.5% BSA in TBST for 2 h at room temperature. After washing, blots were visualized using ECL Plus Western Blotting Substrate (Thermo Scientific) and ChemiDoc Imaging System (Bio-Rad).

For immunoprecipitation, cell supernatants were filtered through a 0.2-μm filter and were admixed with lysis buffer (50 mM Tris–HCl [pH 7.4], 150 mM NaCl, 1 mM EDTA, 0.5% NP-40) containing protease and phosphatase inhibitors (Roche). Supernatant lysates were precleared for 30 min at 4 °C with Protein G Sepharose 4 Fast Flow (GE Healthcare). After removal of the beads (10 min, 12 000 rpm, 4 °C), the supernatant was incubated with the indicated antibody overnight at 4 °C. The immunocomplex was collected with beads at 4 °C for 2 h. The beads were washed multiple times with the lysis buffer, and the bound proteins were eluted with 2 × Laemmli sample buffer at 95 °C for 5 min. Protein concentrations were determined by Bradford protein assay (Bio-Rad). The samples were subjected to blotting as described above. Primary antibodies used were: pEGFR^(Y1068)^ (ab5644, abcam), EGFR (ab52894, abcam), pMET^(Tyr1349)^ (#3121, CST), MET (#8198, CST) pAKT^(Ser473)^ (#9271, CST), AKT (#9272, CST), pERK1/2^(Thr202/Tyr204)^ (#4370, CST), ERK1/2 (4696, CST), AXL (H-3, Santa Cruz), C-terminal AXL (OAAB17122, Aviva Systems Biology), N-terminal AXL (OAAB17121, Aviva Systems Biology), cleaved PARP (#5625, CST), α-tubulin (#2144, CST), and GAPDH (6C5, Santa Cruz).

### Immunohistochemistry

For histological evaluation of lung tumor metastases in our LLC mouse model, tissues were harvested, formaldehyde-fixed and paraffin-embedded (FFPE) following standard procedures and consecutive sections were prepared. Lung tissue sections were stained with H&E to define tumor tissue areas in the lung as previously described. Five regions of interest (n = 4) on paraffin-embedded H&E-stained sections were defined at 20 × and imaged for tumor cell clusters. Representative images are shown. For immunohistochemical analysis of patient tumors, FFPE tissue slides were dewaxed in xylene, rehydrated through gradients of alcohol, and placed in an endogenous peroxide block before heat-induced epitope retrieval (HIER) prior to immunostaining. The sections were incubated in 10 mM Tris (pH 9.0) or 10 mM sodium-citrate (pH 6.0) buffered solution containing 0.05% Tween and, depending on the Ab used, if needed heated at 100 °C for 10 min using a pressure cooker. The sections were pretreated using BLOXALL endogenous enzyme blocking solution (Vector Laboratories) for 10 min to destroy all endogenous peroxidase activity. Non-specific staining was blocked by treating sections with 10% normal donkey serum. The sections were then incubated with anti-AXL antibody (ab227871, abcam; 1:350 dilution) for 4 h followed by several washes. Appropriate biotinylated link and horseradish peroxidase (HRP)-conjugated secondary antibodies were applied onto the sections and were further incubated for up to 2 h in a dark humidified chamber at room temperature followed by washing. Immunohistochemical staining was carried out using LSAB^+^ System-HRP kit (Dako). The sections were counterstained with hematoxylin as indicated, and dehydrated in alcohol gradient series, and were mounted using an organic mountant. The positive staining density was measured using a computerized imaging system composed of a Leica CCD camera connected to a Leica DMi1 microscope (Leica Microsystems). The H-score scoring system was used, which evaluated staining intensity (0 to 3) and the percentage of positively stained cells (0 to 1), with a final score ranging from 0 to 3.

### RNA extraction, RT-PCR, and qPCR

Total cellular RNA was extracted using TRIzol (Thermo Scientific) following manufacturer’s protocol. Total RNA was mixed with 1 µl of 10 mM dNTPs (Thermo Scientific) and 1 µl of 50 µM random hexaprimers (New England Biolabs) and treated with DNase I (Takara) and reverse transcribed using RevertAid (Thermo Scientific) or SuperScript IV reverse transcriptase (Thermo Scientific). The gene specific reverse transcription program recommended by the manufacturer was used to synthesize cDNA. cDNA was amplified by SYBR Green PCR master mix (Bioline) and analyzed by AriaMx Real-Time PCR System (Agilent). Each reaction was performed in three replicates. Fold changes were calculated by the ΔΔC_t_ method using GAPDH as internal standard, and normalized to the experimental control as indicated. Primers were tested for specificity by performing a test PCR reaction and resolving the samples on an agarose gel with ethidium bromide. Primers used in this study are provided in Supplementary Table S1.

### RNAi and plasmid transfections

All short-interfering RNA (siRNA) and plasmid transfections were performed using RNAiMAX (ThermoFisher), DharmaFECT 4 (Dharmacon), or Lipofectamine 3000 (ThermoFisher) according to manufacturer’s instructions and analyzed 48 h post transfection. In each assay, a uniform transfection protocol was adapted. Cells were harvested by trypsinization, resuspended in fresh medium and transferred into a glass conical tube and passed through a 35 μm cell strainer cap to prevent aggregation prior to seeding. Cells were seeded at 4–8 × 10^4^ cells per well of 6-well clear flat bottom, non-treated polystyrene plate (Corning) in supplemented OptiMEM media. During transfection, media was changed to nutrient-deprived (5% dialyzed FBS) OptiMEM media. Cells were recovered in normal media thereafter. For transient knockdown of AXL, 125–250 pmol of 25-bp siRNA duplexes were used and purchased from IDT Korea (Integrated DNA Technologies). Sequences used for AXL RNAi are: sense; 5′-CCA GCA CCU GUG GUC AUC UUA CCU U-3′ and antisense; 5′-AAG GUA AGA UGA CCA CAG GUG CUG G-3′. Scrambled duplexes were used as non-targeting siRNA control (siControl). RNAi efficiency was measured by qPCR and Western blotting (Supplementary Fig. S18).

For AXL overexpression, myc-His tagged AXL in a pcDNA3.1 vector (2 μg of purified plasmid) was used for transfection. The vector was constructed by cloning AXL cDNA into the EcoRI and BamHI sites of the backbone vector. For validation studies, untagged AXL in pIRESpuro2 (10 μg DNA) was used. All ectopic DNA transfections were performed 18–48 h post-transfection.

### ATP measurement

Cells were seeded in 96-well plates and were subjected to indicated treatment/culture conditions all in nutrient-restricted media (10% FBS dialyzed against 0.15 M NaCl until < 5 mg/dL glucose, 10,000 MW dialysis tubing, and no L-glutamine). ATP levels were measured using the luminescence-based ATPLite system (Perkin-Elmer) following manufacturer’s instructions.

### ELISA-based phosphorylation and kinase assays

For detecting Met pan-tyosine phosphorylation, sandwich ELISA-based PathScan kit (CST) was used according to manufacturer’s instructions. For scalable detection of kinase activities of Met, Axl, Egfr, and Akt, the following kits were used following manufacturer’s instructions: luminescence-based MET and AXL kinase enzyme systems (Promega) and solid phase sandwich ELISA-based PathScan kits for EGFR and AKT1. For total AXL or AXL phosphorylation measurement, we performed a multiplexed ELISA using individually identifiable beads (Bio-Rad) as described previously^58^. Capture antibody for AXL (R&D Systems) and biotinylated anti-phosphotyrosine antibody 4G10 (Millipore Corp.) were used. AXL receptor abundance was quantified by comparison with a recombinant standard (R&D Systems).

### Caspase activity assays

Caspases 3/7 and 9 activities were assessed using a fluorescence based Apo-ONE Homogeneous Caspase-3/7 Assay Kit (Promega) and luminescence-based Caspase-Glo 9 Assay System (Promega), respectively, following manufacturer’s protocol. Briefly, 2.5 × 10^4^ cells were seeded onto 96-well plates, allowed to adhere overnight, and treated according to indicated drug schedules. 120 μL of master reagent (mix of kit’s substrate 170 and buffer) was loaded onto each well, gently mixed in a shaker for 1 min, and incubated for 40 min to 90 min at RT. Excitation and emission wavelengths were set at 560 and 590 nm, respectively. Luminescence was read on POLARstar Omega luminometer.

### Ex vivo biochemical analysis

Excised portions of frozen tumors and primary/metastasized tumor nodules in organs from mice were thawed on ice and homogenized in Complete Lysis Buffer (Active Motif) using a handheld homogenizer. Tumor lysates were subjected to immunoblotting and phosphorylation assay as described. Whole RNA from tumor samples was isolated from cells using RNeasy Mini Kit (QIAGEN) according to manufacturer’s instructions and was subjected to RT-PCR as described. Primary lung tumors were excised and directly fixed in 4% paraformaldehyde and embedded in paraffin. Sectioned slides of embedded specimens were subjected to immunohistochemistry as described.

### Human tumor samples and ethics statement

All human tissue samples were collected and analyzed with approved protocols in accordance with the ethical requirements and regulations of the Institutional Review Board of Seoul National University Hospital. Informed consent was obtained from all subjects prior to the conduct of the study. Thirty-three adult patients, all clinically diagnosed to have invasive ductal carcinoma, received adjuvant chemotherapy consisting of four cycles of adriamycin (doxorubicin) plus cychlophosphamide regimen followed by four additional cycles of paclitaxel. Post- or preoperative radiation therapy was not performed. All clinicopathologic information including recurrence/relapse and metastasis status of patients were obtained by reviewing medical records, pathology reports, and evaluating H&E-stained sections of biopsied tumor tissues. All patient data were independently reviewed by a breast cancer pathologist (co-author H.S.R.).

## Supplementary Information

Supplementary Information 1.

Supplementary Movie S1.

Supplementary Movie S2.

## Data Availability

All data needed to evaluate the conclusions in the paper are present in the paper and/or Supplementary Information. The main scripts used for the presented analyses are available upon request from the corresponding authors or from https://github.com/borrisHUBO/Aldonza-et-al.-Scientific-Reports. All reference data, pre-processed and raw drug screening data from the GDSC we used are available in the 2018 version of the site (https://www.cancerrxgene.org/gdsc1000/GDSC1000_WebResources/Home.html).
