# Supplementary material for: Chemotherapy confers a conserved secondary tolerance to EGFR inhibition via AXL-mediated signaling bypass
Source: Sci Rep. 2021 Apr 13;11:8016. doi: 10.1038/s41598-021-87599-9 (PMC8044124; doi:10.1038/s41598-021-87599-9)

Supplementary Materials for

# Chemotherapy confers a conserved secondary tolerance to EGFR inhibition via AXL-mediated signaling bypass

Mark Borris D. Aldonza[1,2,3,4,5], Roben D. Delos Reyes[6], Young Seo Kim[1,7], Jayoung Ku[1,3], Ana Melisa Barsallo[1,2], Ji-Young Hong[8], Sang Kook Lee[8], Han Suk Ryu[10], YongKeun Park[3,7,9], Je-Yoel Cho[4,5,*], and Yoosik Kim[1,3,*]

[1]Department of Chemical and Biomolecular Engineering, Korea Advanced Institute of Science and Technology (KAIST), Daejeon 34141, Korea.

[2]Department of Biological Sciences, KAIST, Daejeon 34141, Korea.

[3]KI for Health Science and Technology (KIHST), KAIST, Daejeon 34141, Korea.

[4]Department of Biochemistry, College of Veterinary Medicine, Seoul National University, Seoul 151-742, Korea.

[5]BK21 PLUS Program for Creative Veterinary Science Research and Research Institute for Veterinary Science, Seoul National University, Seoul 151-742, Korea.

[6]Department of Electrical Engineering, KAIST, Daejeon 34141, Korea.

[7]Tomocube Inc., Daejeon 34051, Korea.

[8]College of Pharmacy, Natural Products Research Institute, Seoul National University, Seoul 08826, Korea.

[9]Department of Physics, KAIST, Daejeon 34141, Korea.

[10]Department of Pathology, Seoul National University Hospital, Seoul National University College of Medicine, Seoul 03080, Korea.

*Correspondence: ysyoosik@kaist.ac.kr (Y.K.); jeycho@snu.ac.kr (J.Y.C.)

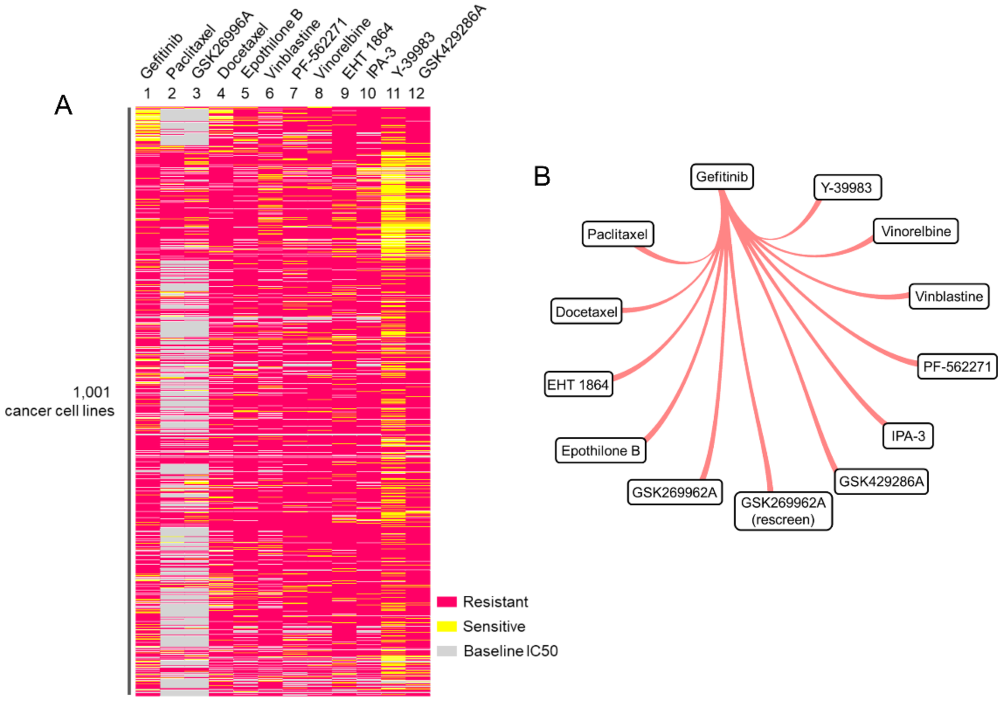


Antimitotic drug → Gefitinib

| | Co-resistance with gefitinib (%) |
|---|---|
| Paclitaxel | 92.77 |
| GSK269962A | 90.9 |
| Docetaxel | 87.69 |
| Epothilone B | 87.16 |
| Vinblastine | 86.29 |
| PF-562271 | 86.11 |
| GSK269962A (rs) | 85.63 |
| Vinorelbine | 85.59 |
| EHT 1864 | 85.57 |
| IPA-3 | 85.12 |
| Y-39983 | 84.0 |
| GSK429286A | 83.94 |

**Supplementary Fig. S1. Co-resistance signatures between gefitinib and 11 CTDs.**
(**a**) Discretization threshold of binarized drug response (sensitive or resistant) of gefitinib and 11 CTDs across 1,001 cells lines analyzed in the GDSC. GraphPad Prism 7.01 was used to generate the plot.
(**b**) Trajectory visualization of co-resistance to gefitinib in cancer cell lines classified as resistant to indicated antimitotic drugs processed from publicly available database of the Genomics of Drug Sensitivity in Cancer (GDSC). Note that co-resistance is viewed as one-directional co-occurring resistance trajectory as indicated identified in the pre-processed data files of binarized response of cell lines to drugs in the GDSC. Beside is the table showing co-resistance with gefitinib in cell lines classified as resistant to indicated antimitotic drugs. R [59] (ggplot2; https://ggplot2.tidyverse.org/) was used to generate the plot.

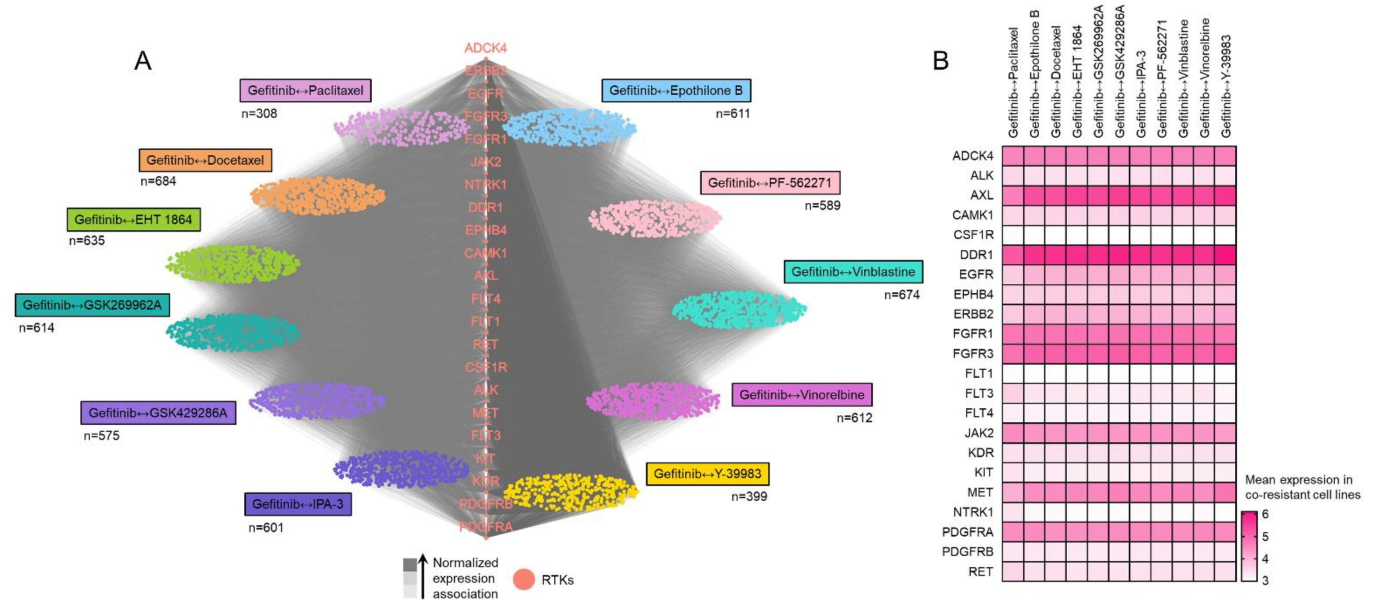


**Supplementary Fig. S2. Co-resistance between EGFR-TKI gefitinib and CTDs can be traced by RTK expression.**

(**a**) Association of the expression of 22 RTKs with individual cell lines classified to be co-resistant to gefitinib and a CTD (11 gefitinib ↔ CTD co-resistance cases evaluated in this study). Color of edges represents the normalized gene expression association while the nodes represent individual cell line component. Python (seaborn; https://seaborn.pydata.org/) was used to generate the plot.

(**b**) Mean gene expression profile of indicated RTKs in per co-resistance cases. GraphPad Prism 7.01 was used to generate the plot.

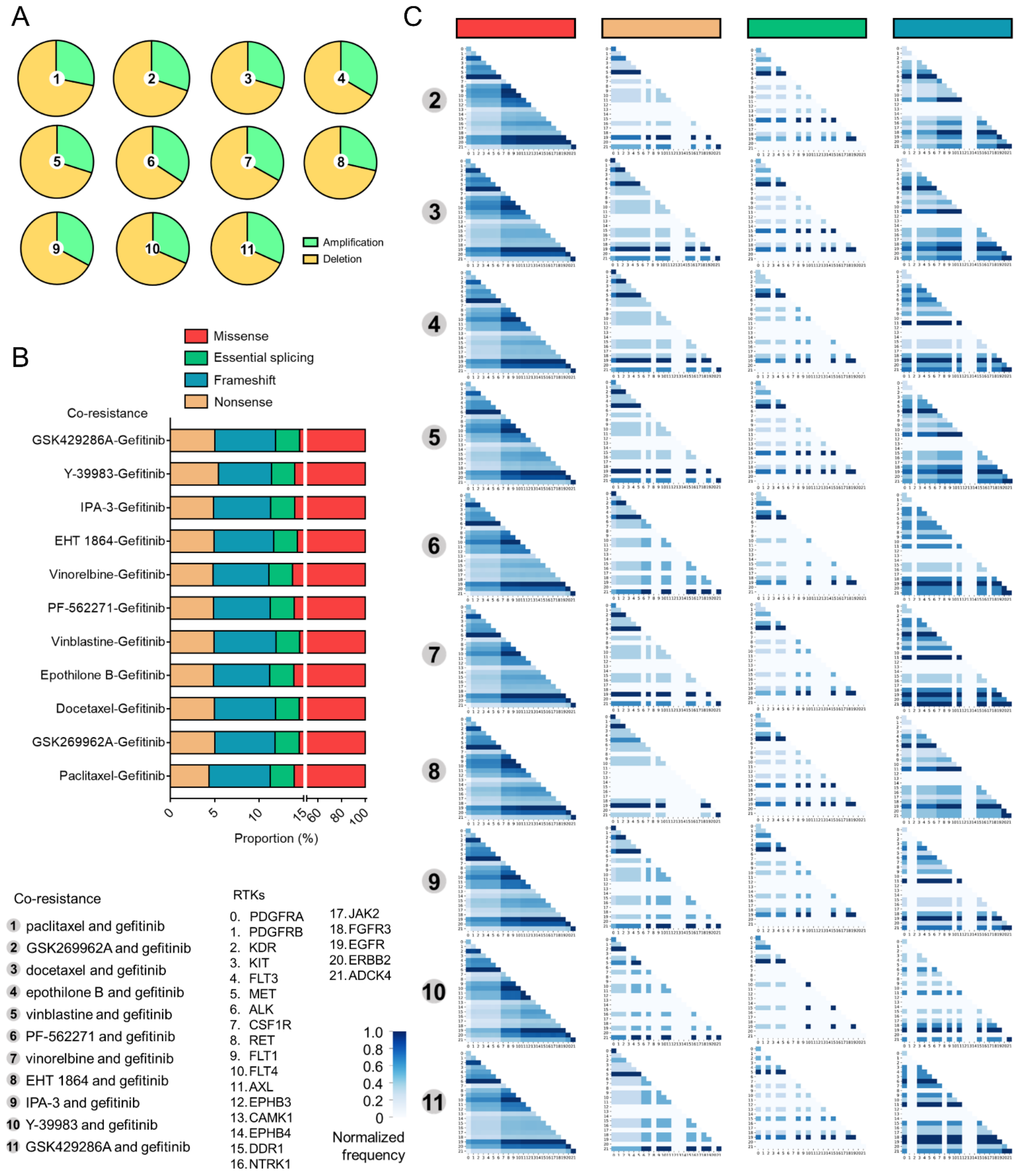


**Supplementary Fig. S3. Mutational signatures associated with co-resistance between gefitinib and 11 CTDs.**

(**a**) Proportion of RACS amplification and deletion per co-resistance case.

(**b**) Proportion of each mutation type (missense, nonsense, essential splicing, and frameshift) per co-resistance case.

(**c**) Association matrix of missense, nonsense, essential splicing, or frameshift mutation frequency (in all screened genes) between 22 RTKs per co-resistance case. Similar denomination of indicated mutation per gene in each cell line was normalized. Python (seaborn; https://seaborn.pydata.org/) was used to generate all plots.

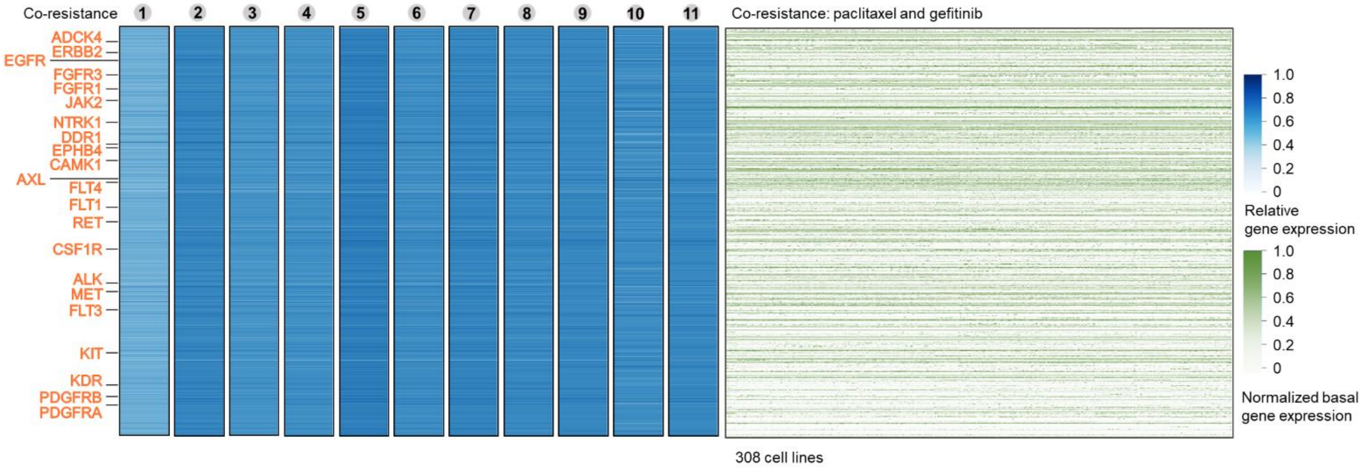


**Supplementary Fig. S4. Relative gene expression profile of about 17,000 genes per co-resistance case.**
22 RTKs are highlighted. Normalized gene expression in each cell line co-resistant to paclitaxel and gefitinib is also shown. Python (seaborn; https://seaborn.pydata.org/) was used to generate the plot.

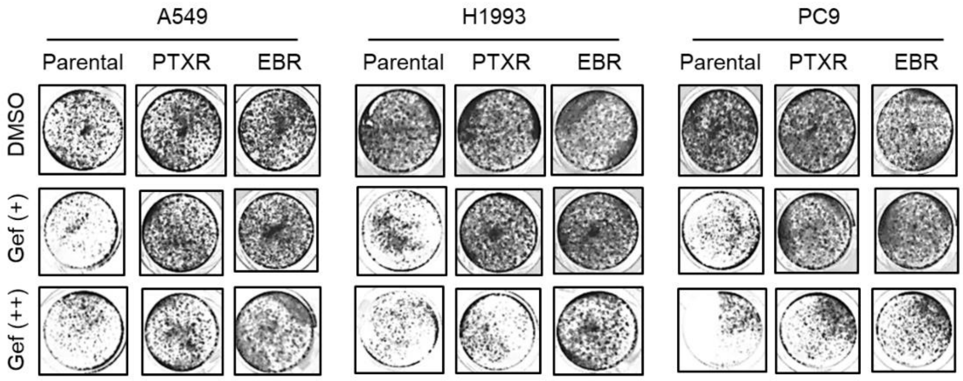


**Supplementary Fig. S5. Validation of collateral gefitinib resistance in CTD-resistant cells.**
Colony formation of indicated parental and CTD-resistant cells. Cells were treated with or without 4 μM or 8 μM (A549-derived cells), 2 μM or 4 μM (H1993-derived cells), and 0.5 μM or 1 μM (PC9-derived cells) of the drug for 4 days followed by drug-free culture for 8 days. Representative of two independent experiments.

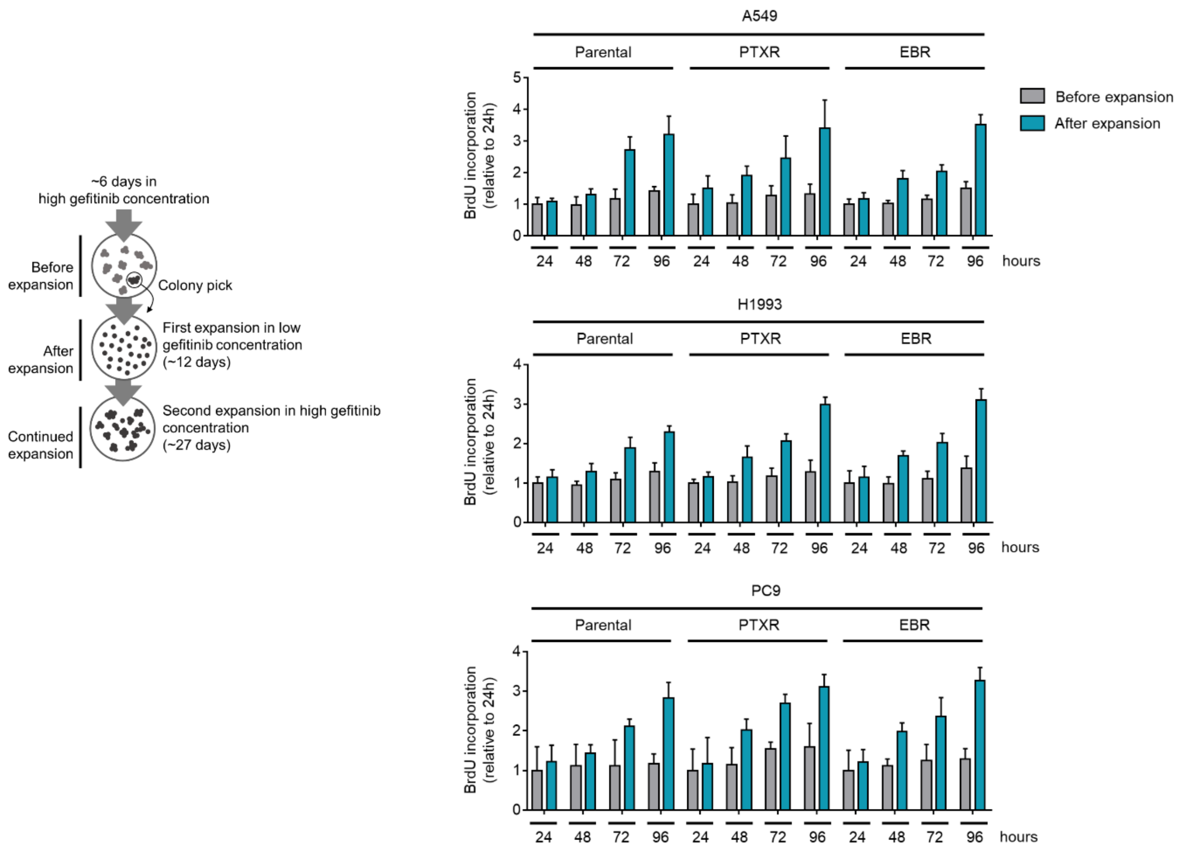


**Supplementary Fig. S6. DNA synthesis of pre-GPs.**
BrdU incorporation assay in indicated pre-GPs. Cells were assayed before and after expansion as schematized in the left panel (mean±SD of three biological replicates). GraphPad Prism 7.01 was used to generate the plot.

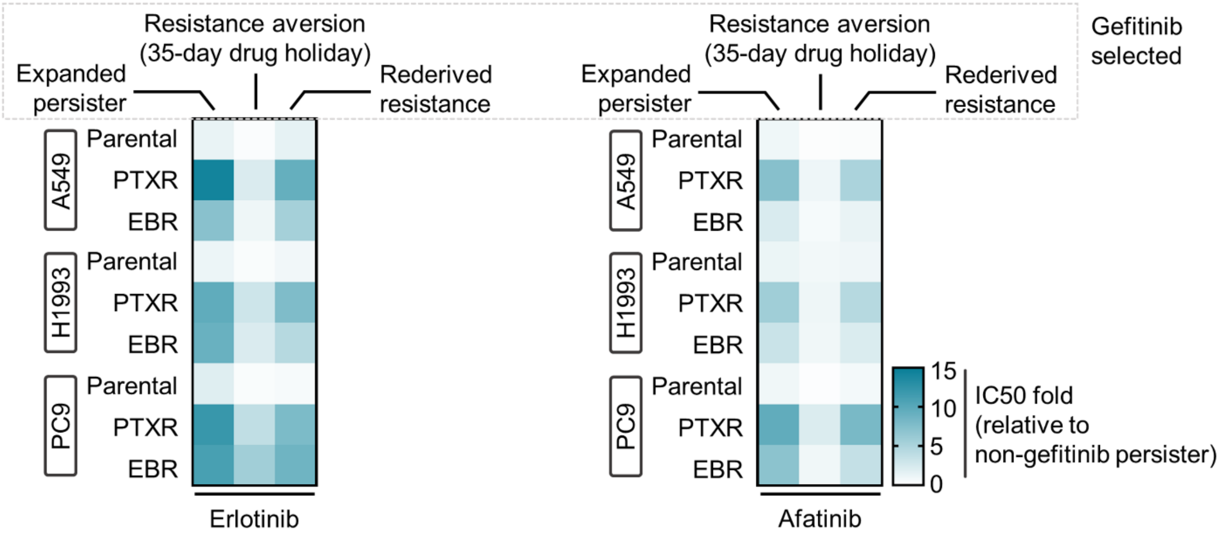


**Supplementary Fig. S7. Collateral resistance to other EGFR-TKIs in GPs.**
Characterization of collateral drug tolerance in A549-, H1993-, and PC9-derived GPs (similar selection under gefitinib as Fig. 3g) in response to EGFR-TKIs erlotinib or afatinib. Cells were treated with or without drugs for 72 hours with a concentration dilution series and were assayed for SRB (mean±SD of three biological replicates). GraphPad Prism 7.01 was used to generate the plot.

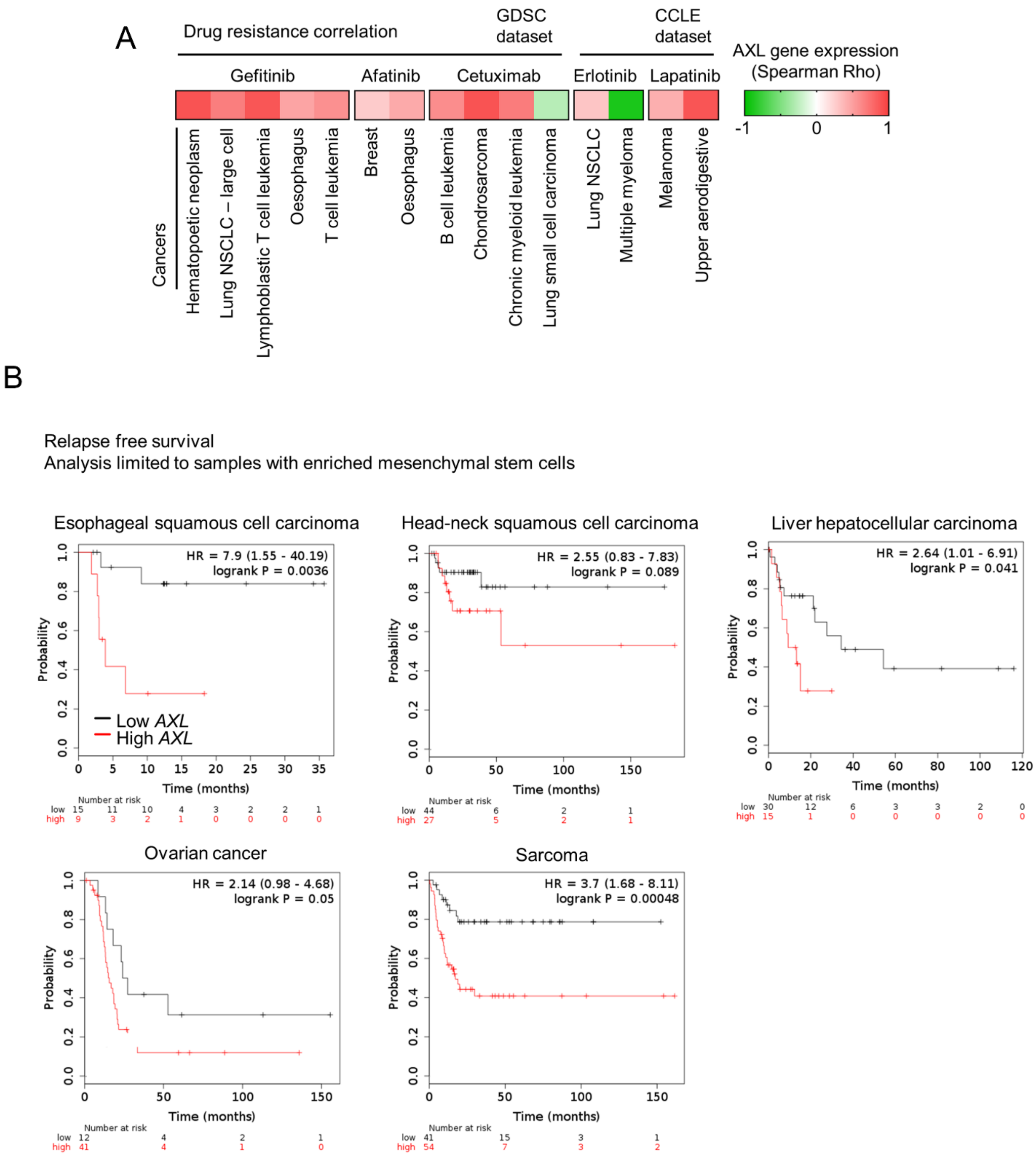


**Supplementary Fig. S8. Association between AXL and therapy failure.**
(**a**) *In silico* prediction of drug efficacy correlated with AXL expression in GDSC and CCLE data sets. Red and green represent positive and negative Spearman's rank correlation coefficients, respectively, between AXL expression and IC50 of indicated EGFR-TKIs. GraphPad Prism 7.01 was used to generate the plot.
(**b**) Kaplan-Meier plots of relapse free survival of cancer patients of indicated types. Patient survival data were stratified by AXL expression (low or high) in their primary tumors limited to samples with enriched mesenchymal stem cells. Risk number of patients stratified per AXL level is indicated. P values were calculated using a log rank test.

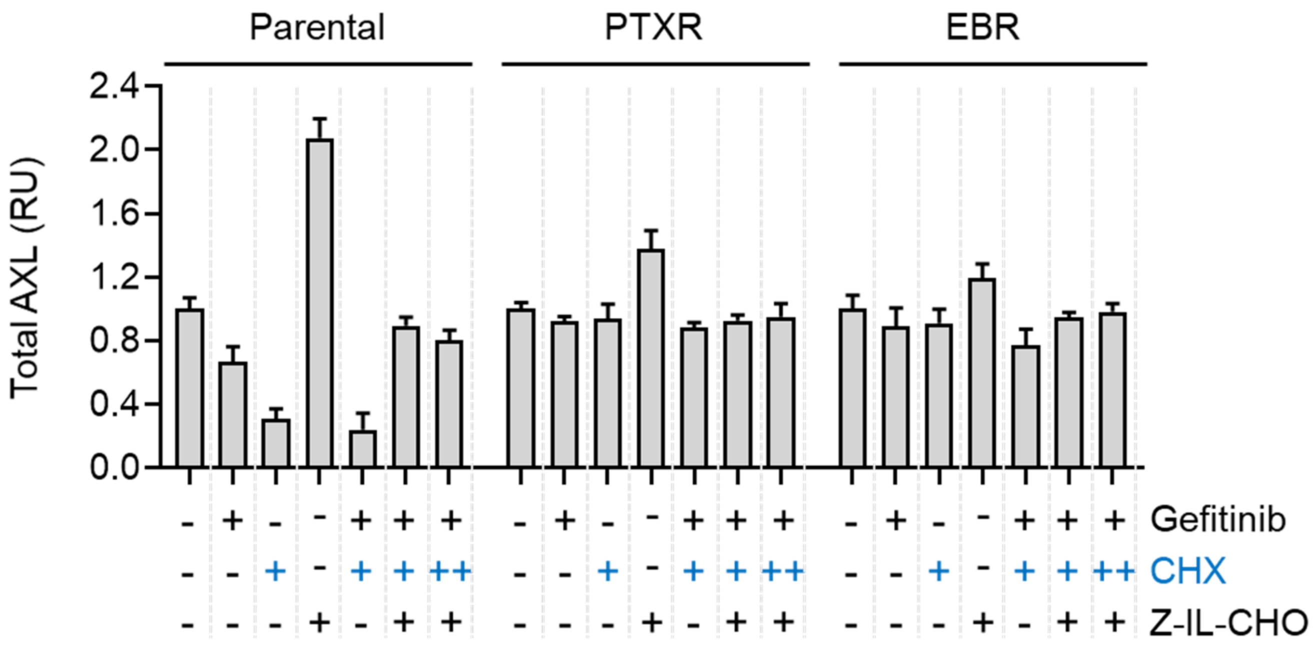


**Supplementary Fig. S9. Gamma-secretase inhibitor Z-IL-CHO effect on AXL.**
Quantification of total AXL in A549-derived parental, PTXR, and EBR cells upon treatment with or without 5 μM gefitinib and with or without 800 nM Z-IL-CHO for 24 h followed by treatment with or without 25 μg/mL CHX for 8 (+) or 12 h (++) (mean±SD of two biological replicates). All measurements are significantly different (*P*<0.05, Student t test) compared with control. GraphPad Prism 7.01 was used to generate the plot.

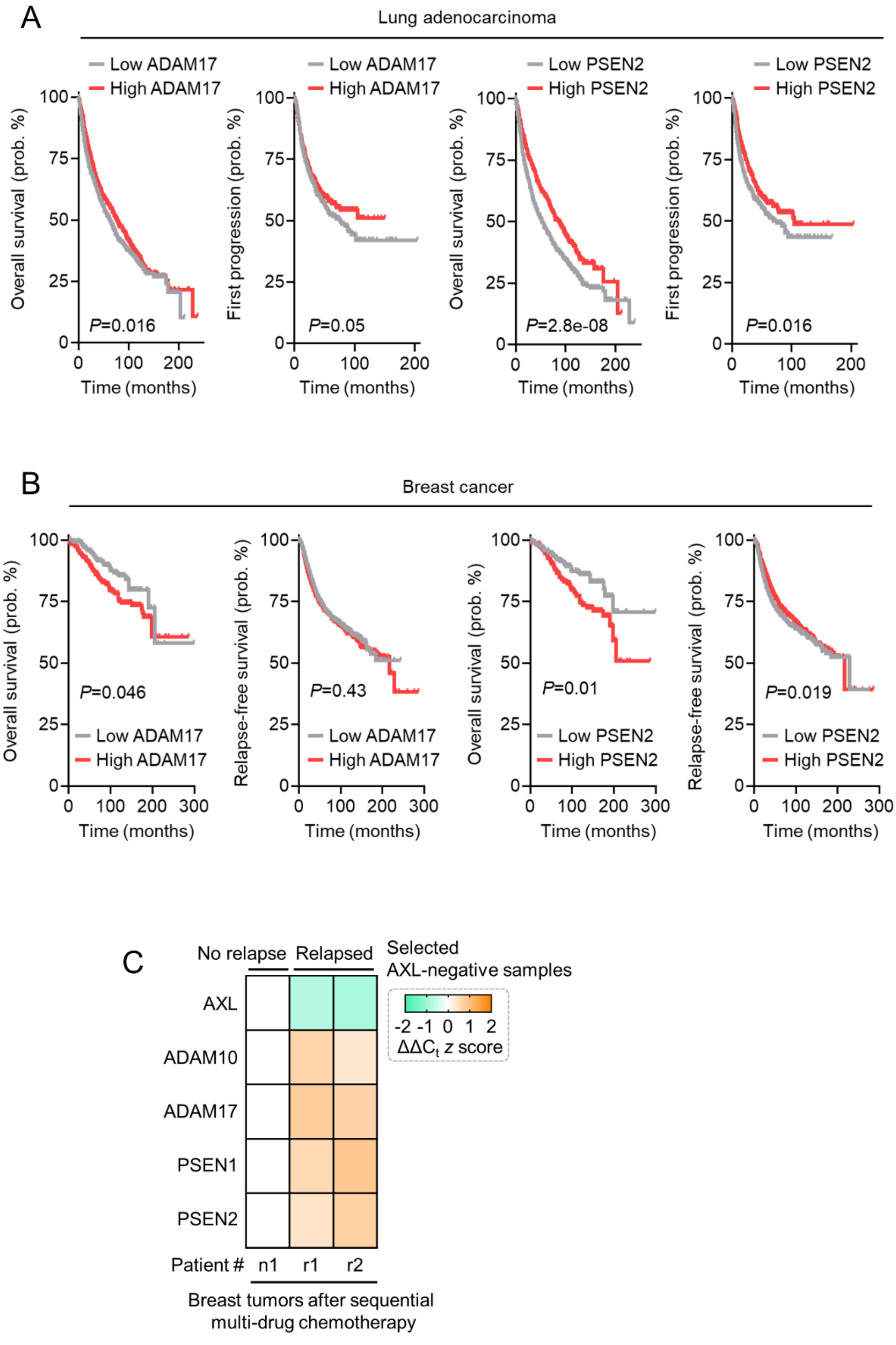


**Supplementary Fig. S10. PS-RIP expression and patient survival.**
(**a**) Kaplan-Meier plots of overall and first progression survival of lung cancer patients. Patient survival data were stratified by ADAM17 or PSEN2 expression (low or high) in their primary tumors. P values were calculated using a log rank test.
(**b**) Kaplan-Meier plots of overall (systematically untreated cohort) and relapse free survival (systemic therapy cohort) of breast cancer patients. Patient survival data were stratified by ADAM17 or PSEN2 expression (low or high) in their primary tumors. P values were calculated using a log rank test.
(**c**) qRT-PCR analysis of AXL and PS-RIP marker expression in FFPE tumor tissue sections from breast cancer patients who underwent sequential multi-drug chemotherapy. Log-transformed gene expression values are relative to the sample with the lowest AXL expression and were normalized to GAPDH levels (mean±SD of three biological replicates). GraphPad Prism 7.01 was used to generate all plots.

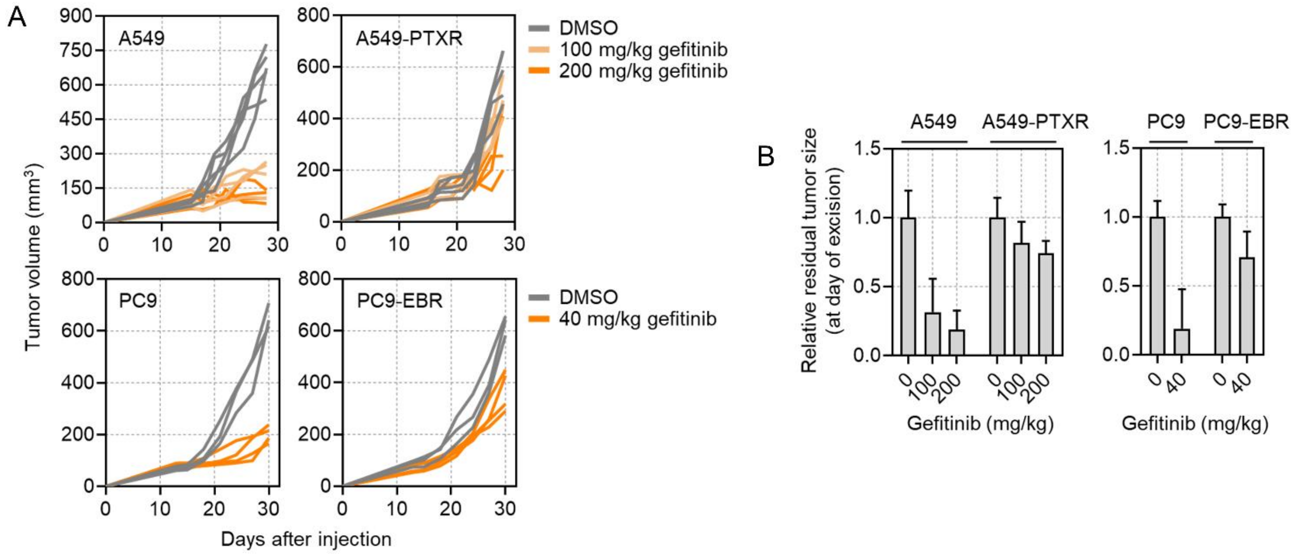


**Supplementary Fig. S11. Residual tumor characterization in xenograft models.**

(**a**) Tumor growth of indicated A549- and PC9-derived cells upon subcutaneous injection into flanks of nude mice. Indicated dose of gefitinib was orally administered once daily for 14 days (A549 xenografts) or 12 days (PC9 xenografts). The control group was treated with an equal volume of vehicle. Tumor volume was measured and monitored using a digital caliper every 2~3 days and indicated time points were selected for plotting (n=4~5 per group, two cohorts per cell line).

(**b**) Relative mass of tumors at the end of gefitinib treatment as defined in Fig. 4i. Tumor mass was measured using a digital weighing scale. GraphPad Prism 7.01 was used to generate all plots.

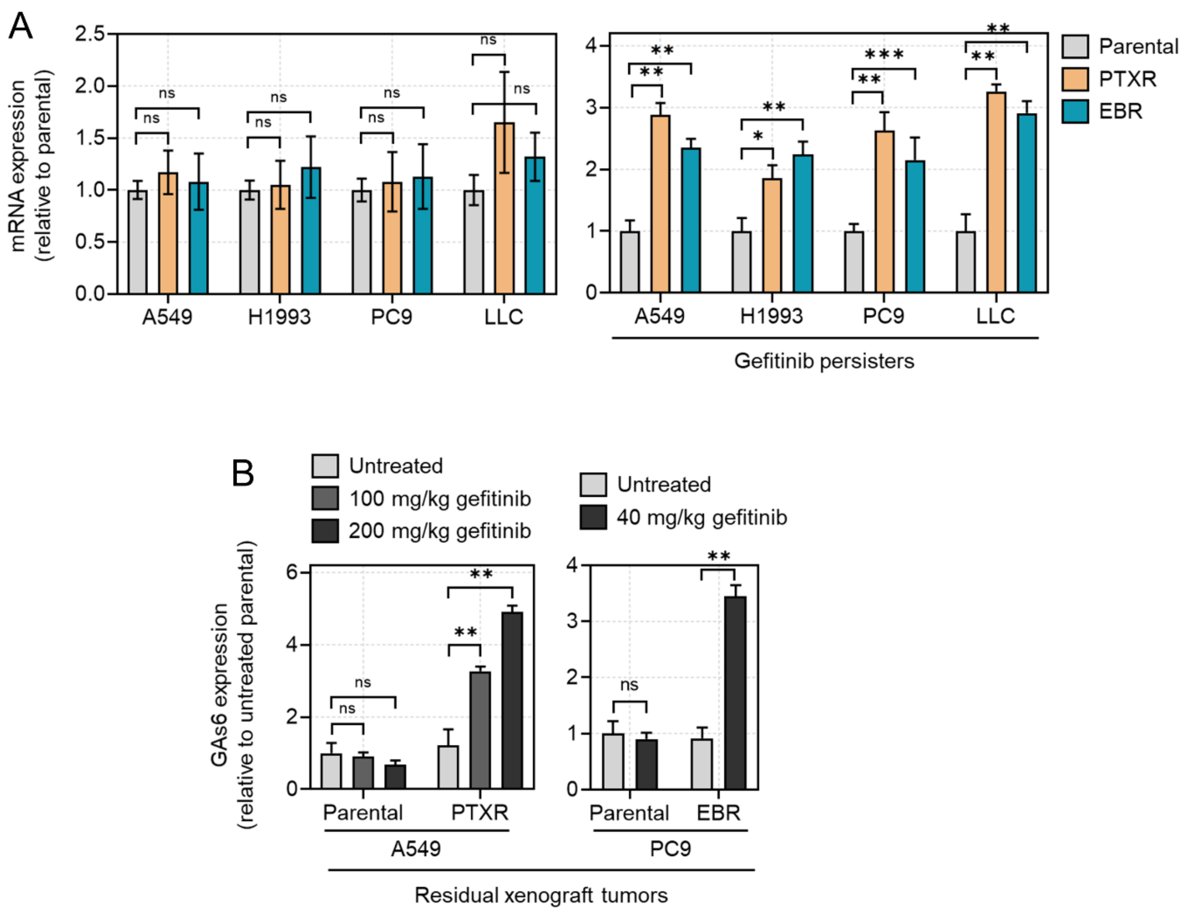


**Supplementary Fig. S12. Analysis of GAS6 expression.**
(**a**) qRT-PCR analysis of expression of GAS6 in indicated cell lines and GPs. Values are relative to parental and were normalized to GAPDH levels (mean±SD of two biological replicates; **P*<0.05, ***P*<0.01, ****P*<0.005, NS=not significant, Student's t test).
(**b**) qRT-PCR analysis of expression of GAS6 in indicated residual tumors detailed in Supplementary Fig. S11. Values are relative to parental and were normalized to GAPDH levels (mean±SD of two biological replicates; **P*<0.05, ***P*<0.01, ****P*<0.005, NS=not significant, Student's t test). GraphPad Prism 7.01 was used to generate all plots.

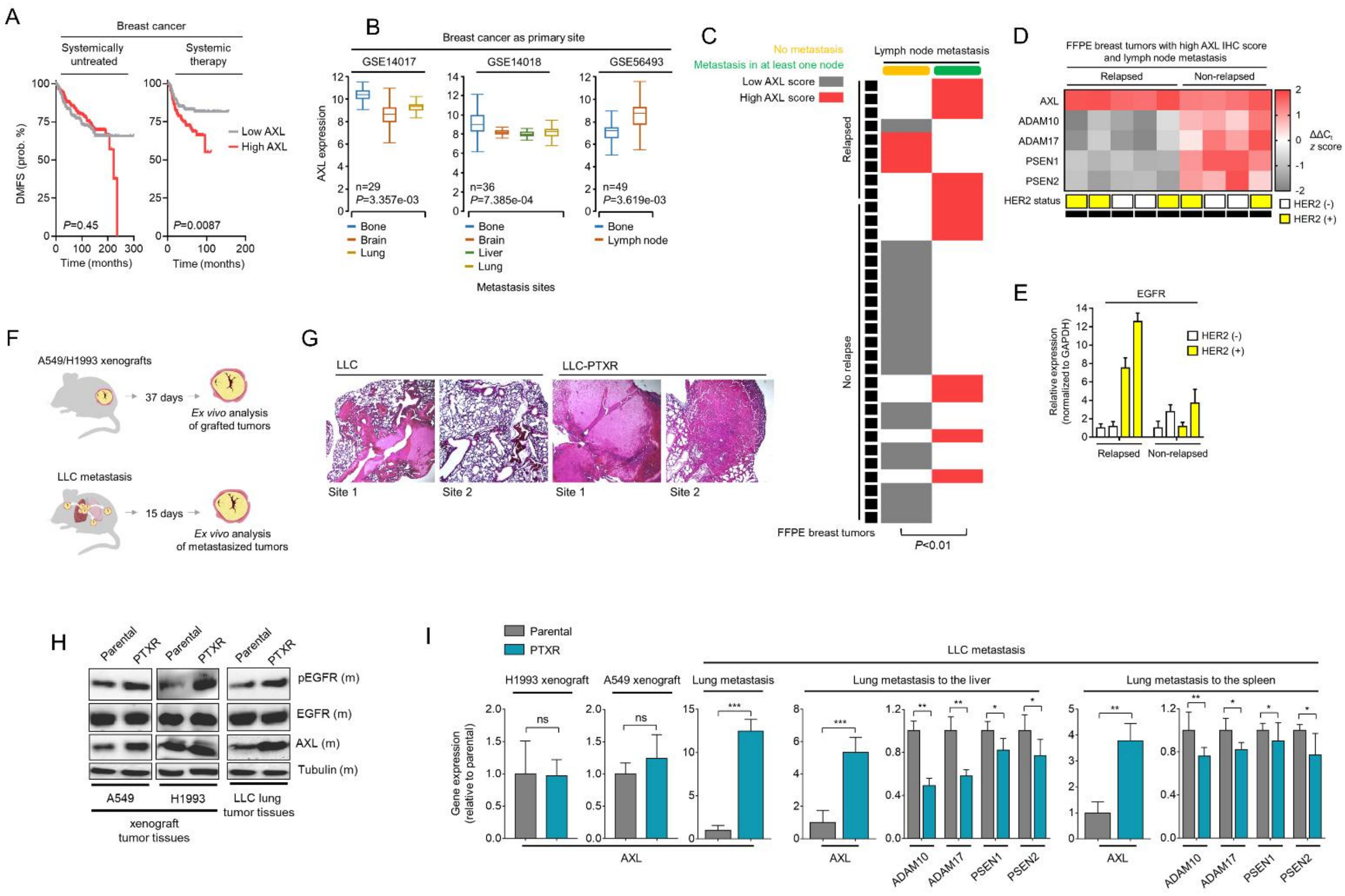

**Supplementary Fig. S13. AXL expression and stability are implicated in relapse and metastasis.**
(**a**) Kaplan-Meier plots of distant metastasis free survival (DMFS) of breast cancer patients, categorized in cohort groups of systemically untreated (patients n = 1015) and chemotherapy-treated patients (patients n = 1543). Patient survival data were stratified by AXL expression (low or high) in their primary tumors. P values were calculated using a log rank test.
(**b**) Correlation analysis of AXL expression and breast metastasis on different distant sites. Data were queried through the HCMD (http://hcmdb.i-sanger.com; see Methods).
(**c**) Association of IHC AXL positivity with lymph node metastasis of patients as in Fig. 4g (total of 33 unique patient cases).
(**d**) qRT-PCR analysis of AXL and PS-RIP marker expression in FFPE tumor tissue sections from breast cancer patients who underwent sequential multi-drug chemotherapy. Selected tissues stained high IHC positivity to AXL. HER2 positivity was identified based on a PCR test. Log-transformed gene expression values are relative to the sample with the lowest AXL expression and were normalized to GAPDH levels (mean±SD of three biological replicates).
(**e**) qRT-PCR analysis of EGFR expression in indicated selected samples from d. Values are relative to the sample with the lowest EGFR expression and were normalized to GAPDH levels (mean±SD of three biological replicates).
(**f**) Schematic of xenograft and metastasis experiments.
(**g**) H&E staining of two different sites of primary lung tumor metastasis from our LLC1-induced metastasis model.
(**h**) Western blot analysis of indicated proteins in xenograft tumor tissues derived from parental or PTXR cells (A549 and H1993) and in primary lung tumor metastasis derived from parental or PTXR cells (LLC1). Representative of two independent experiments. 20 µg of total tumor lysates were loaded per lane. Samples from the same cell line were run on the same gel. Paired samples are highlighted in black frame.
(**i**) qRT-PCR analysis of AXL and PS-RIP marker expression in indicated xenograft tumor tissues and metastasized tumor tissues. Values are relative to parental and were normalized to GAPDH levels (mean±SD of three biological replicates; **P*<0.05, ***P*<0.01, ****P*<0.005, NS=not significant, Student's t test). GraphPad Prism 7.01 was used to generate all plots.

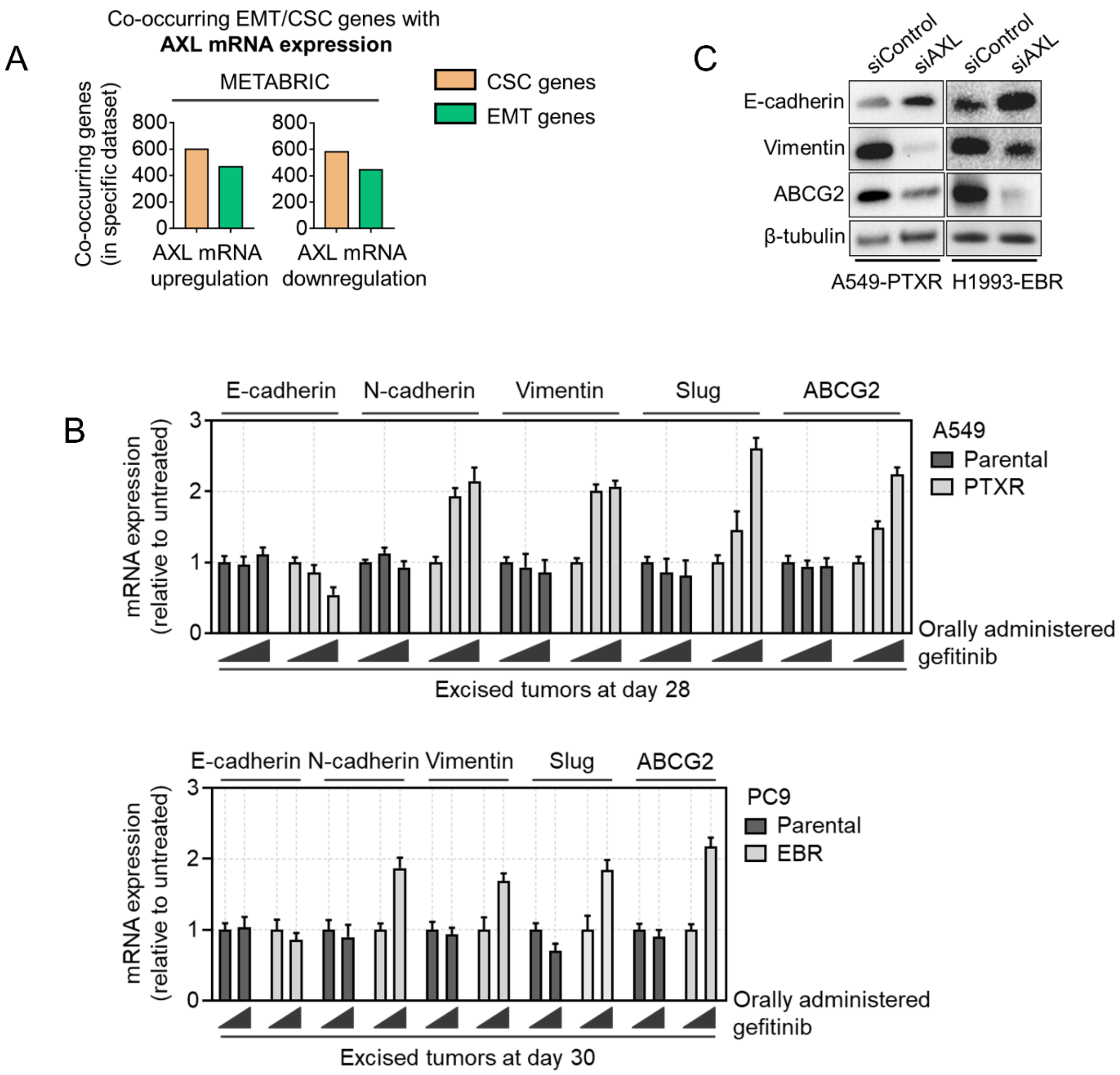


**Supplementary Fig. S14. EMT and CSC gene expression profiles.**

(**a**) Co-occurrence analysis of AXL mRNA alterations with EMT (out of 342 genes) and CSC (out of 1782 genes) associated genes, respectively, analyzed using the METABRIC data set.

(**b**) qRT-PCR analysis of expression of EMT and CSC markers in xenograft tumors derived from parental and PTXR cells excised at day 28 (A549-derived xenografts) or day 30 (PC9-derived xenografts) following 40, 100, or 200 mg/kg gefitinib therapy. Values are relative to parental untreated and were normalized to GAPDH levels (mean±SD of four biological replicates).

(**c**) Western blot analysis of indicated proteins in GPs derived from A549-PTXR or H1993-EBR cells upon AXL RNAi. Representative of two independent experiments. 45 μg of total cell lysates were loaded per lane. Samples from the same cell line were run on the same gel highlighted in black frame. GraphPad Prism 7.01 was used to generate all plots.

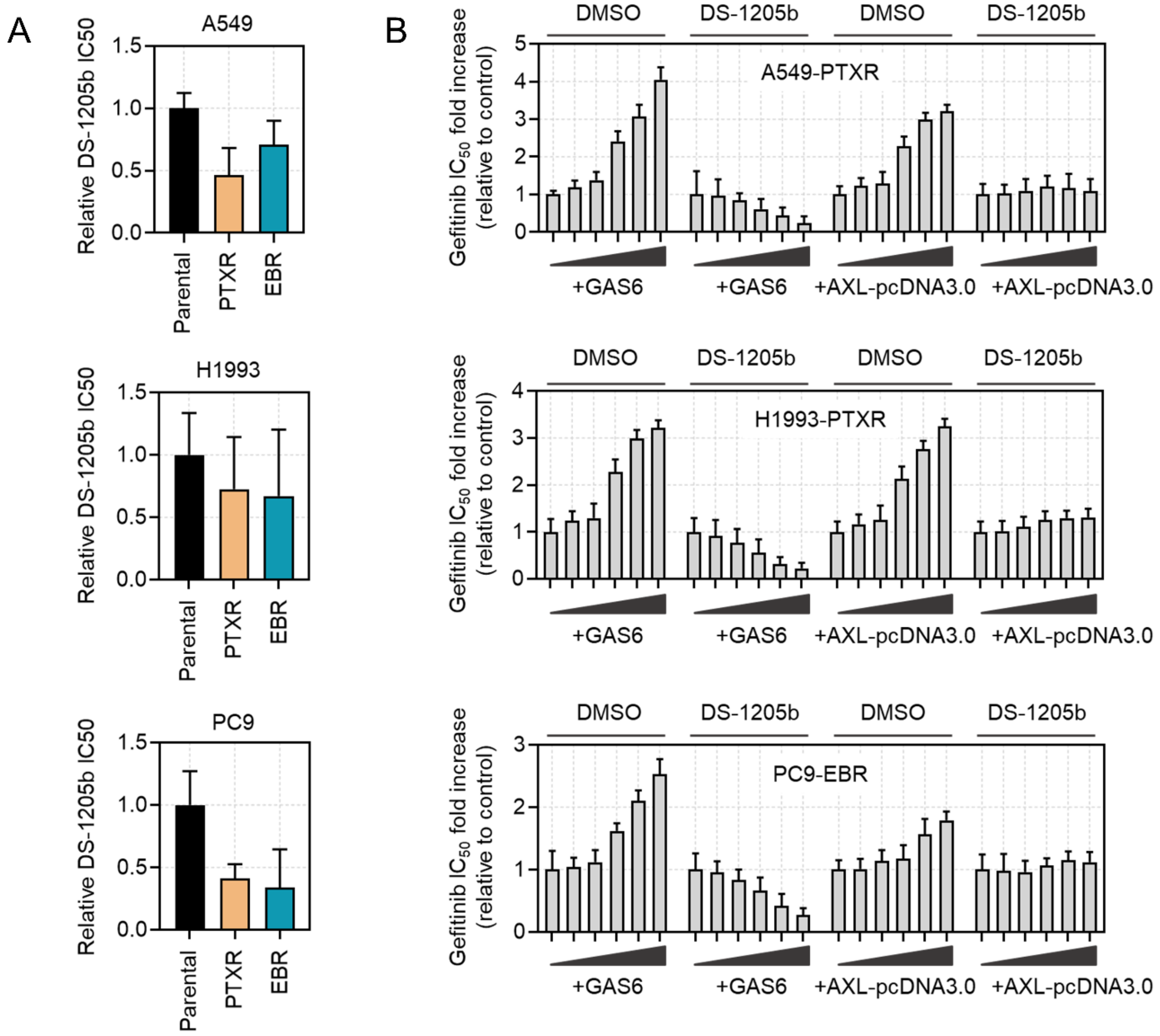


**Supplementary Fig. S15. Collateral gefitinib resistance in CTD-resistant cells is driven by an AXL-dependent mechanism.**

(**a**) Characterization of sensitivity to the AXL-selective inhibitor DS-1205b in indicated cells derived from A549, H1993, or PC9. Sensitivity was assessed based on IC50 values. Cells were treated with or without DS-1205b for 72 h with a concentration dilution series and were assayed for SRB. Values are relative to indicated control (mean±SD of three biological replicates).

(**b**) Characterization of gefitinib resistance in PTXR or EBR cells derived from A549, H1993, or PC9. Resistance was assessed based on IC50 fold values. Cells were transfected with or without increasing concentrations of AXL-pcDNA3.0 for 48 h or treated with or without increasing concentrations of AXL's ligand GAS6 (0.625, 1.25, 2.5, 5, 10 nM). As a co-treatment, cells were treated with or without 50 nM DS-1205b. Cells were treated with or without gefitinib for 72 h with a concentration dilution series and were assayed for SRB. Values are relative to indicated control (mean±SD of three biological replicates). GraphPad Prism 7.01 was used to generate all plots.

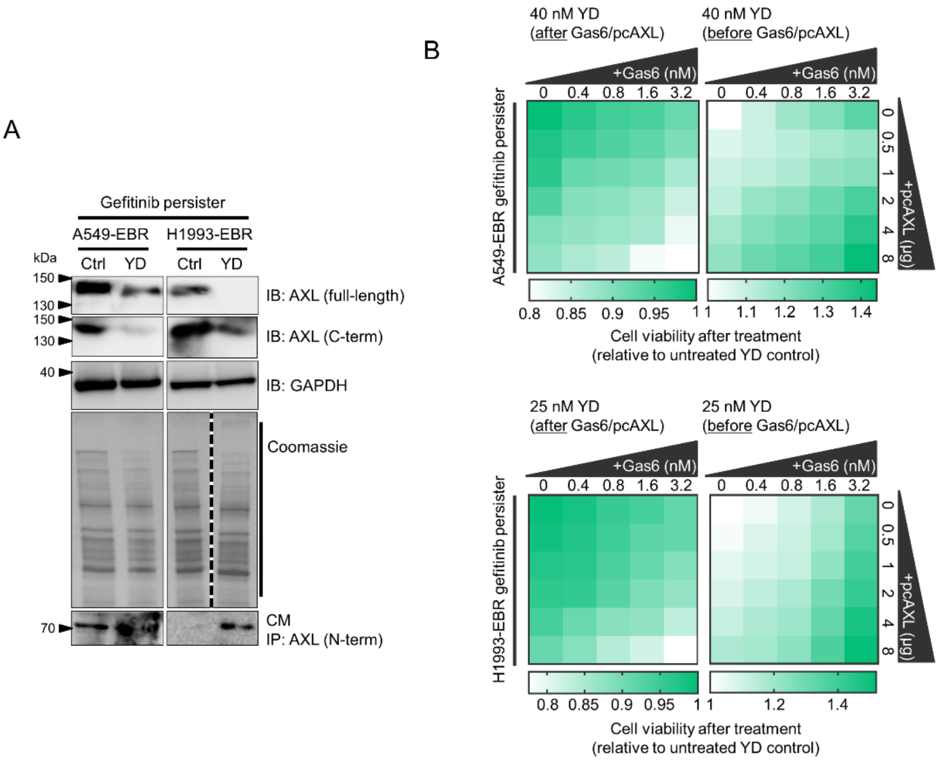


**Supplementary Fig. S16. YD inhibits resistance by targeting AXL and promoting receptor shredding.** (**a**) Characterization of YD-induced AXL suppression in indicated EBR-derived GPs. Cells were treated with or without 30 nM YD (in A549-derived GPs) and 45 nM YD (in H1993-derived GPs) for 48 h. Conditioned culture media (CM) were harvested, immunoprecipitated with antibody against N-terminal AXL, and immunoblotted using anti-N-terminal AXL. Cells were also subjected for immunoblotting with anti-C-terminal AXL. GAPDH was used as a loading control. In-gel proteins were visualized with Coomassie blue. Representative of two independent experiments. 40 µg of total cell lysates were loaded per lane for immunoblotting while 50 µg of pulled-down proteins were loaded for immunoprecipitation. Samples from the same cell line were run on the same gel. Paired samples are highlighted in black frame. Broken line indicates cropped gel.

(**b**) Cell viability of indicated EBR-derived GPs upon treatment with 30 nM YD (in A549-derived GPs) and 45 nM YD (in H1993-derived GPs) for 48 h. Prior to YD treatment, cells were treated or transfected with or without the indicated concentrations of Gas6 ligand or pcDNA3.0-AXL plasmid for 24 h. Values are relative to untreated control. Representative of three independent experiments. Python (seaborn) was used to generate the plot.

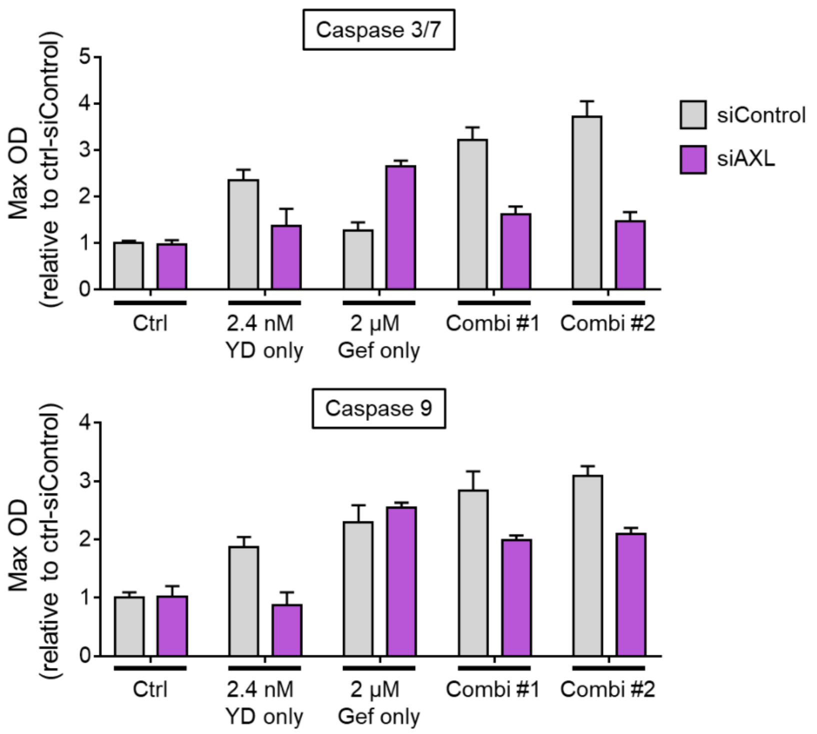


**Supplementary Fig. S17. Apoptosis induced through synergism between YD and gefitinib.**
Caspase 3/7 DEVDase and caspase 9 activities in A549-PTXR-derived GPs upon AXL RNAi. Cells were treated with indicated drugs alone or in combination. Cells were then treated with or without gefitinib for 72 h with a concentration dilution series and were assayed for SRB. Resistance was assessed based on drug IC50 values. Values are relative to siControl (mean±SD of three biological replicates). GraphPad Prism 7.01 was used to generate the plot.

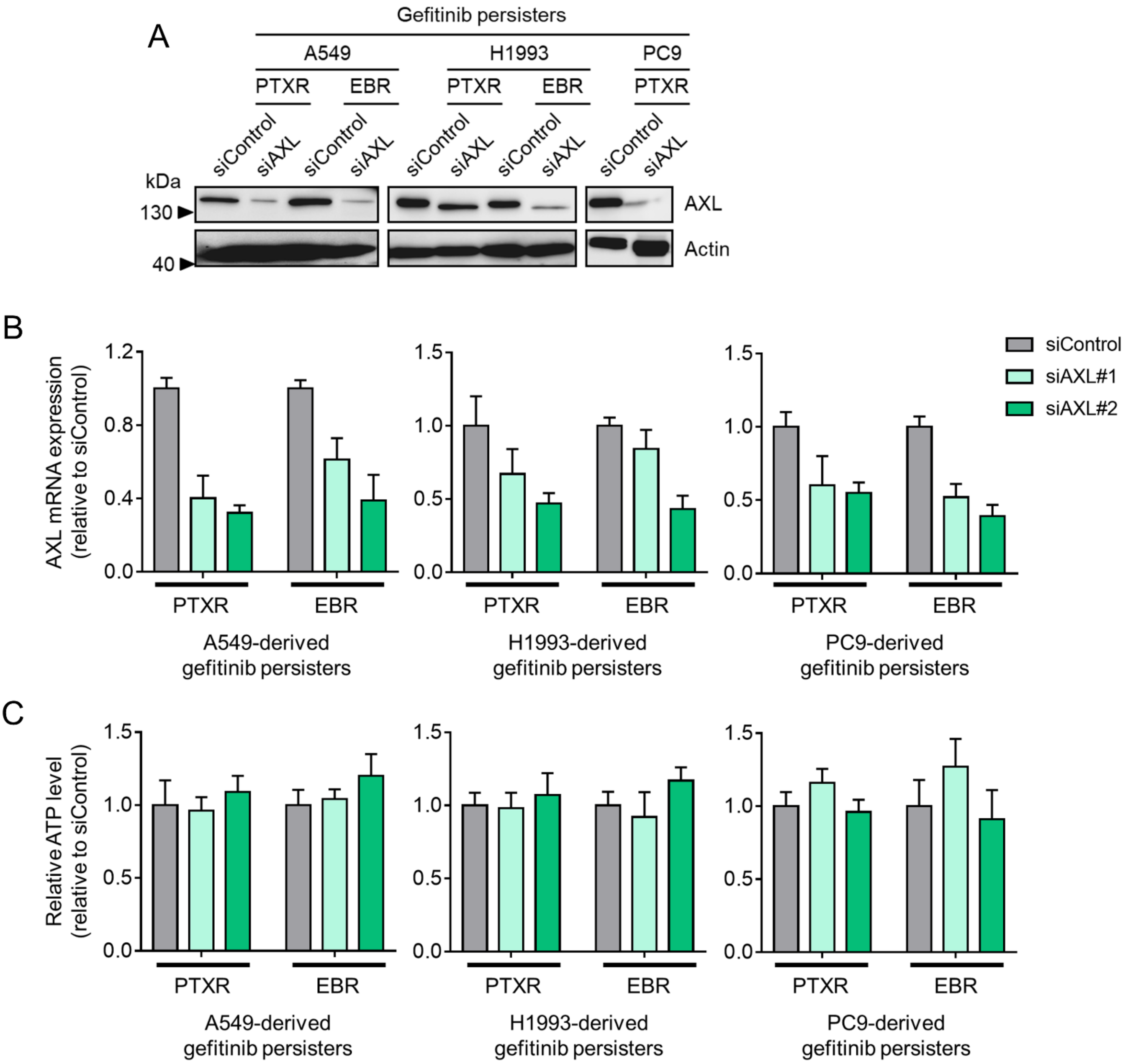


**Supplementary Fig. S18. AXL RNAi in GPs.**

(**a**) Western blot analysis of AXL upon AXL RNAi for 48 h in indicated GPs derived from A549, H1993, and PC9 cells. Actin was used as a loading control. Representative of two independent experiments. 40 μg of total cell lysates were loaded per lane. Samples from the same cell line were run on the same gel highlighted in black frame.

(**b**) qPCR analysis of AXL expression upon AXL RNAi for 48 h in indicated GPs derived from A549, H1993, and PC9 cells. Values are relative to siControl and were normalized to GAPDH levels (mean±SD of three biological replicates).

(**c**) ATP levels of indicated GPs upon AXL RNAi for 48 h as in a. Values are relative to siControl (mean±SD of three biological replicates). GraphPad Prism 7.01 was used to generate all plots.

**Supplementary Table S1**. Primer sequences for qRT-PCR.

| Target gene | Forward (5'→3') | Reverse (5'→3') |
|---|---|---|
| AXL (h) | CGTAACCTCCACCTGGTCTC | TCCCATCGTCTGACAGCA |
| ADAM10 (h) | ATATTACGGAACACGAGAAGCTG | TCAATCGCTTTAACATGACTGG |
| ADAM17 (h) | CCTTTCTGCGAGAGGGAAC | CACCTTGCAGGAGTTGTCAGT |
| PSEN1 (h) | CCTCAACAATGGTGTGGTTG | TTGTGACTCCCTTTCTGTGCT |
| PSEN2 (h) | CCGGGATTCAGACCTCTCT | GGCCATGAATGTGAGCATAG |
| AXL (m) | GGAACCCAGGGAATATCACAGG | AGTTCTAGGATCTGTCCATCTCG |
| GAS6 (h) | GTAGCTTCCACTGTTCCT | GCGCACTCGTCTATGTCTT |
| GAS6 (m) | CCGTGATTAGACTACGCTTC | AGTTGAGCCTGTAGGTAGCA |
| ADAM10 (m) | GCAACATCTGGGGACAAACT | TTGCACTGGTCACTGTAGCC |
| ADAM17 (m) | AGGATGCTTGGGATGTGAAG | CTGTTTGCTCTGGGAGAACC |
| PSEN1 (m) | (A/G)ACGG(G/T)CAGCT(A/C)ATCTACAC | GAT(A/G)AA(C/T)ACCAGGGCCATGAG |
| PSEN2 (m) | CTCAGCAAGCAAGCGTCTCTTC | TCCCAGCAGTCACTGCAGAAAT |
| CDH1 (E-cadherin) | GAACAGCACGTACACAGCCCT | GCAGAACTGTCCCTGTCCCAG |
| CDH2 (N-cadherin) | GACGGTTCGCCATCCAGAC | TCGATTGGTTTGACCACGG |
| VIM (Vimentin) | TACAGGAAGCTGCTGGAAGG | ACCAGAGGGAGTGAATCCAG |
| FN1 (Fibronectin) | GGAAAGTGTCCCTATCTCTGATACC | AATGTTGGTGAATCGCAGGT |
| SNAI2 (Slug) | TGGTTGCTTCAAGGACACAT | GTTGCAGTGAGGGCAAGAA |
| CTNNB1 (β-catenin) | GTGCAATTCCTGAGCTGACA | CTTAAAGATGGCCAGCAAGC |
| NANOG | CCTCCTCCATGGATCTGCTTATTCA | CAGGTCTTCACCTGTTTGTAG |
| OCT4 | ACATCAAAGCTCTGCAGAAAGAACT | CTGAATACCTTCCCAAATAGAACCC |
| LIN28 | TTGTCTTCTACCCTGCCCTCT | GAACAAGGGATGGAGGGTTTT |
| BMI1 | CCAGGGCTTTTCAAAAATGA | CCGATCCAATCTGTTCTGGT |
| ABCG2 | CAGGTCTGTTGGTCAATCTCACA | TCCATATCGTGGAATGCTGAAG |
| SOX2 | AGCTACAGCATGATGCAGGA | GGTCATGGAGTTGTACTGCA |
| CD133 | TGGATGCAGAACTTGACAACGT | ATACCTGCTACGACAGTCGTGGT |
| EGFR | GCACCCAAGCCCATGCCGTGGCTGC | GAAACAAAGAGTAAAGTAGATGATGG |
| GAPDH (h) | CCACATCGCTCAGACACCAT | GGCAACAATATCCACTTTACCAGAGT |
| GAPDH (m) | ACCACAGTCCATGCCATCAC | TCCACCACCCTGTTGCTGTA |

## Raw blotting and stained SDS-PAGE gel images

Author notes:

- Blotting membranes were cut prior to probing with appropriate antibodies.
- Raw images were cropped post-detection in the ChemiDoc imaging system prior to export as TIFF images.
- Levels of exposure were appropriately adjusted using the Image Lab software (Bio-rad; https://www.bio-rad.com/ko-kr/product/image-lab-software?ID=KRE6P5E8Z)
- Blotting and stained SDS-PAGE gel images shown here are raw and unedited (i.e., contrast, brightness).

Figure 4A pt.1

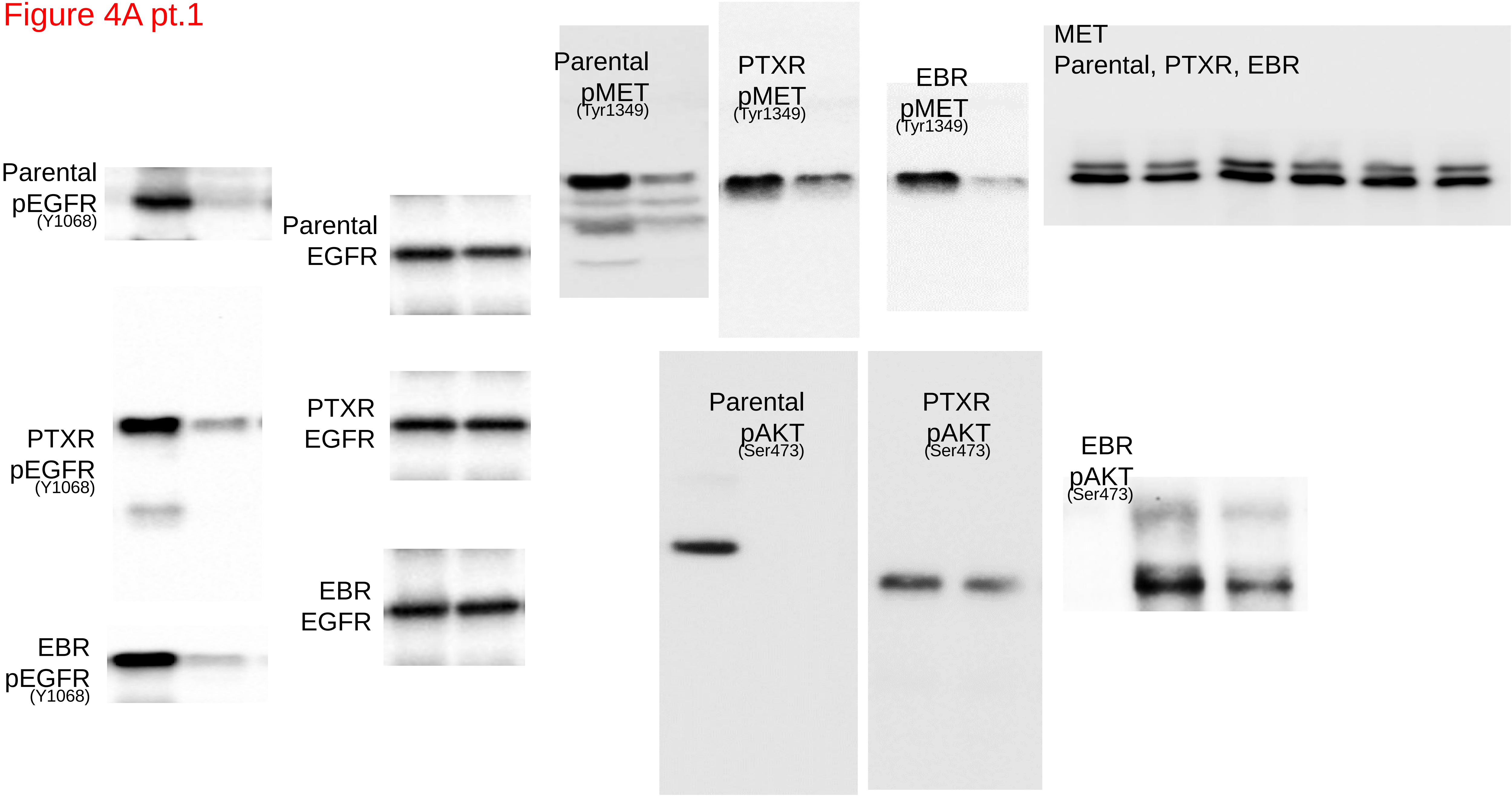

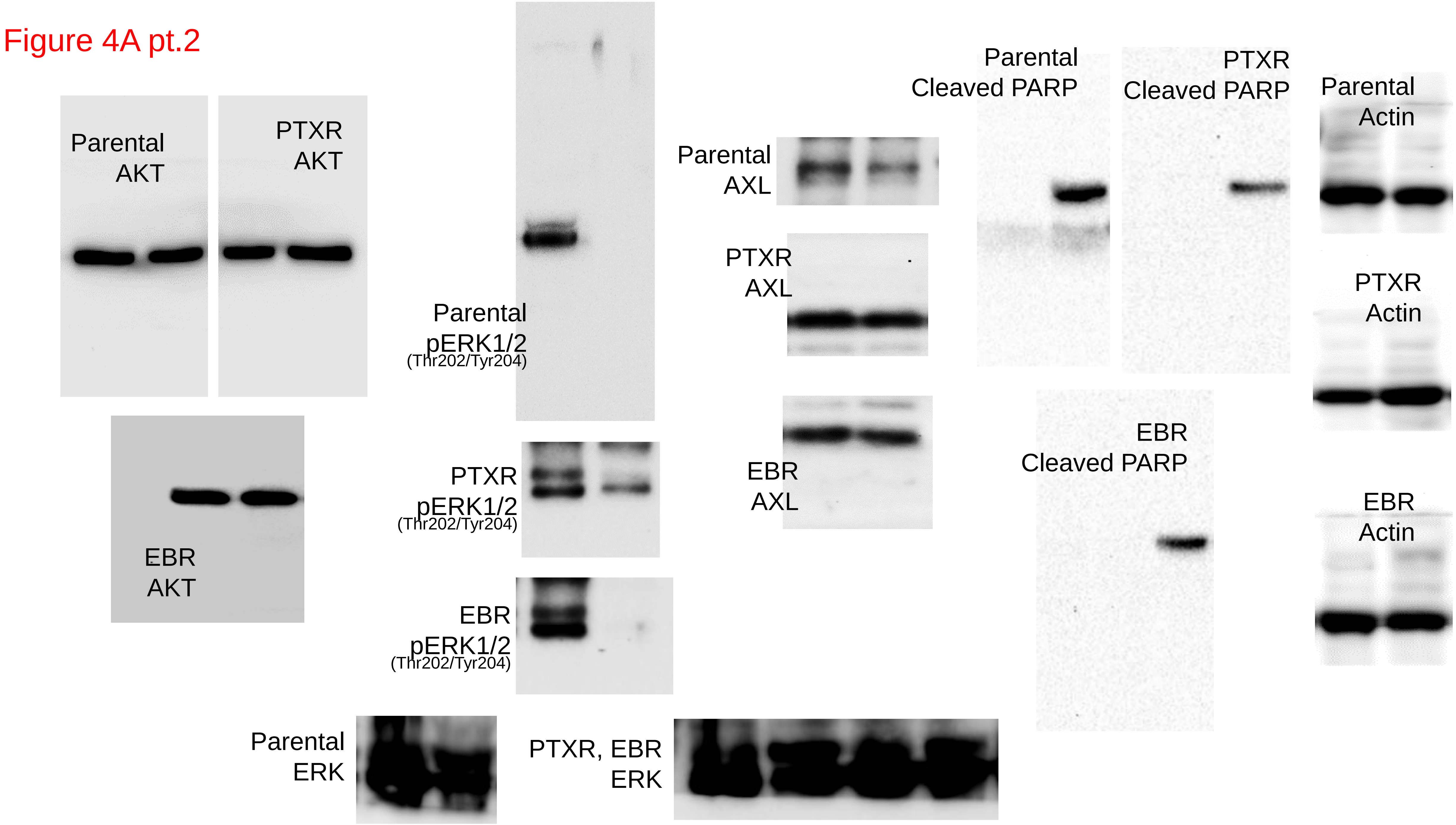
Figure 4A pt.2
Parental
AKT
PTXR
AKT
EBR
AKT
Parental
pERK1/2
(Thr202/Tyr204)
PTXR
pERK1/2
(Thr202/Tyr204)
EBR
pERK1/2
(Thr202/Tyr204)
Parental
ERK
PTXR, EBR
ERK
Parental
AXL
PTXR
AXL
EBR
AXL
Parental
Cleaved PARP
PTXR
Cleaved PARP
EBR
Cleaved PARP
Parental
Actin
PTXR
Actin
EBR
Actin

Figure 4D

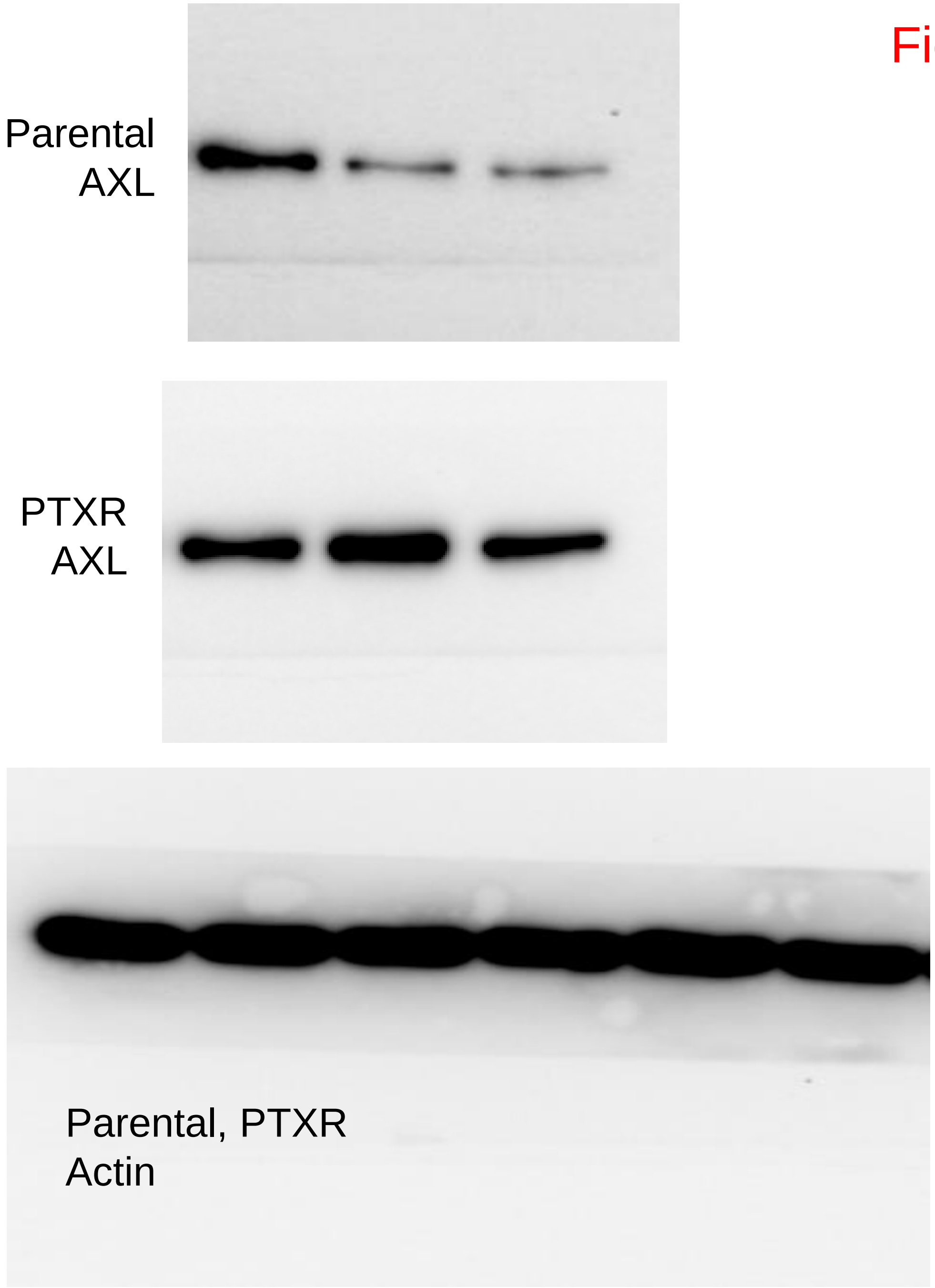


Figure 4E

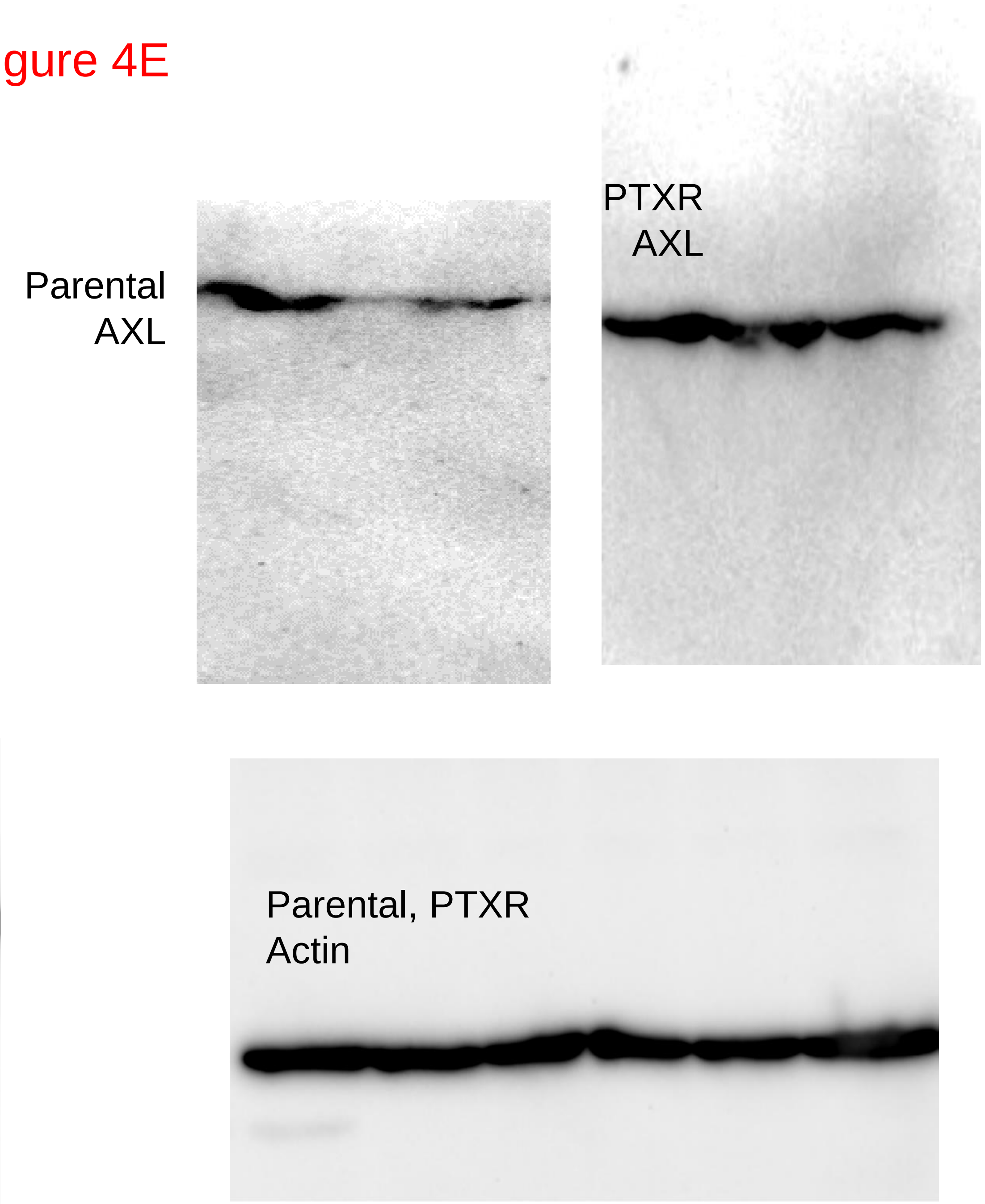

# Figure 5H

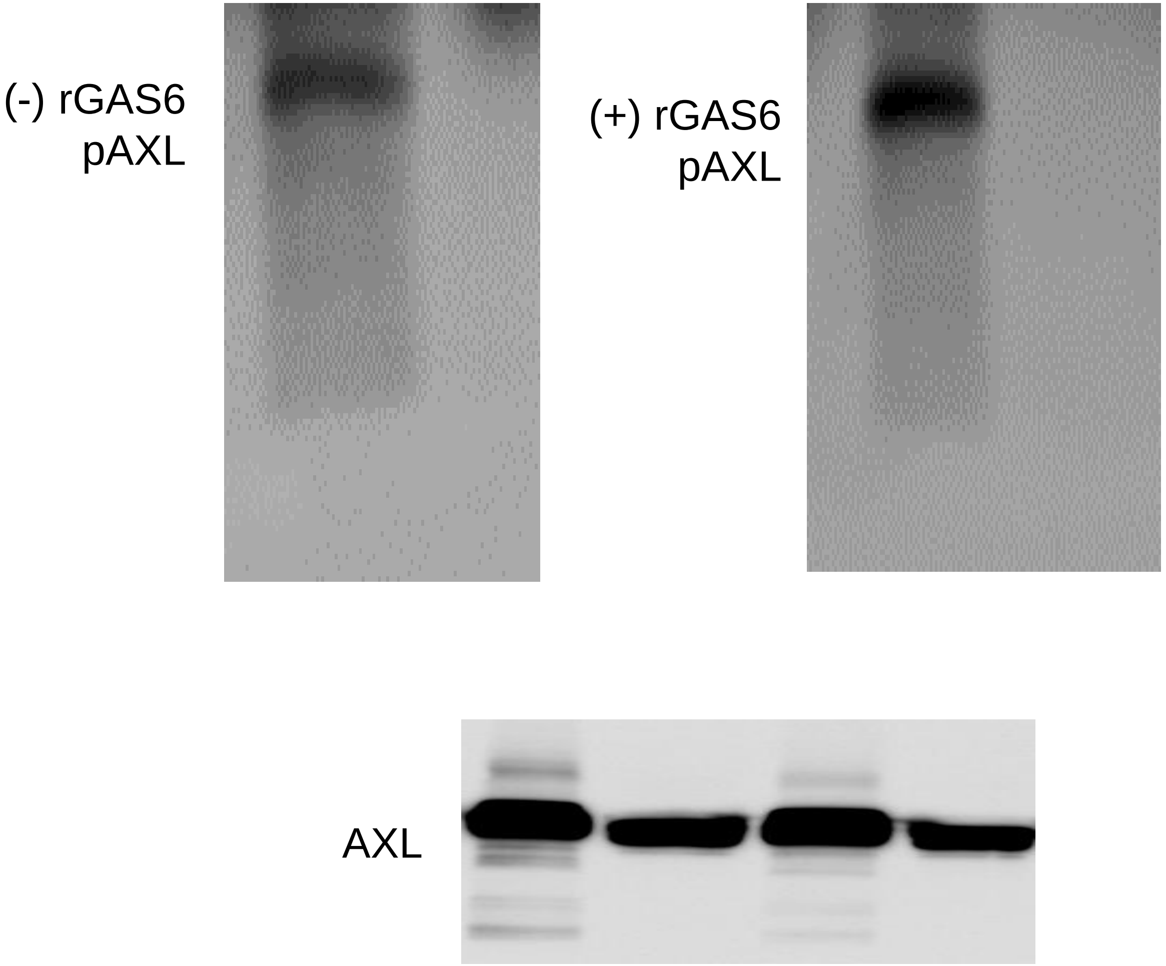

Figure 7D

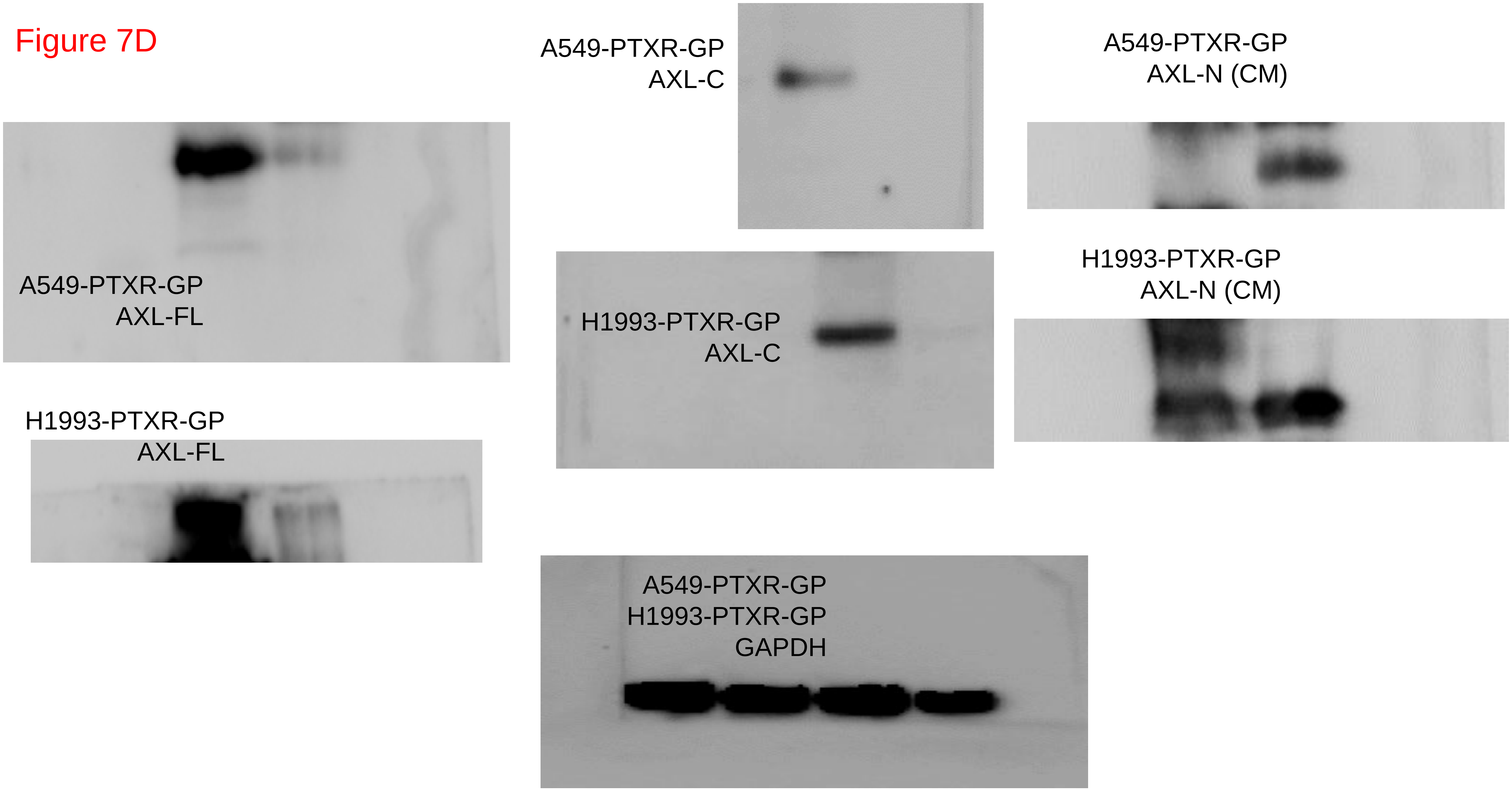
A549-PTXR-GP
AXL-C
A549-PTXR-GP
AXL-N (CM)
A549-PTXR-GP
AXL-FL
H1993-PTXR-GP
AXL-C
H1993-PTXR-GP
AXL-N (CM)
H1993-PTXR-GP
AXL-FL
A549-PTXR-GP
H1993-PTXR-GP
GAPDH

# Figure 7D & Supp. Fig. 16A

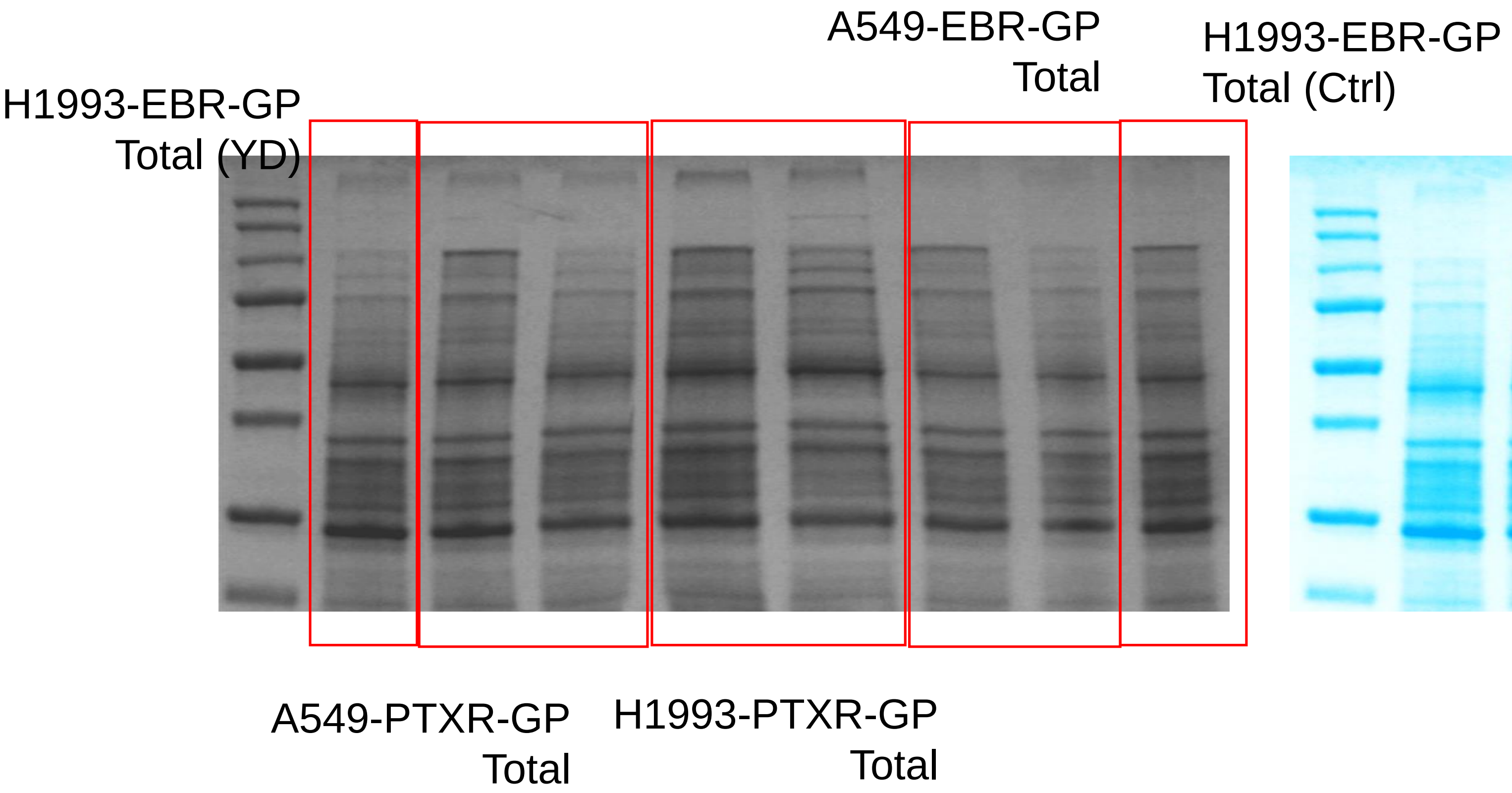


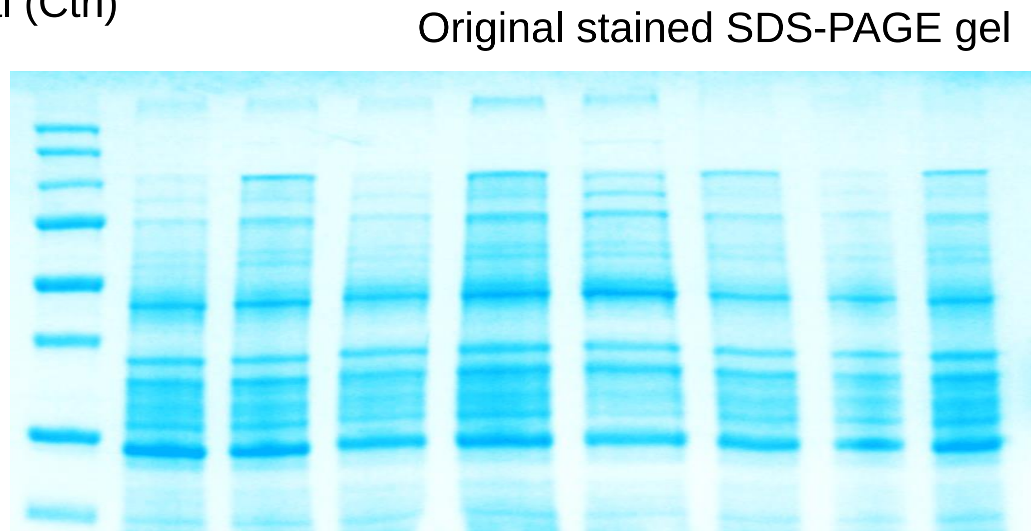

Supp. Fig. 13H

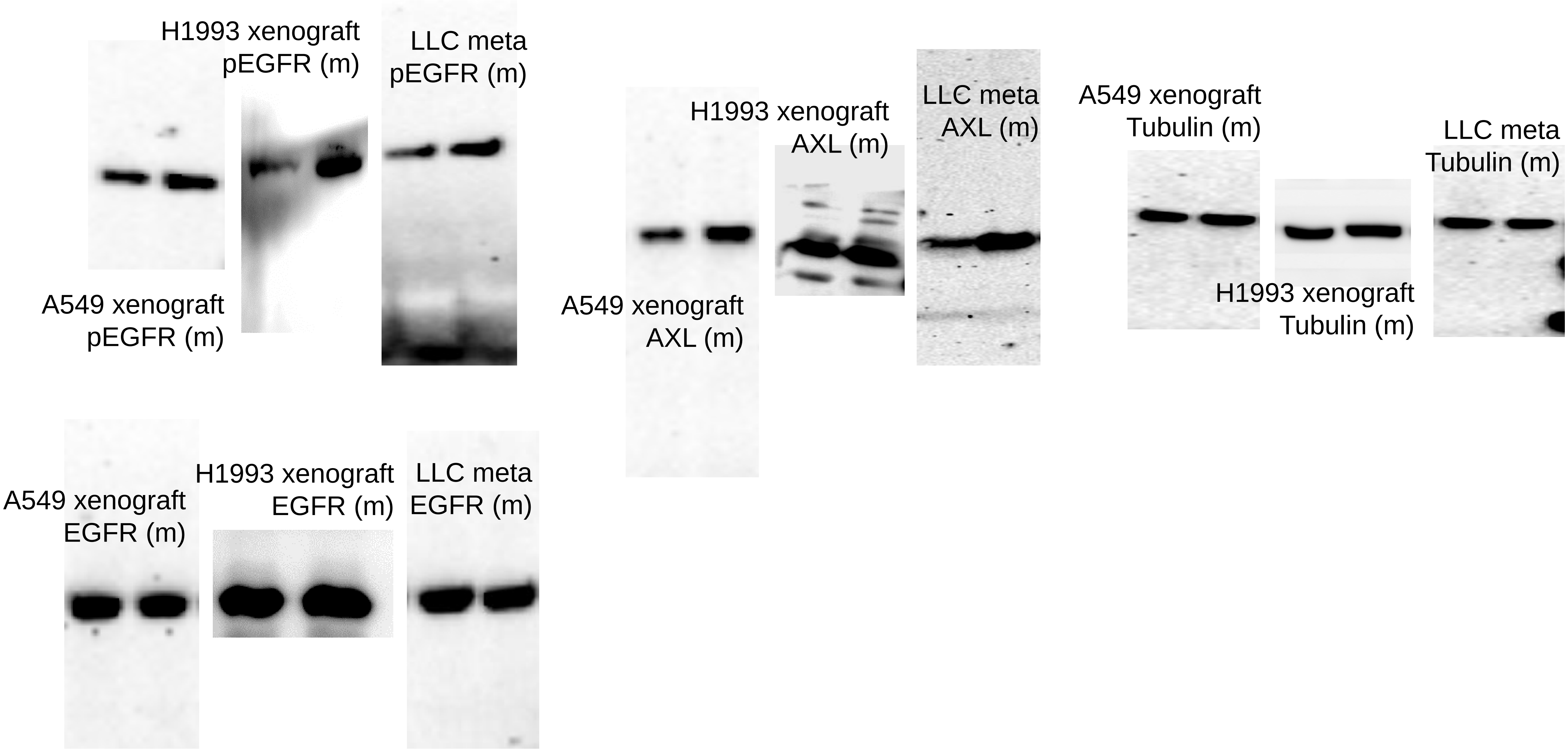
H1993 xenograft pEGFR (m)
LLC meta pEGFR (m)
A549 xenograft pEGFR (m)
H1993 xenograft AXL (m)
LLC meta AXL (m)
A549 xenograft AXL (m)
A549 xenograft Tubulin (m)
LLC meta Tubulin (m)
H1993 xenograft Tubulin (m)
A549 xenograft EGFR (m)
H1993 xenograft EGFR (m)
LLC meta EGFR (m)

Supp. Fig. 14C

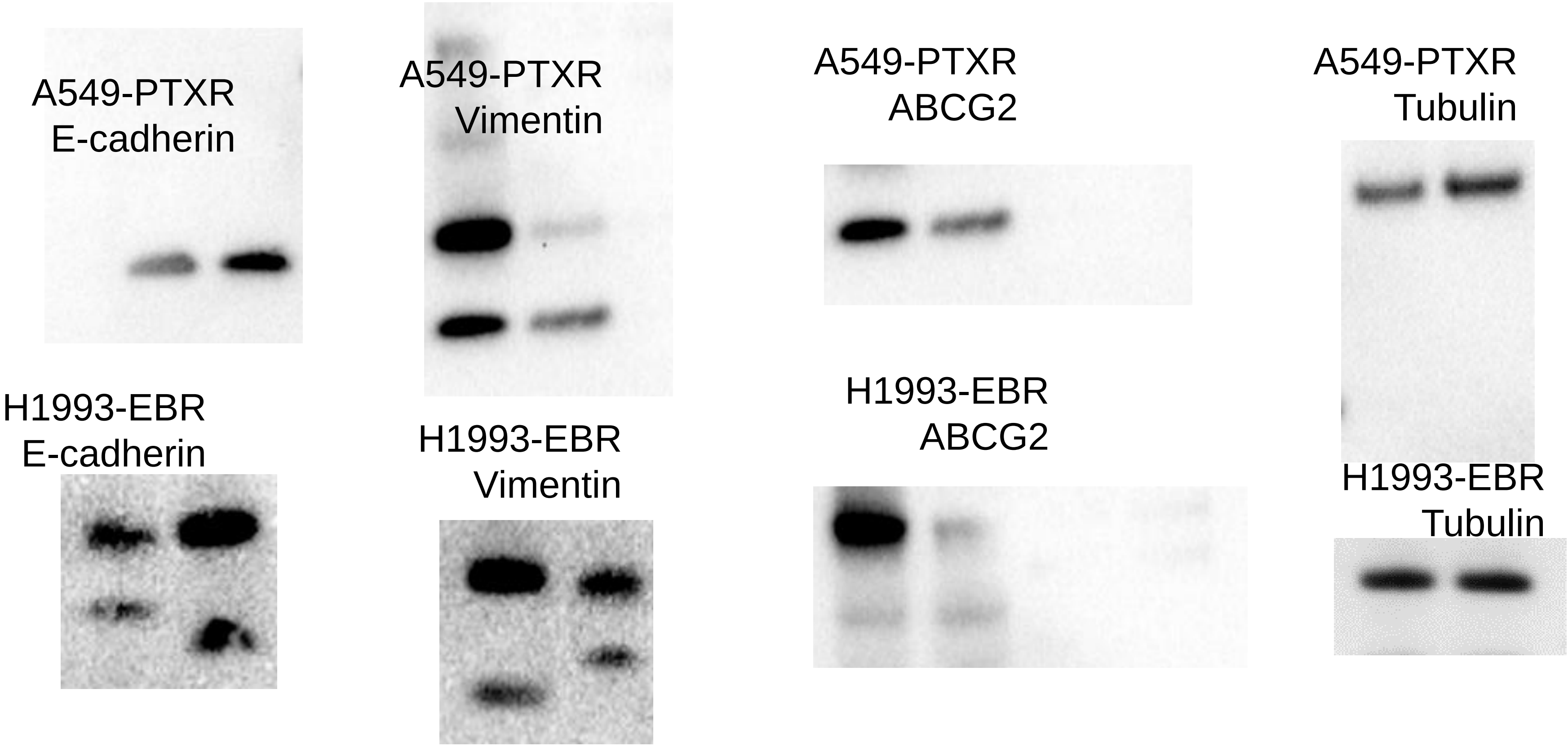
A549-PTXR
E-cadherin
A549-PTXR
Vimentin
A549-PTXR
ABCG2
A549-PTXR
Tubulin
H1993-EBR
E-cadherin
H1993-EBR
Vimentin
H1993-EBR
ABCG2
H1993-EBR
Tubulin

Supp. Fig. 16A

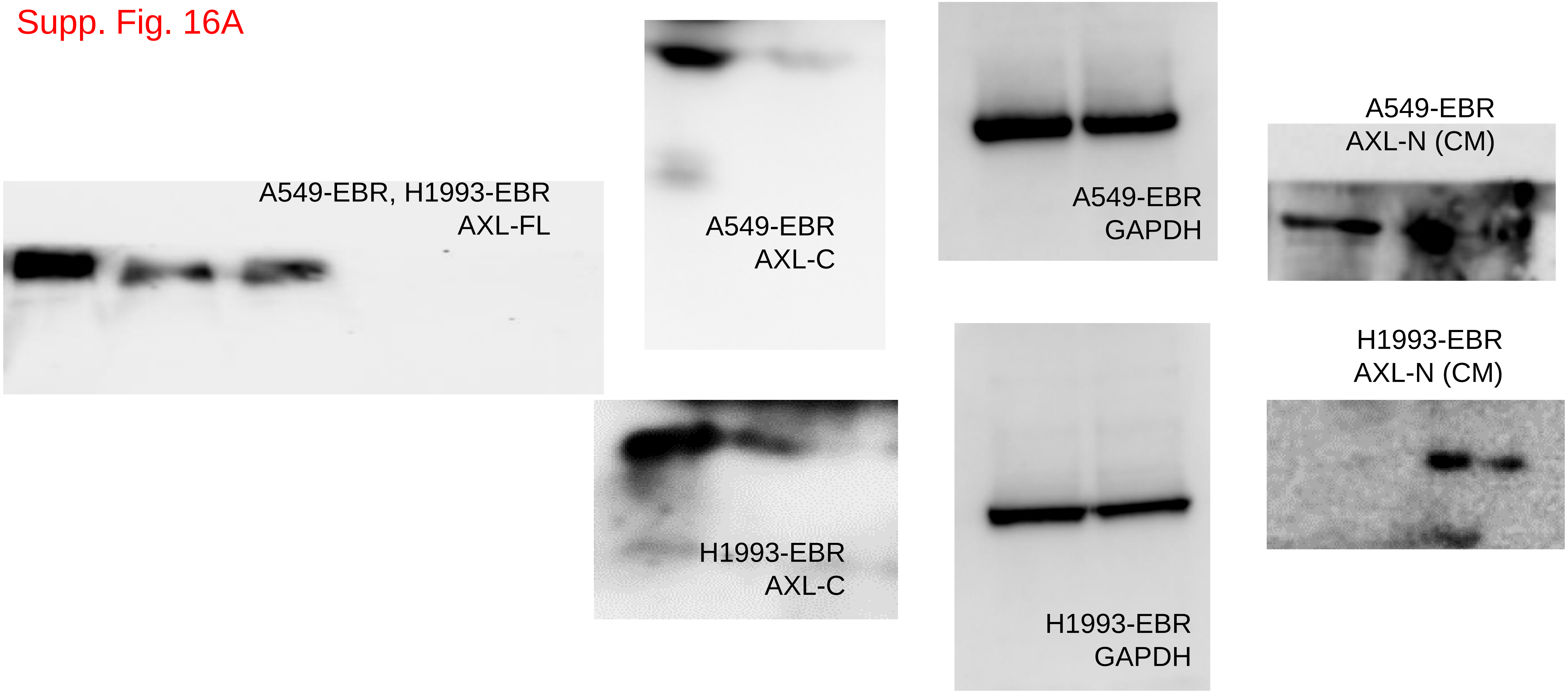
A549-EBR, H1993-EBR
AXL-FL
A549-EBR
AXL-C
H1993-EBR
AXL-C
A549-EBR
GAPDH
H1993-EBR
GAPDH
A549-EBR
AXL-N (CM)
H1993-EBR
AXL-N (CM)

Supp. Fig. 18A

A549-PTXR,-EBR
AXL

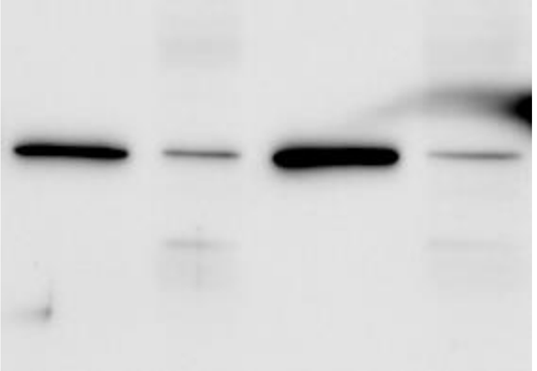

H1993-PTXR,-EBR
AXL

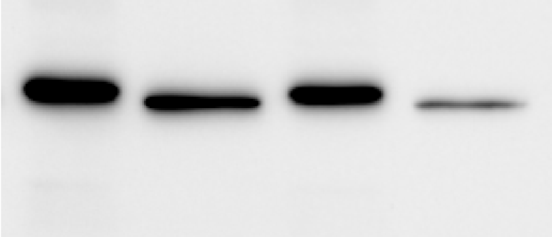

PC9-EBR
AXL

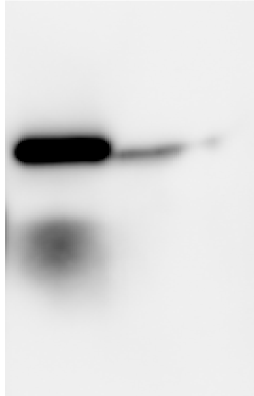

A549-PTXR,-EBR
Actin

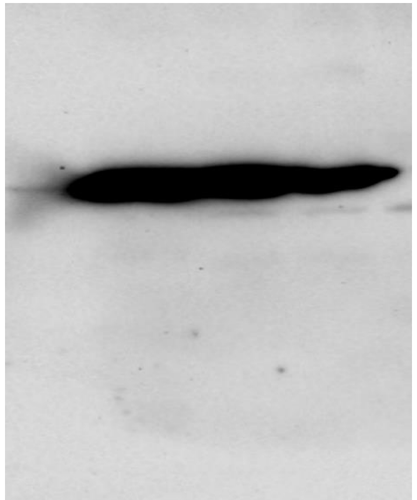

H1993-PTXR,-EBR
Actin

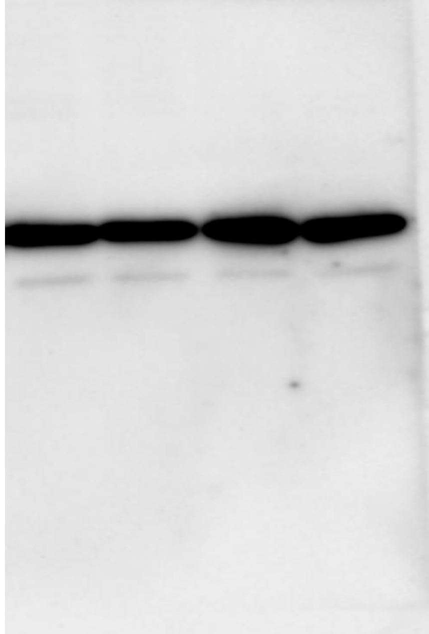

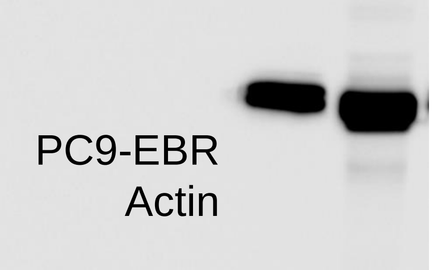

Supplement: Supplementary file 1 — Supplementary Information 1. [file 41598_2021_87599_MOESM1_ESM.pdf]
